# Transcriptomics analysis of *Psidium cattleyanum* Sabine (Myrtaceae) unveil potential genes involved in fruit pigmentation

**DOI:** 10.1590/1678-4685-GMB-2019-0255

**Published:** 2020-04-27

**Authors:** Nicole M. Vetö, Frank Guzman, Franceli R. Kulcheski, Ana Lúcia A. Segatto, Maria Eduarda G. Lacerda, Rogerio Margis, Andreia C. Turchetto-Zolet

**Affiliations:** 1 Universidade Federal do Rio Grande do Sul, Instituto de Biociências, Departamento de Genética, Programa de Pós-graduação em Genética e Biologia Molecular, Porto Alegre, RS, Brazil.; 2 Universidade Federal do Rio Grande do Sul, Centro de Biotecnologia e Programa de Pós-Graduação em Biologia Celular e Molecular, Porto Alegre, RS, Brazil.; 3 Universidade Federal de Santa Catarina, Departamento de Biologia Celular, Embriologia e Genética, Programa de Pós-graduação em Biologia Celular e o Desenvolvimento, Florianópolis, SC, Brazil.; 4 Universidade Federal de Santa Maria, Departamento de Bioquímica e Biologia Molecular, Santa Maria, RS, Brazil.; 5 Universidade Federal do Rio Grande do Sul, Departamento de Biofísica, Porto Alegre, RS, Brazil.; 6 Instituto Nacional de Innovación Agraria, Dirección de Recursos Genéticos y Biotecnología, Lima, Peru.

**Keywords:** Anthocyanin, Atlantic Forest, carotenoids, fruit ripening, Myrtaceae

## Abstract

*Psidium cattleyanum* Sabine is an Atlantic Forest native species that presents some populations with red fruits and others with yellow fruits. This variation in fruit pigmentation in this species is an intriguing character that could be related to species evolution but still needs to be further explored. Our goal was to provide genomic information for these morphotypes to understand the molecular mechanisms of differences in fruit colour in this species. In this study, we performed a comparative transcriptome analysis of red and yellow morphotypes of *P. cattleyanum*, considering two stages of fruit ripening. The transcriptomic analysis performed encompassing leaves, unripe and ripe fruits, in triplicate for each morphotype. The transcriptome consensus from each morphotype showed 301,058 and 298,310 contigs from plants with yellow and red fruits, respectively. The differential expression revealed important genes that were involved in anthocyanins biosynthesis, such as the anthocyanidin synthase (ANS) and UDP-glucose:flavonoid-o-glucosyltransferase (UFGT) that were differentially regulated during fruit ripening. This study reveals stimulating data for the understanding of the pathways and mechanisms involved in the maturation and colouring of *P. cattleyanum* fruits and suggests that the ANS and UFGT genes are key factors involved in the synthase and pigmentation accumulation in red fruits.

## Introduction

**Figure 1:**
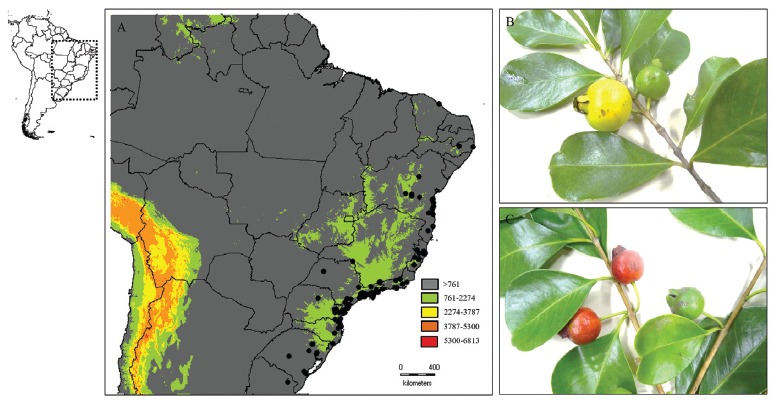
Geographic distribution of *P. cattleyanum* and the morphological differences in their fruits. Map showing the natural distribution of *P. cattleyanum* in the Atlantic Forest (A). Data retrieved from the Global Biodiversity Information Facility (GBIF https://www.gbif.org/) and SpeciesLink (http://splink.cria.org.br/). Fruits of the yellow (B) and red (C) morphotypes of *P. cattleyanum*.

**Figure 2:**
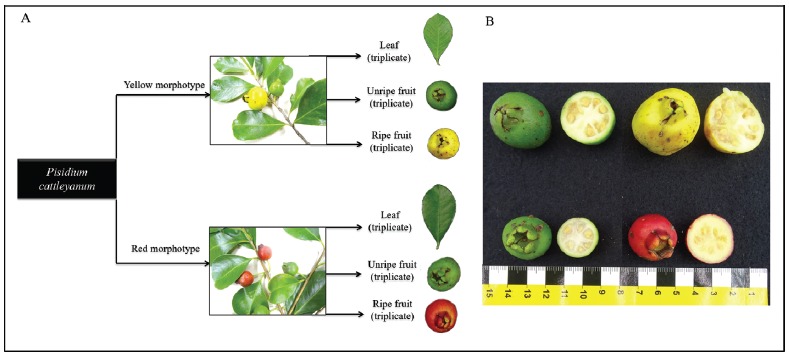
RNA library scheme for transcriptome sequencing of the leaf and fruit tissues of the yellow and red morphotypes (A). Epicarp and endocarp characteristics of the yellow and red morphotypes of *P. cattleyanum* (B).

*Psidium cattleyanum* Sabine (araça, cattley guava, strawberry guava or cherry guava) occurs in the Atlantic Forest, from Bahia to the northeastern Uruguay (Castro *et al.*, 2004) (Figure 1A). The fruits of *P. cattleyanum* have a distinct colour pattern (Figures 1B and C):the epicarp can be yellow or red, and the endocarp shows a light yellow to white or red colour, whitening towards the centre (Sanchotene, 1989) (Figure 2A and B). The colour diversity of *P. cattleyanum* fruits can be found in different locations, where groups of individuals present yellow fruits and others present red fruits. This variation in fruit colour can be an essential ecological aspect not only by the potential dispersers but also by the environmental conditions that influence the species adaptation. Plants with red and yellow fruits characterise two distinct groups according to fruit morphology, as well as other characteristics such as leaf morphology and habit (Souza and Sobral, 2007). Despite these differences, both types are grouped in the same taxonomic clade and have been classified as different morphotypes within the same species. Studies of the molecular basis of fruit colour in this species will be very interesting to help understanding if the differences between the two morphotypes could be related to evolutive and taxonomic aspects or are just polymorphisms among populations.

Fruit colour is determined by different proportions of tissue pigments such as carotenoids, chlorophyll, anthocyanins and flavonoids (Liu *et al.*, 2003; Nashilevitz *et al.*, 2010; Kachanovsky *et al.*, 2012). The determination of this colour pattern is frequently correlated to attracting the animals that will consume these fruits and consequently disperse their seeds (Snow, 1971; Wang *et al.*, 2015). As red and black colours are often related to bird dispersal syndrome, their abundance could be a consequence of a global bird preference for these colours (Schaefer *et al.*, 2007). The red and black colour are mainly a result of the accumulation of anthocyanins, that are specialised metabolites of the phenylpropanoid pathway widely present in plant species, and one of the main compounds responsible for fruit colouration. Anthocyanins are glycosylated polyphenolic compounds with a range of colours ranging from orange, red and purple to blue in flowers, seeds, fruits and vegetative tissues (Tanaka *et al.*, 2008). Tissue accumulation and allocation of anthocyanins are governed by complex metabolic pathways that are regulated by genetic and environmental conditions; and strongly correlated with structural and regulatory gene expression (Jaakola, 2013). Therefore, differences in fruit colouration between species may occur either by modifications in structural genes or by alterations in regulatory genes providing differences in expression of structural genes.

The genes involved in the synthesis of anthocyanins are well studied on model plants and plants with commercial potential. But little is known about the molecular and genetic mechanisms of fruit colour in non-model and native plants. In this regard, the use of a transcriptomic approach may be a useful strategy to identify potential genes involved in fruit pigmentation in *P. cattleyanum* yellow and red morphotypes, thus contributing to the understanding of fruit colourationin this species. This method has been useful in revealing genes associated with the distinctive polymorphism that is common in nature, as well as in screening genome-wide gene expression at any time and in any tissue. Transcriptome analyses have helped to reveal the genetic basis of anthocyanin polymorphism in *Parrya nudicaulis* (Butler *et al.*, 2014). These approach has also been useful to identify candidate genes responsible for pollinator attraction and reproductive isolation in hybridising bromeliad species (Palma-Silva *et al.*, 2016), as well as, to detect the potential genes involved in the terpenoid biosynthesis pathway and terpene diversity in *Eugenia uniflora* L. (Guzman *et al.*, 2014).

In this study, the RNA sequencing and *de novo* assembly of *P. cattleyanum* fruit and leaf transcriptomes were performed for the first time, producing large expression datasets for functional and molecular analyses. Our hypothesis is that distinct molecular mechanisms are involved in the colouring diversity of *P. cattleyanum.* Differential gene expression analysis on the leaves and unripe and ripe fruits of two different morphotypes of *P. cattleyanum* cultivated at the same location was performed to (i) provide reference transcriptomes for the yellow and red morphotypes of *P. cattleyanum*; (ii) to characterise the transcripts of the fruits and leaves, identifying functional category composition by tissues and morphotypes; and (iii) to identify and annotate the structural and regulatory genes involved in the biosynthesis of fruit pigmentation. Aside from that, the transcriptome sequences of *P. cattleyanum* will facilitate future studies on other Myrtaceae species that lack genomic data.

## Material and Methods

### Plant material, RNA isolation and transcriptome sequencing

*Y*oung leaves, unripe and ripe fruit samples were collected from plants of the yellow and red morphotypes (Figure 2A). The unripe and ripe stages of the fruits were defined based on their colour, beeing green for the unripe samples; and yellow or red (according to the respective yellow or red morphotype) for the ripe fruits (Figure 2B). Samples were immediately frozen in liquid nitrogen and stored at -80°C until RNA isolation.

The total RNA was isolated from the leaves, unripe and ripe fruits using a Direct-zol RNA kit (Zymo, USA). Only the mesocarp and epicarp of the fruits were used for RNA isolation. Nine libraries were prepared each morphotype using biological triplicates for each tissue (Figure 2A). Qualitative and quantitative estimations of the RNA were performed using 1.2% agarose gel electrophoresis and Nano-Drop ND-1000 UV-Vis spectrophotometer analysis, respectively. Library preparation and sequencing was performed by Macrogen Inc. (Seoul, Republic of Korea), following the TruSeq Stranded sample preparation protocol. The libraries were individually barcoded and sequenced, together with paired-end strand-specific sequencing in a HiSeq 4000 next-generation sequencing platform.

### ***De novo* transcriptome assembly**

The presence of adapters and the quality of the reads produced by the sequencing were determined for each library using FastQC software (http://www.bioinformatics.babraham.ac.uk/projects/fastqc/). Based on this data, Trinity-v2.6.5 Release (Grabherr *et al.*, 2011) was used to eliminate bases with a Phred quality value below 30, as well as the adapter sequences present in the reads. Cleaned reads were assembled using Trinity software (Grabherr *et al.*, 2011), with three different k-mers (21, 25 and 31) and default parameters for each morphotype. The total transcripts obtained in each separate assembly were merged to produce a combined assembly using the CD-HIT-EST software, with a minimum of 95% of similarity among the assembled transcripts. After this step, we obtained consensus transcripts with a minimum length of 180 bp. To validate the quality of the consensus transcripts, we used BUSCO software (Simão *et al.*, 2015) to identify ortholog groups between the Viridiplantae and the Eukarya. Other parameters, including the overall number of contigs, the average length of the contigs and the N50 value, were obtained using QUAST version 2.3 (Gurevich *et al.*, 2013). Finally, one consensus transcriptome was assembled for each morphotype.

### Annotation of gene families, protein domains and functional classification

The unigenes were annotated using the Blast2GO suite. All sequences were compared with non-redundant sequences of the Viridiplantae from the National Center for Biotechnology Information (NCBI, http://www.ncbi.nlm.nih.gov/) database using BLASTX, with an E-value cutoff of 10-5e. The best hits of each unigene with the highest sequence similarity were chosen to associate the annotations. A functional category assignment for each unigene was conducted using the GOslim tool from the Blast2GO suite, and classification was performed according to GO terms in the molecular functions, biological processes and cellular components. The WEGO online tool was used for graphical representation of the GO terms at the macrolevel (Ye *et al.*, 2006). The identification of gene families and protein domains was performed using the InterProScan tool from the Blast2GO suite from multiple databases, including Gene3D, PANTHER, Pfam, PIR, PRINTS, ProDom, ProSITE, SMART, SUPERFAMILY and TIGERFAM.

### Pathway assignment with the Kyoto Encyclopedia of Genes and Genomes (KEGG)

Pathway mapping of the unigenes using the KEGG database (http://www.genome.jp/kegg/) was performed with the Blast2GO suite. The unigenes were annotated with the KEGG database to obtain their enzyme commission (EC) number. This code was further used to map the unigenes to the KEGG biochemical pathways.

### Differential expression analysis

The cleaned reads of the nine RNAseq libraries for each morphotype were anchored, allowing three mismatches in their respective consensus transcriptomes, using Bowtie. Transcript clusters were generated, as well as their count tables, using the alignment files of each individual in the Corset programme (Davidson and Oshlack, 2014). To perform the statistical analysis for identifying the differential expression, the cluster counting tables were analysed in the DESeq2 package v.1.12.3 of the Bioconductor, using a false discovery rate of 0.001 and a log 2 fold change of 4. For each comparison of the treatments, all differentially expressed genes were separated into two groups, one formed by upregulated clusters and the other by down-regulated clusters. The annotations of each differentially expressed gene were obtained using the longest transcript of each cluster and its respective blast2go annotation.

### Phylogenetic analysis of candidate genes involved in anthocyanin biosynthesis

The phylogenetic analysis was performed with the anthocyanidin synthase (ANS, same as leucocyanidin oxygenase LDOX) and UDP-glucose:flavonoid-3-O-glycosyltransferase (UFGT) gene families from the red and yellow morphotypes and other species, such as *Vitis vinifera*, *Eucalyptus grandis*, *Solanum lycopersicum* and *Prunus persica* (only for ANS phylogeny). The unigenes of *P. cattleyanum* previously identified by BLAST, containing the candidate ANS and UFGT, were first examined in ORF Finder (http://www.ncbi.nlm.nih.gov/gorf/gorf.html) to determine the open reading frame of each unigene and to predict protein sequences. The conserved domains of each predicted protein were identified using the search tool in the SMART protein database (http://smart.embl-heidelberg.de/). Then, protein sequence alignments for each gene family were performed using MUSCLE (Edgar 2004), implemented in MEGA 7 (Kumar *et al.*, 2016), using the default parameters. The alignments were then used for phylogenetic reconstruction using Bayesian analysis in BEAST v.1.8.4 software (Drummond *et al.*, 2012). The best fit model of protein evolution was the LG+I+G model for the ANS sequences and the JTT+G+F model for the UFGT sequences, which were selected using PROTTEST3 (Abascal *et al.*, 2005). The birth-death process was chosen as a tree prior to Bayesian analysis, and 50,000,000 generations were performed using Markov chain Monte Carlo algorithms. Two independent runs were carried out to confirm the chain convergence. TRACER 1.6 (http://tree.bio.ed.ac.uk/software/tracer/) was used to check for convergence of the Markov chains and adequate effective sample sizes (>200). FIGTREE v.1.4.1 software (http://tree.bio.ed.ac.uk/software/figtree/) was used to visualise and edit the trees.

## Results

### Characterisation of plant material in yellow and red morphotypes

The ripening processes started and ended at the same time for both morphotypes. The colour of the fruit peel changed gradually, from green to yellow and red, with the progression of ripening in the respective morphotypes (Figure 2B). The pulp colour presented moderate changes in its intensity, from white to whitish-yellow and red in the respective morphotypes. Fruit firmness decreased according to the colour change.

### **Sequencing and *de novo* assembly**

To obtain the *P. cattleyanum* transcriptome, libraries were constructed and sequenced from the leaves and fruit during two ripening stages (Figures 2A and B). The 403,311,198 and 396,182,350 (yellow and red morphotypes, respectively) overall raw reads obtained from the Illumina sequencing were trimmed down to 379,508,632 (94.10%), and 366,766,700 (92.58%) reads using the Trimmomatic tool (Table S1), representing the high quality of the sequencing. A consensus transcriptome from each morphotype was assembled, using multiple k-mers, which were further merged with CD-HIT-EST into 301,058 and 298,310 contigs (Table 1). The mean transcript sizes were 889 (yellow morphotype) and 866 (red morphotype) bp, with lengths ranging from 180 (both) to 13,213 and 12,416 bp for the yellow and red morphotypes, respectively (Table 1), and 49.17% and 49.44% of the GC content for the yellow and red morphotypes, respectively, considering the means of the nine libraries for each morphotype (Table S1). The averages N50 were 1511 bp (yellow morphotype) and 1471 bp (red morphotype). Among the total distribution of non-redundant contigs, most of the transcripts had between 401 and 4000 bp, although a large number of transcripts were shorter than 400 bp, as presented in Figure S1.

**Table 1:** Summary of transcripts generated for *Psidium cattleyanum* yellow and red morphotype

Assembly Yellow	k21	k25	k31	Merged assembly
Number of transcripts	251522	294213	282060	301058
Median transcript length	558	467	494	534
Mean transcript length	940	788	826	889
Max transcript length	13213	11646	11719	13213
No. transcript 1kbp	80335	73757	77395	89929
N50	1624	1305	1382	1511
**Assembly Red**	**k21**	**k25**	**k31**	**Merged assembly**
Number of transcripts	247694	310159	265241	298310
Median transcript length	545	419	474	510
Mean transcript length	944	697	796	866
Max transcript length	12416	12344	12342	12416
No. transcript 1kbp	78272	63327	67945	84845
N50	1658	1096	1328	1471

### Functional annotation and characterisation

**Figure 3:**
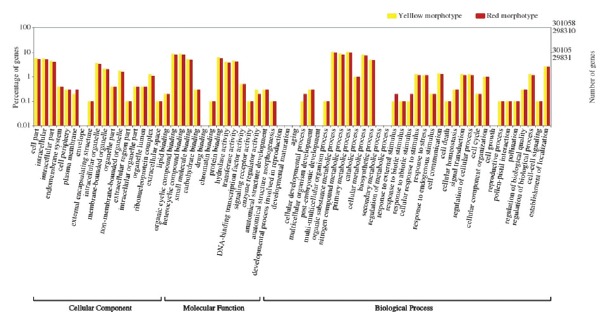
Functional annotation and comparison of the GO terms among the leaf and unripe and ripe transcriptome sequences in the yellow and red morphotypes of *P. cattleyanum*.

The results of unigene validation and annotation showed that 92,354 (32.66%) out of 282,758 unigenes showed significant similarities to known proteins in the Nr database for the yellow morphotype, with 86,427 (32.35%) out of 267,180 for the red morphotype (Table 2). Gene ontology (GO) enrichment analysis of the 94,800 and 87,471 unigenes showed associations with known proteins of biological, cellular and functional GO classes (Table 3, Figure 3). Possible functions of transcripts with significant BLAST hits were classified for three main GO terms: biological process (59,582 and 55,427 unigenes for yellow and red morphotypes, respectively); cellular component (26,584 and 24,840 unigenes for yellow and red morphotypes, respectively); and molecular function (83,206 and 76,607 unigenes for yellow and red morphotypes, respectively) (Figure 3). A total of 18,020 and 16,930 transcripts were identified and were included in 129 and 130 KEGG pathways, for the yellow and red morphotypes, respectively (Table 2).

**Table 2:** Statistics of BLAST2GO annotation of assembled unigenes of Psidium cattleyanum yellow and red morphotype

Annotation	***Psidium cattleyanum* morphotypes**	
	Yellow	Red
Number of unigenes	282768	267180
Unigenes with BLASTX hits	92354	86427
Unigenes without BLASTX hits	190414	180753
Unigenes with GO terms	94800	87471
Unigenes with Enzyme (EC)	18020	16930
Unigenes with KEEG pathways	12419	12064
KEEG pathway	129	130

**Table 3:** Gene ontology (GO) enrichment analysis

	***Psidium cattleyanum* morphotypes**		
	Yellow	Red	Total
Contigs	301058	29831	599368
Annotated contigs	94800	87471	182271
Biological	59582	55427	115009
Cellular	26584	2484	51424
Function	83206	76607	159813
Total	169372	156874	326246

### Differential gene expression in the leaves, unripe fruit and ripe fruit of the yellow and red morphotypes

**Figure 4:**
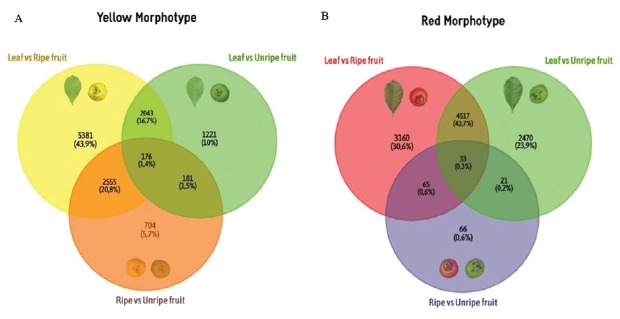
Total number of differentially expressed clusters among the leaf and unripe and ripe fruits from the yellow and red morphotypes of P.cattleyanum.

**Figure 5:**
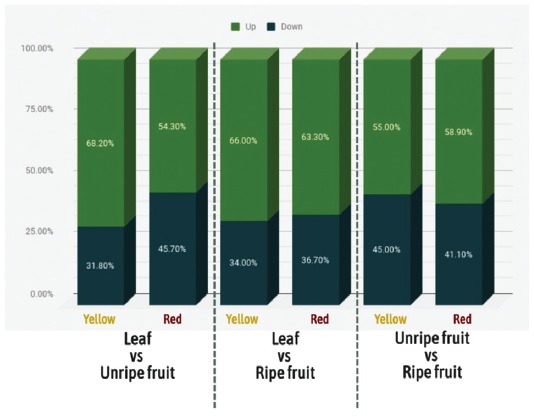
Total clusters upand down-regulated in each comparison of leaf vs unripe fruit, leaf vs ripe fruit and unripe vs ripe fruit of each morphotype of *P. cattleyanum*.

Differential gene expression was analysed in leaf vs ripe fruit, leaf vs unripe fruit and ripe vs unripe fruit, for each morphotype (Figures 4 and 5, Table S2). This analysis allowed the identification of 3,621 and 7,041 clusters, comparing leaf with unripe fruit of the yellow and red morphotypes, respectively. Comparing leaf with ripe fruit, 10,155 and 7,775 clusters were found (yellow and red morphotypes, respectively; Figure 4). Annotation of the differentially regulated genes revealed that genes such as pleiotropic drug resistance 2, galactinol synthase 1 and rust resistance kinase Lr10, among others, were up-regulated, and (R,S)-reticuline 7-O-methyltransferase, aluminum-activated malate transporter 4, and monosaccharide-sensing 2, among others, were down-regulated in both the leaf vs unripe fruit and leaf vs ripe fruit of the yellow morphotype. Some genes, such as hydroquinone glucosyltransferase, were up-regulated only in the leaf vs unripe fruit comparison, and others, such as (-)-germacrene D synthase, only in the leaf vs ripe fruit comparison. Annotation of the differentially regulated genes in the red morphotype revealed that genes such as ribulose bisphosphate carboxylase oxygenase chloroplastic, ferredoxin-dependent glutamate chloroplastic, and plastocyanin, among others, were up-regulated, while expansin 3, expan sin-A8-like precursor, and isoflavone 3-hydroxylase were down-regulated (Table S3).

**Figure 6:**
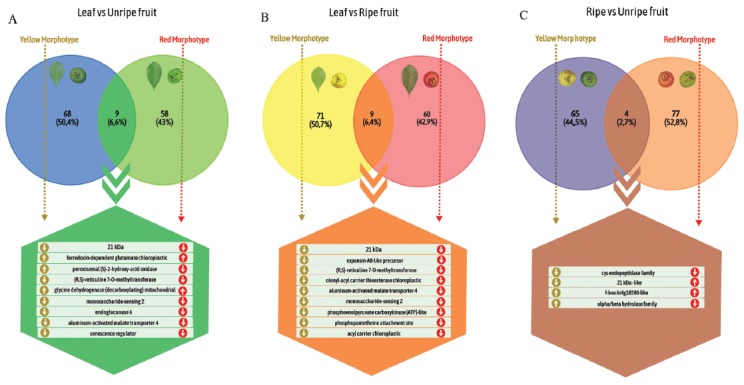
Venn diagrams showing clusters shared among the different tissues of each morphotype of *P. cattleyanum*. Each comparison displays the annotation shared. The arrows indicate the different regulation (upand down-regulated) for each morphotype.

Differential gene expression analysis revealed a total of 3,616 and 185 clusters for the unripe vs ripe fruit of the yellow and red morphotypes, respectively. Annotation of the differentially regulated genes revealed that specific genes, such as F-box-like, were up-regulated in both morphotypes. The Cys endopeptidase family was down-regulated in both morphotypes. Taking into account the top 100 expressed genes (Tables S4 and S5), the four common clusters found in the differential expression analysis of the unripe and ripe fruit of the two morphotypes represented only 2.7%, as shown in Figure 6C. Potential structural and regulatory genes of anthocyanin biosynthesis were found among the differentially expressed genes during the ripening processes of the red fruit. Among the putative homologs of the structural genes, we found two clusters containing putative phenylalanine ammonia-lyase (Psi-rd-282061 and Psi-rd75320), one cluster containing putative chalcone isomerase (Psi-rd-256261), one containing putative leucoanthocyanidin dioxygenase (ANS, Psi-rd-165087) and one containing putative UFGT (Psi-rd-65618). The comparison of unripe vs ripe fruit showed all these clusters to be down-regulatedwhile other clusters containing putative leucoanthocyanidin dioxygenase (ANS, Psi-rd-159291) were up-regulated. Regarding regulatory genes, a b-ZIP transcription factor 2-like (Psi-rd-297933) was up-regulated. Among all the differentially expressed genes in the yellow fruit, we identified anthocyanidin 3-O-glucosyltransferase 2-like (Psi-yw292910), anthocyanidin 3-O-glucosyltransferase 7, anthocyanidin 5,3-O-glucosyltransferase, and anthocyanin regulatory C1 as up-regulated genes and anthocyanidin 3-Oglucosyltransferase 2 as a down-regulated gene.

**Figure 7:**
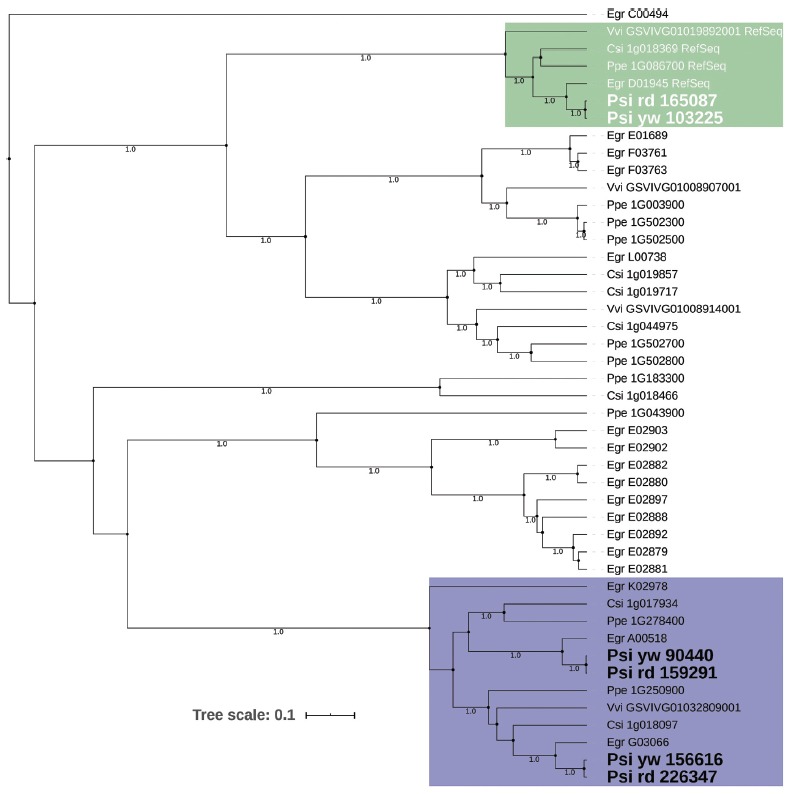
Phylogenetic analysis showing the principal putative homologs of ANS of *P. cattleyanum* grouped with the reference sequence of other species. The posterior probabilities greater than 0.95 are shown on branches.

Putative homologs of ANS and UFGT genes were identified in more than 100 differentially expressed clusters in the red morphotype, denoting metabolic differences in fruit colour between the morphotypes. Some ANS and UFGT homologs are crucial in the final step of anthocyanin formation. Thus, phylogenetic analysis was performed with putative homologs of each ANS and UFGT identified in *P. cattleyanum* and sequences of these gene families from other species (Tables S6 and S7). Putative homologs of these genes were searched in all differentially expressed clusters in all comparisons of the *P. cattleyanum* red and yellow morphotypes, resulting in the identification of further putative ANS gene (Psi-rd-226347), which the comparison of leaf vs unripe fruit found to be up-regulated. For the yellow morphotype, three clusters with putative ANS genes (Psiyw-156616, Psi-yw-103225 and Psi-yw-90440) were identified, but none of these presented higher expression in the ripe fruit. The phylogenetic analysis of ANS genes revealed the formation of three distinct groups (Figure 7). The first group included clusters Psi-rd-165087 and Psi-yw-103225 in the red and yellow morphotypes, respectively, together with the well-characterised ANS sequences of *E. grandis*, *V. vinifera*, *C. sinensis* and *P. persica*. The other two groups included the remaining sequences that also belong to the 2oxoglutarate-dependent dioxygenase gene family but are not related to anthocyanin biosynthesis. This result suggests that the clusters Psi-rd-159291, Psi-rd-226347, Psi-yw-90440, and Psi-yw-156616 are not involved in anthocyanin biosynthesis. Interestingly, the closely related clusters Psi-yw103225 (yellow morphotype) and Psi-rd-165087 (red morphotype) did not present the same expression pattern.

**Figure 8:**
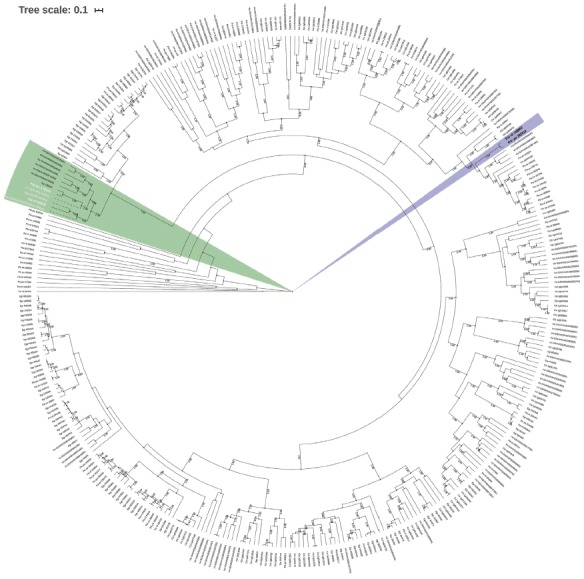
Phylogenetic analysis showing the putative homologs of UFGT of *P. cattleyanum* grouped with genes knowns in other species involved with anthocyanin synthesis. The posterior probabilities greater than 0.95 are shown on branches.

Regarding the UFGT genes, 32 clusters in the yellow morphotype and 54 in the red in all differentially expressed genes were identified. Given that the UFGT genes belong to a multi-membered gene family, the phylogeny revealed that the Psi-rd-65618 and Psi-yw-172184 clusters were grouped with genes already known to be involved with anthocyanin synthesis. Interestingly, the red Psi-rd-65618 cluster was closely related with *E. grandis* and *V. vinifera*, while the yellow Psi-yw-172184 cluster was closely related with *C. sinensis* (Figure 8). The cluster of UFGT genes found to be differentially expressed in the yellow fruit in the top 100 expressed genes did not occur in the same phylogenic group. This suggests that only the Psi-rd-65618 cluster of the red morphotype is involved in anthocyanin biosynthesis.

## Discussion

The evolution of fruit characteristics, such as colour, is considered an essential driver for diversification in many groups of angiosperms (Lomáscolo *et al.*, 2010; Valenta *et al.*, 2018). The material basis of fruit colour formation is pigment accumulation, and the type and level of these accumulations determine the colour and shade of the fruit. Chlorophyll, carotenoid, anthocyanidins and other flavonoids are the primary pigments that determine fruit colour. The levels of flavonoids in the *P. cattleyanum* red morphotype were found to be usually higher than in the *P. cattleyanum* yellow morphotype (Biegelmeyer *et al.*, 2011), mainly because of the presence of cyanidins in the red fruits - the main pigments in bright-red-coloured fruits (Macheix *et al.*, 1990; Jaakola *et al.*, 2002). The genetic background of the species/lineage and environmental factors determine which anthocyanins occur in fruit, as well as their concentrations. Thus, the variation in fruit colour in *P. cattleyanum* may be associated with selective pressure on the genes involved in this pigment biosynthesis and accumulation (Carbone *et al.*, 2009). Information on the molecular basis of fruit colour in this species could be extremely useful in understanding the evolution and diversity of fruit colours in nature. In this context, transcriptome analysis could be interesting, since it can be applied to the study of species with incomplete or no genome sequence resources, allowing the identification of genes that are related to pigment biosynthesis and accumulation in non-model plants. In this study, transcriptome sequences of the yellow and red morphotypes of *P. cattleyanum* were assembled for the first time to analyze the global gene expression among the leaves, non-ripe and ripe fruits of each morphotype. More than 300,000 and 290,000 contigs were obtained, and 280,000 and 260,000 were annotated for the yellow and red morphotypes, respectively. The results of the sequencing and transcriptome assembly suggest that the unigene data were reliable for use in further analysis.

In the later ripening stages, *P. cattleyanum* fruits undergo colour changes from green to yellow in the yellow morphotype and green to red in the red morphotype. The red colour is probably a result of an increase in anthocyanins, consisting mostly of cyanidin-3-glucoside (Dalla Nora *et al.*, 2014). Studies that have phytochemically compared and quantified the two morphotypes have revealed that there is a higher accumulation of anthocyanins in the red fruit than in the yellow fruit (Luximon-Ramma *et al.*, 2003; Fetter *et al.*, 2010; Vinholes *et al.*, 2015; Pereira *et al.*, 2017). Several genes involved in anthocyanin biosynthesis were found to be upand down-regulated in the red fruit, but not in the yellow fruit. For instance, a basic leucine zipper (bzip) transcription factor was up-regulated, while the UDP-glucose:flavonoido-glucosyltransferase was down-regulated. A previous study has demonstrated that the transcription factor bzip (HY5) promotes anthocyanin accumulation by regulating the expression of the MdMYB10 gene and downstream anthocyanin biosynthesis genes in apple (An *et al.*, 2017). The UDP-glucose:flavonoid-o-glucosyltransferase gene has been demonstrated to regulate anthocyanin biosynthesis during fruit colouration (Zhao *et al.*, 2012). Other studies on crop species of apple, pear and grape have reported that the expression of anthocyanin biosynthesis genes correlates with the fruit anthocyanin content (Feng *et al.*, 2010, 2013; Liu *et al.*, 2013; Soubeyrand *et al.*, 2014).

Apart from the distinct expression profiles of the genes involved in anthocyanin biosynthesis between the red and yellow morphotypes studied here during fruit ripening, the general expression profile of each morphotype was quite different in all comparisons (Figure 6). The differential gene expression analysis revealed that less than 10% of the top 100 differentially expressed genes (see Tables S3, S4, S5, S8, S9 and S10) in all tissue comparisons were common between the two morphotypes (Figure 6). These results showed a different transcriptional profile, especially concerning the formation of pigmentation, suggesting marked distinctions between the two morphotypes regarding gene regulation. These results corroborate with our hypothesis that distinct molecular processes are involved in fruit pigmentation of each morphotype. When the transcriptomes data were compared between the leaves and fruits (unripe and ripe) of each morphotype, we observed differentially expressed genes that could be significantly involved in specific organ/tissue development, such as galactinol synthase, and a key enzyme in the raffinose family of oligosaccharide (RFOs) biosynthesis in plants (Sengupta *et al.*, 2015). The RFOs have been reported as playing a fundamental role in plants. They protect the embryo from maturation-related desiccation (Downie *et al.*, 2003) and in several cellular functions (Stevenson *et al.*, 2000), are predominant carbohydrate transporters in some plant species, and can be accumulated in vegetative tissues in response to several abiotic factors (Hincha *et al.*, 2003).

Genes involved in the biosynthesis of anthocyanins in model species have been extensively studied, such as in petunia, *Arabidopsis,* and maize, and it has been demonstrated that two groups of genes were essential. One of these includes structural genes that encode enzymes that are directly involved in the synthesis of anthocyanin and other flavonoids, such as chalcone synthase (CHS) (Montefiori *et al.*, 2011), chalcone isomerase (CHI) (Goto-Yamamoto *et al.*, 2002), dihydroflavonol-4-reductase (DFR) (Tsuda *et al.*, 2004), leucoanthocyanidin dioxygenase (ANS) (YE *et al.*, 2017) and glucosyltransferase (UFGT) (Kobayashi *et al.*, 2001). The other group of genes includes regulatory genes. The regulation of gene expression at the transcriptional level is considered to be a critical mechanism in the regulation of pigment biosynthesis and accumulation. Some examples include the MYB, basic helix-loop-helix (bHLH) and WD40 transcription factors. These proteins form MYB-bHLH or MYB-bHLH-WD40 (MBW) complexes that bind to the promoter region of biosynthetic genes, regulating their expresion (Dare *et al.*, 2008). Our phylogenetic analysis allowed the identification of the genes of the yellow and red morphotypes that grouped with the well-characterized ANS homologs of other species that act in anthocyanin biosynthesis.

Interestingly, the expression pattern of these genes (Psi-yw-103225, Psi-rd-165087) was different in each morphotype, reinforcing their possible role in fruit colour differentiation. In the case of UFGT, only the gene of the red morphotype (Psi-rd-65618) grouped with genes already known to be involved with anthocyanin synthesis, suggesting that the homologs found in the yellow morphotype are not related to anthocyanin biosynthesis. Besides that molecular process, fruit pigmentation is usually responsible for the attraction of seed dispersers because colour patterns facilitate the detection of fruits against their background. Thus, interactions between fruit colour and fruit dispersers may be responsible for driving the evolution of fruit pigmentation in several plant species (Burns, 2005). One explanation for fruit colour variation is that different types of frugivores select for different-coloured fruits (Willson and Melampy, 1983). Suggesting that adaptive mechanisms could be involved in *P. cattleyanum* fruit colour and deserve further consideration of these issues.

In this study, we were able to identify the potential genes involved in the pigmentation of *P. cattleyanum* fruits and showed that the two morphotypes present distinct mechanisms for fruit pigmentation during ripening. The transcriptome analysis of two distinct morphotypes of *P. cattleyanum* yielded a global landscape of differentially expressed genes in a native Neotropical species. The consensus transcriptomes for the yellow and red morphotypes of *P. cattleyanum* will also be highly relevant to other Myrtaceae species, as a reference for comparison. The transcriptomes of the yellow and red morphotypes regarding fruit colour contrast markedly. Apart from that, we found important genes that could be involved in the differentiation of these two morphotypes and highlight the need for functional studies to confirm the function of each of these genes.

## Supplementary material

The following online material is available for this article:

Figure S1 - Length distribution of P. cattleyanum transcripts from the combined assembly.

Table S1 - Statistical summary of sequences of Psidium cattleyanum libraries obtained for yellow and red morphotypes

Table S2 - Total differential gene expression among P. cattleyanum yellow and red morphotype.

Table S3 - Top 100 differential gene expression between Leaf vs Ripe fruit in red morphotype.

Table S4 - Top 100 differential gene expression between Unripe vs Ripe fruit in yellow morphotype.

Table S5 - Top 100 differential gene expression between Unripe vs Ripe fruit in red morphotype.

Table S6 - Species information used to perform UDP-glucose: flavonoid 3-O-glucosyltransferase (UFGT) phylogenetic analysis.

Table S7 - Species information used to perform anthocyanidin synthase (ANS,) phylogenetic analysis.

Table S8 - Top 100 differential gene expression between Leaf vs Unripe fruit in yellow morphotype.

Table S9 - Top 100 differential gene expression between Leaf vs Ripe fruit in yellow morphotype.

Table S10 - Top 100 differential gene expression between Leaf vs Unripe fruit in red morphotype.
