# Supplementary material for: Transcriptomics analysis of Psidium cattleyanum Sabine (Myrtaceae) unveil potential genes involved in fruit pigmentation
Source: Genet Mol Biol. 2020 Apr 27;43(2):e20190255. doi: 10.1590/1678-4685-GMB-2019-0255 (PMC7199922; doi:10.1590/1678-4685-GMB-2019-0255)
Supplement: Figure S1 [file 1415-4757-GMB-43-2-e20190255-s1.pdf]

Supplementary material to: Transcriptomics analysis of *Psidium cattleyanum* Sabine (Myrtaceae) unveil potential genes involved in fruit pigmentation

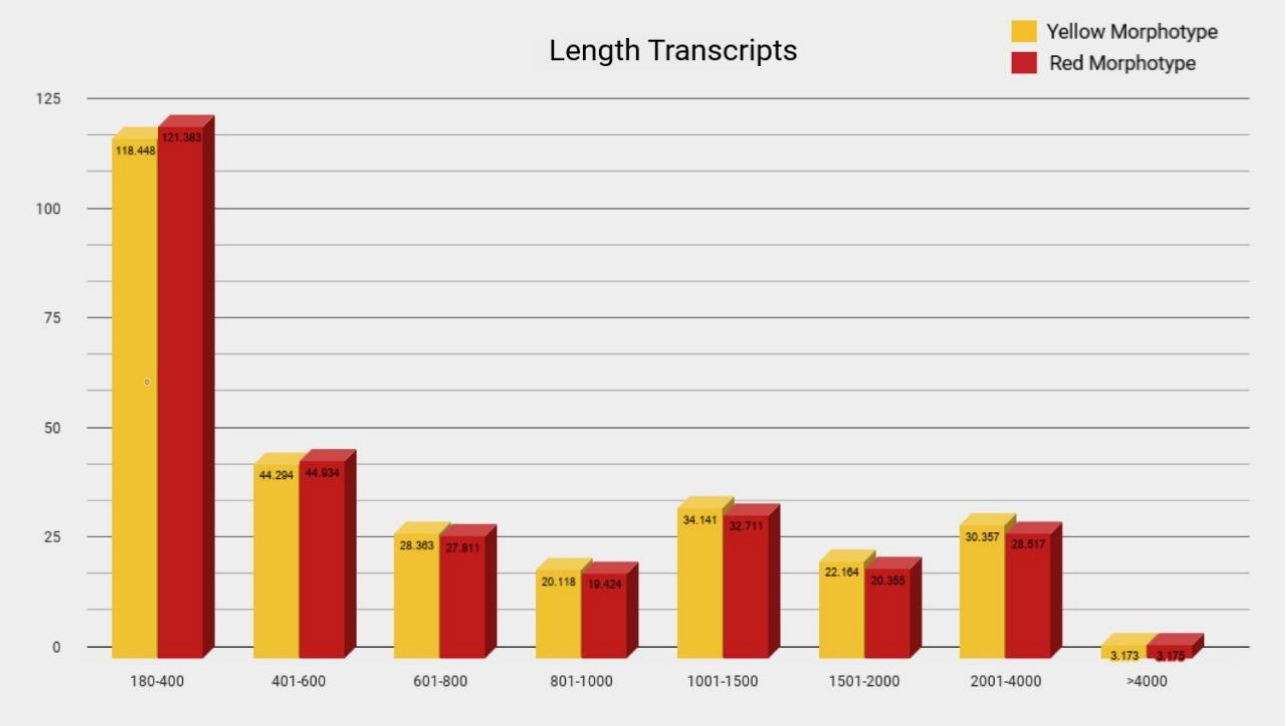

Figure S1 - Length distribution of *P. cattleyanum* transcripts from the combined assembly. The yellow and red bars represent the transcript numbers for the yellow and red morphotypes, respectively.
