# Supplementary material for: Transcriptomics analysis of Psidium cattleyanum Sabine (Myrtaceae) unveil potential genes involved in fruit pigmentation
Source: Genet Mol Biol. 2020 Apr 27;43(2):e20190255. doi: 10.1590/1678-4685-GMB-2019-0255 (PMC7199922; doi:10.1590/1678-4685-GMB-2019-0255)
Supplement: Table S1 [file 1415-4757-GMB-43-2-e20190255-s2.pdf]

Supplementary material to: Transcriptomics analysis of *Psidium cattleyanum* Sabine (Myrtaceae) unveil potential genes involved in fruit pigmentation

Table S1 - Statistical summary of sequences of *Psidium cattleyanum* libraries obtained for yellow and red morphotypes.

	Sample ID	Total raw reads	Total cleaned reads	Raw reads GC(%)	Trimmed reads GC(%)
YELLOW	LEAF 1	45,489,838	42,616,382	50,18	49,00
	LEAF 2	45,284,254	42,882,214	50,34	49,50
	LEAF 3	43,679,922	41,266,246	49,95	49,00
	UNRIPE FRUIT 1	52,046,514	48,820,480	49,68	49,00
	UNRIPE FRUIT 2	43,992,392	41,440,638	49,80	49,00
	UNRIPE FRUIT 3	41,848,404	39,578,246	49,63	49,00
	RIPE FRUIT 1	42,265,556	39,492,114	49,62	49,00
	RIPE FRUIT 2	45,889,476	43,338,934	50,55	50,00
	RIPE FRUIT 3	42,814,842	40,073,378	49,87	49,00
	Total	403,311,198	379,508,632		
RED	LEAF 1	45,242,080	41,785,414	50,59	49,50
	LEAF 2	48,032,056	44,963,030	49,74	49,00
	LEAF 3	48,359,218	45,070,598	49,53	49,00
	UNRIPE FRUIT 1	48,486,364	45,205,874	48,98	48,00
	UNRIPE FRUIT 2	39,817,028	37,256,212	50,67	49,50
	UNRIPE FRUIT 3	38,965,530	33,061,038	49,74	50,50
	RIPE FRUIT 1	35,608,570	33,677,856	51,03	50,50
	RIPE FRUIT 2	48,322,130	45,599,394	50,75	50,00
	RIPE FRUIT 3	43,349,374	40,147,284	49,95	49,00
	Total	396,182,350	366,766,700		
