# Supplementary material for: Transcriptomics analysis of Psidium cattleyanum Sabine (Myrtaceae) unveil potential genes involved in fruit pigmentation
Source: Genet Mol Biol. 2020 Apr 27;43(2):e20190255. doi: 10.1590/1678-4685-GMB-2019-0255 (PMC7199922; doi:10.1590/1678-4685-GMB-2019-0255)
Supplement: Table S2 [file 1415-4757-GMB-43-2-e20190255-s3.pdf]

Supplementary material to: Transcriptomics analysis of *Psidium cattleyanum* Sabine (Myrtaceae) unveil potential genes involved in fruit pigmentation

Table S2 - Total differential gene expression among *P. cattleyanum* yellow and red morphotype.

Comparation	Yellow	%	Red	%
Leaf vs unripe fruit Up	2470	68,2	3822	54,30%
Leaf vs unripe fruit Down	1151	31,8	3219	45,70%
Leaf vs ripe fruit Up	6699	66%	4919	63,30%
Leaf vs ripe fruit Down	3456	34%	2856	36,70%
Unripe vs ripe fruit Up	1989	55%	109	58,90%
Unripe vs ripe fruit Down	1627	45%	76	41,10%
