# Supplementary material for: Transcriptomics analysis of Psidium cattleyanum Sabine (Myrtaceae) unveil potential genes involved in fruit pigmentation
Source: Genet Mol Biol. 2020 Apr 27;43(2):e20190255. doi: 10.1590/1678-4685-GMB-2019-0255 (PMC7199922; doi:10.1590/1678-4685-GMB-2019-0255)
Supplement: Table S3 [file 1415-4757-GMB-43-2-e20190255-s4.pdf]

Supplementary material to: Transcriptomics analysis of *Psidium cattleyanum* Sabine (Myrtaceae) unveil potential genes involved in fruit pigmentation

Table S3 - Top 100 differential gene expression between Leaf vs Ripe fruit in red morphotype.

Cluster	Unigenes	Annotation	log2FoldChange	padj
Cluster-2958.21568	Psi-rd-271456	DNA-damage-repair toleration DRT100-like	-12,18861964	4,158310918E-90
Cluster-972.4	Psi-rd-283089	polygalacturonase	-12,08923805	5,59825423E-140
Cluster-693.17	Psi-rd-272882	isoflavone 3 -hydroxylase	-11,80971154	4,94720859E-163
Cluster-882.1	Psi-rd-268889	probable 9-cis-epoxycarotenoid dioxygenase chloroplastic	-11,75223277	2,80522989E-161
Cluster-2958.1631	Psi-rd-231317	omega-hydroxypalmitate O-feruloyl transferase	-11,61364607	8,11784835E-118
Cluster-899.1	Psi-rd-268423	21 kDa	-11,46268598	2,66190127E-103
Cluster-899.16	Psi-rd-268420	21 kDa	-11,36870743	1,93382506E-287
Cluster-2741.0	Psi-rd-88462	transcription factor PRE5-like	-11,2842145	5,317234211E-99
Cluster-899.4	Psi-rd-76839	21 kDa	-11,24802619	8,43641235E-154
Cluster-2958.24538	Psi-rd-226252	expansin-A8-like precursor	-11,20232491	2,85173127E-164
Cluster-2958.1894	Psi-rd-180356	MADS-box CMB1-like	-10,7115307	9,05686609E-140
Cluster-2958.2139	Psi-rd-270950	(R,S)-reticuline 7-O-methyltransferase	-10,51310735	1,37726526E-137
Cluster-2958.9678	Psi-rd-270096	probable RNA-binding ARP1 isoform X2	-10,40440446	2,181680556E-98
Cluster-2958.24239	Psi-rd-115441	Pollen allergen / Rare lipoprotein A (RlpA)-like double-psi beta-barrel	-10,30758099	1,665705500E-98
Cluster-19403.3	Psi-rd-239217	Gibberellin regulated protein	-10,26811976	2,825280242E-98
Cluster-2958.23328	Psi-rd-294597	expansin 3	-10,26791124	1,50926581E-114
Cluster-2958.24274	Psi-rd-294600	expansin 3	-10,17287878	3,11848309E-183
Cluster-2210.11	Psi-rd-254768	Pectate lyase	-10,12180410	3,37309724E-140

Cluster	Unigenes	Annotation	log2FoldChange	padj
Cluster-5378.2	Psi-rd-255580	ethylene receptor 2	-9,930069419	4,12691716E-120
Cluster-1441.0	Psi-rd-108391	aluminum-activated malate transporter 4	-9,779265092	9,49031820E-110
Cluster-1778.1	Psi-rd-195924	COBRA 7	-9,546406276	2,00158335E-100
Cluster-693.14	Psi-rd-64662	cytochrome P450 family	-9,476259405	1,33265646E-104
Cluster-2958.26748	Psi-rd-208895	Pectate lyase	-9,215381978	1,63829428E-113
Cluster-1806.5	Psi-rd-265758	protein E6	-9,208280161	7,35759578E-121
Cluster-2958.28608	Psi-rd-245235	acyl carrier chloroplastic	-8,790314257	2,376335866E-93
Cluster-2958.26252	Psi-rd-274141	Pectate lyase	-8,664052014	1,08988532E-207
Cluster-1806.7	Psi-rd-61236	protein E6	-8,516863260	6,74635879E-165
Cluster-11399.0	Psi-rd-169722	omega-hydroxypalmitate O-feruloyl transferase-like	-8,409548000	8,862185036E-94
Cluster-3828.2	Psi-rd-265670	Senescence regulator	-7,939081312	1,109244157E-97
Cluster-1244.5	Psi-rd-173010	hypothetical protein EUGRSUZ_E00515	-7,936347344	3,062065680E-99
Cluster-1806.8	Psi-rd-258084	protein E6	-7,884458834	1,77267532E-105
Cluster-18407.6	Psi-rd-195692	ABC transporter G family member 25	-7,875178781	4,428434473E-99
Cluster-2958.24366	Psi-rd-245245	Phosphopantetheine attachment site	-7,839071801	2,99664646E-101
Cluster-18407.11	Psi-rd-247775	ABC transporter G family member 25	-7,401211640	6,51638110E-131
Cluster-2958.24364	Psi-rd-146323	Phosphopantetheine attachment site	-7,356747086	2,751851712E-97
Cluster-2958.15087	Psi-rd-253052	peptide methionine sulfoxide reductase B5-like	-7,350123931	1,085064878E-96
Cluster-593.12	Psi-rd-294992	hydroxymethylglutaryl- reductase	-7,186293415	3,062576979E-95
Cluster-1105.2	Psi-rd-162061	lipid transfer precursor	-7,083723925	1,148287583E-92
Cluster-2958.1687	Psi-rd-39966	phosphoenolpyruvate carboxykinase (ATP)-like	-6,901049324	2,483306043E-90
Cluster-8355.0	Psi-rd-47473	nematode resistance -like HSPRO2	-6,876687789	2,07201899E-108
Cluster-1821.2	Psi-rd-238979	monosaccharide-sensing 2	-6,807916396	3,96350341E-107
Cluster-7025.7	Psi-rd-179602	oleoyl-acyl carrier thioesterase chloroplastic	-6,770887036	1,07223831E-102
Cluster-7025.9	Psi-rd-227699	oleoyl-acyl carrier thioesterase chloroplastic	-6,713005722	2,93652251E-113
Cluster-2958.1693	Psi-rd-264534	phosphoenolpyruvate carboxykinase [ATP]-like	-6,317901413	6,16969850E-106
Cluster-1105.8	Psi-rd-270792	non-specific lipid-transfer	-6,176882323	4,556425906E-95
Cluster-19010.0	Psi-rd-23827	phosphatase 2C 57	5,996086509	1,742809170E-97
Cluster-9704.1	Psi-rd-247198	ferredoxin-dependent glutamate chloroplastic	6,255364303	4,47771684E-128

Cluster	Unigenes	Annotation	log2FoldChange	padj
Cluster-15492.2	Psi-rd-170632	elongation factor G- chloroplastic	6,398807863	4,867357084E-92
Cluster-2958.23966	Psi-rd-248607	hypothetical protein EUGRSUZ_B030602, partial	6,718696682	7,180098767E-98
Cluster-20354.1	Psi-rd-5023	Serine mitochondrial	6,730928924	8,862185036E-94
Cluster-17477.0	Psi-rd-141630	CAAD domains of cyanobacterial aminoacyl-tRNA synthetase	7,361991315	1,796441790E-99
Cluster-2958.464	Psi-rd-240388	ribulose-phosphate 3- chloroplastic	7,436817594	3,814339467E-97
Cluster-18535.0	Psi-rd-159317	cytochrome b6-f complex iron-sulfur	7,623579364	9,73765526E-121
Cluster-5675.7	Psi-rd-122971	NHL repeat-containing 2	7,645966448	5,162263281E-90
Cluster-851.23	Psi-rd-84311	pentatricopeptide repeat-containing At5g65560-like isoform X1	7,862964371	7,00242881E-104
Cluster-2958.10533	Psi-rd-124196	LOV domain-containing	7,908792786	2,312349046E-95
Cluster-2958.25046	Psi-rd-6633	protein CHUP1, chloroplastic	8,242281277	2,94455585E-115
Cluster-20015.1	Psi-rd-58957	serine--glyoxylate aminotransferase	8,342151966	1,73838689E-126
Cluster-11784.0	Psi-rd-106464	CP12 domain	8,458943968	2,058802484E-90
Cluster-2958.15180	Psi-rd-297450	oxygen-evolving enhancer chloroplastic	8,536502542	3,69774548E-101
Cluster-2958.15181	Psi-rd-297449	oxygen-evolving enhancer chloroplastic	8,583730504	4,16507844E-118
Cluster-18935.2	Psi-rd-88284	ferric reduction oxidase chloroplastic	8,641076593	5,866836819E-90
Cluster-20015.3	Psi-rd-58954	serine--glyoxylate aminotransferase	8,746096626	5,87148161E-104
Cluster-18541.0	Psi-rd-255131	ferredoxin--NADP leaf chloroplastic	9,009064603	4,09137853E-124
Cluster-2958.15193	Psi-rd-183517	oxygen-evolving enhancer chloroplastic	9,027904070	4,136168514E-95
Cluster-2958.15186	Psi-rd-4805	oxygen-evolving enhancer chloroplastic	9,383514838	6,371254238E-93
Cluster-2632.26	Psi-rd-224535	ATPase family associated with various cellular activities (AAA)	9,468952834	2,84377032E-144
Cluster-10033.18	Psi-rd-157349	chlorophyll a-b binding chloroplastic	9,579618697	5,38761228E-120
Cluster-19531.6	Psi-rd-209977	chlorophyll a-b binding chloroplastic	9,633119202	7,051777847E-95
Cluster-12155.3	Psi-rd-233351	glyceraldehyde-3-phosphate dehydrogenase chloroplastic	9,634796006	5,76786151E-145
Cluster-20015.2	Psi-rd-288823	serine--glyoxylate aminotransferase	9,641894296	5,938179805E-93
Cluster-7678.21	Psi-rd-229589	chlorophyll a-b binding chloroplastic	9,645027124	3,34842399E-115

Cluster	Unigenes	Annotation	log2FoldChange	padj
Cluster-7678.25	Psi-rd-297112	chlorophyll a b-binding type	9,895648849	9,11299620E-116
Cluster-10033.15	Psi-rd-128444	chlorophyll a-b binding chloroplastic	9,925613634	7,726540623E-98
Cluster-21179.1	Psi-rd-23215	Light regulated protein Lir1	10,05136176	3,62310120E-150
Cluster-2958.29957	Psi-rd-270470	catalase isozyme 1	10,17632250	2,032169384E-99
Cluster-19281.1	Psi-rd-233963	fructose-1,6- chloroplastic	10,27108124	3,363522585E-94
Cluster-15247.0	Psi-rd-190214	NAD dependent epimerase/dehydratase family	10,27479553	2,54424731E-104
Cluster-7678.32	Psi-rd-214041	chlorophyll a-b binding chloroplastic	10,33090314	4,07748360E-134
Cluster-579.4	Psi-rd-15870	chlorophyll a-b binding chloroplastic	10,37285592	2,84377032E-144
Cluster-15858.8	Psi-rd-258635	probable carotenoid cleavage dioxygenase chloroplastic	10,37316596	6,55861826E-146
Cluster-19605.0	Psi-rd-5165	plastocyanin	10,40524653	4,48000671E-163
Cluster-7627.2	Psi-rd-390	fructose-bisphosphate aldolase chloroplastic	10,40954254	3,86864734E-127
Cluster-3996.3	Psi-rd-46782	peroxisomal (S)-2-hydroxy-acid oxidase	10,41513110	2,25414654E-120
Cluster-851.9	Psi-rd-233630	Phosphoribulokinase family	10,72650223	5,79401438E-102
Cluster-14952.3	Psi-rd-175825	Ribulose bisphosphate carboxylase, small chain / Ribulose-1,5-bisphosphate carboxylase small subunit	10,77519520	7,04080579E-100
Cluster-14952.9	Psi-rd-165461	Ribulose bisphosphate carboxylase, small chain / Ribulose-1,5-bisphosphate carboxylase small subunit	10,87454436	4,61356779E-115
Cluster-14952.21	Psi-rd-211411	Ribulose bisphosphate carboxylase, small chain / Ribulose-1,5-bisphosphate carboxylase small subunit	10,99007234	5,62973968E-128
Cluster-9915.10	Psi-rd-16308	carbonic chloroplastic isoform X2	11,10558369	3,361263972E-92
Cluster-14952.40	Psi-rd-165459	Ribulose bisphosphate carboxylase, small chain / Ribulose-1,5-bisphosphate carboxylase small subunit	11,18503520	1,481710169E-94
Cluster-2632.22	Psi-rd-280546	ATPase family associated with various cellular activities (AAA)	11,22237828	1,37842677E-104
Cluster-2632.32	Psi-rd-153474	ATPase family associated with various cellular activities (AAA)	11,43096487	1,54609130E-126
Cluster-3996.4	Psi-rd-107889	peroxisomal (S)-2-hydroxy-acid oxidase	11,49322922	2,71122607E-133
Cluster-2958.33205	Psi-rd-54839	fructose-bisphosphate aldolase chloroplastic	11,50917851	5,57102395E-117

Cluster	Unigenes	Annotation	log2FoldChange	padj
Cluster-2958.20359	Psi-rd-204515	fructose-bisphosphate aldolase chloroplastic	11,63039324	2,95325748E-162
Cluster-9915.24	Psi-rd-174111	carbonic anhydrase	11,65495323	7,04080579E-100
Cluster-14952.35	Psi-rd-139791	Ribulose bisphosphate carboxylase, small chain / Ribulose-1,5-bisphosphate carboxylase small subunit	11,89998858	5,599224207E-99
Cluster-2958.513	Psi-rd-260002	fructose-bisphosphate aldolase chloroplastic	11,90499412	1,63003507E-104
Cluster-2632.37	Psi-rd-280547	ATPase family associated with various cellular activities (AAA)	12,19578715	4,68294114E-108
Cluster-2632.36	Psi-rd-145549	ribulose bisphosphate carboxylase oxygenase chloroplastic	12,21028138	5,80148653E-186
