# Supplementary material for: Transcriptomics analysis of Psidium cattleyanum Sabine (Myrtaceae) unveil potential genes involved in fruit pigmentation
Source: Genet Mol Biol. 2020 Apr 27;43(2):e20190255. doi: 10.1590/1678-4685-GMB-2019-0255 (PMC7199922; doi:10.1590/1678-4685-GMB-2019-0255)
Supplement: Table S4 [file 1415-4757-GMB-43-2-e20190255-s5.pdf]

Supplementary material to: Transcriptomics analysis of *Psidium cattleyanum* Sabine (Myrtaceae) unveil potential genes involved in fruit pigmentation

Table S4 - Top 100 differential gene expression between Unripe vs Ripe fruit in yellow morphotype.

Cluster	Unigenes	Annotation	log2FoldChange	padj
Cluster-10601.2	Psi-yw-166537	hypothetical protein EUGRSUZ_F03213	-11,34112025	1,6537E-48
Cluster-3991.3	Psi-yw-280325	SENESCENCE-ASSOCIATED GENE mitochondrial-like	-10,73551236	1,1267E-52
Cluster-22812.23430	Psi-yw-233535	Pectate lyase superfamily protein	-10,38688344	9,5085E-74
Cluster-3991.0	Psi-yw-149536	toll/interleukin-1 receptor (TIR) domain-containing protein	-9,97663134	4,6374E-50
Cluster-3991.1	Psi-yw-285715	protein SENESCENCE-ASSOCIATED GENE 21, mitochondrial-like	-9,96623036	1,4544E-171
Cluster-3991.7	Psi-yw-280328	Late embryogenesis abundant protein	-9,91575937	1,4763E-68
Cluster-14361.0	Psi-yw-257965	Cys endopeptidase family	-9,88984960	6,3732E-94
Cluster-3991.10	Psi-yw-149533	Late embryogenesis abundant protein	-9,82432728	2,2189E-165
Cluster-3991.4	Psi-yw-108155	SENESCENCE-ASSOCIATED GENE mitochondrial-like	-9,80933619	2,0650E-189
Cluster-20542.2	Psi-yw-46751	1-aminocyclopropane-1-carboxylate synthase 1	-9,76523395	5,2291E-110
Cluster-3991.9	Psi-yw-149535	SENESCENCE-ASSOCIATED GENE mitochondrial-like	-9,70939863	2,8292E-71
Cluster-3935.0	Psi-yw-148831	polyphenol chloroplastic-like	-9,70838392	1,1600E-45
Cluster-3991.13	Psi-yw-285717	Senescence-associated gene	-9,52633091	1,2023E-147
Cluster-3991.5	Psi-yw-96944	SENESCENCE-ASSOCIATED GENE mitochondrial-like	-9,52129581	1,3393E-106

Cluster	Unigenes	Annotation	log2FoldChange	padj
Cluster-22812.1493	Psi-yw-287301	polygalacturonase-like isoform X1	-9,14920509	7,1700E-67
Cluster-22812.27514	Psi-yw-160348	polygalacturonase	-9,11385616	1,9072E-52
Cluster-22812.23428	Psi-yw-160346	Pectate lyase superfamily protein	-9,05686659	2,4607E-63
Cluster-12376.0	Psi-yw-21899	Late embryogenesis abundant protein	-8,54231745	9,1510E-54
Cluster-22812.23432	Psi-yw-112051	Pectate lyase superfamily protein	-8,51092171	9,8554E-69
Cluster-19802.0	Psi-yw-209937	calcium-binding PBP1-like	-8,48411977	5,7779E-45
Cluster-397.12	Psi-yw-285440	germin subfamily 1 member 7	-8,43152524	1,2156E-167
Cluster-3991.8	Psi-yw-280329	Late embryogenesis abundant protein	-8,11852166	1,7456E-58
Cluster-8606.21	Psi-yw-293334	allene oxide cyclase	-7,95470583	8,9502E-74
Cluster-1114.4	Psi-yw-294080	flowering locus T	-7,89796535	3,1105E-57
Cluster-1114.9	Psi-yw-259325	Phosphatidylethanolamine-binding protein	-7,79809080	3,1076E-61
Cluster-397.10	Psi-yw-228698	germin subfamily 1 member 7	-7,70171799	1,2371E-106
Cluster-7272.0	Psi-yw-25611	1-acyl-sn-glycerol-3-phosphate acyltransferase 3	-7,66277561	4,5927E-46
Cluster-22812.674	Psi-yw-113666	L-ascorbate oxidase homolog	-7,54461281	2,6089E-49
Cluster-22812.1035	Psi-yw-150315	Thaumatin family	-7,50335705	4,8945E-62
Cluster-18536.1	Psi-yw-104354	inactive cadmium zinc-transporting ATPase HMA3	-7,40550845	4,8418E-80
Cluster-22812.1040	Psi-yw-211030	Thaumatin family	-7,33639448	2,5154E-53
Cluster-612.20	Psi-yw-225902	pathogenesis-related PR-4	-7,32779260	7,2788E-55
Cluster-22761.0	Psi-yw-87284	hypothetical protein EUGRSUZ_K02199	-7,00363339	2,4677E-48
Cluster-22812.1028	Psi-yw-249838	Thaumatin family	-6,89381640	6,4116E-63
Cluster-22812.20457	Psi-yw-129482	Serine hydrolase (FSH1)	-6,85893745	8,6000E-44
Cluster-6732.0	Psi-yw-292910	anthocyanidin 3-O-glucosyltransferase 2-like	-6,83543021	7,4441E-110
Cluster-22812.1039	Psi-yw-211023	Thaumatin family	-6,80665258	2,5263E-50
Cluster-612.13	Psi-yw-225895	Hevein-like prepro	-6,73402699	1,5648E-68
Cluster-22812.1032	Psi-yw-104836	Thaumatin family	-6,67630157	3,3141E-53
Cluster-22812.1019	Psi-yw-286483	Thaumatin family	-6,62792152	3,0290E-44
Cluster-22812.1041	Psi-yw-249840	Thaumatin family	-6,58732178	1,0258E-44
Cluster-865.5	Psi-yw-237737	cytochrome P450 81F3-like	-6,47942022	4,2209E-46
Cluster-18328.4	Psi-yw-5381	uncharacterized protein LOC104421701	-6,46058105	2,8531E-55

Cluster	Unigenes	Annotation	log2FoldChange	padj
Cluster-14047.1	Psi-yw-33362	uncharacterized protein LOC104437075	-6,31885806	6,1441E-61
Cluster-612.3	Psi-yw-225906	pathogenesis-related PR-4	-6,21347813	4,9960E-55
Cluster-5698.2	Psi-yw-227122	muscle M-line assembly unc-89 isoform X2	-6,19336929	4,4888E-49
Cluster-14674.2	Psi-yw-123462	Complex 1 protein (LYR family)	-6,12934359	1,5253E-48
Cluster-3254.5	Psi-yw-290164	21 kDa	-6,11813074	1,2527E-48
Cluster-7089.17	Psi-yw-284053	Phosphopantetheine attachment site	-6,10038688	9,7796E-99
Cluster-7089.48	Psi-yw-191847	Phosphopantetheine attachment site	-6,07445332	6,9635E-61
Cluster-5707.0	Psi-yw-111717	21 kDa protein	-6,03101736	4,0936E-51
Cluster-22812.6609	Psi-yw-83297	uncharacterized protein LOC104427495	-6,02675921	3,3711E-56
Cluster-7089.9	Psi-yw-284051	Phosphopantetheine attachment site	-5,84304835	5,2736E-97
Cluster-7089.38	Psi-yw-191839	Lecithin retinol acyltransferase	-5,75191208	1,2019E-86
Cluster-22812.27354	Psi-yw-282624	oleoyl-acyl carrier thioesterase chloroplastic	-5,68699961	2,1509E-71
Cluster-3254.10	Psi-yw-147381	21 kDa	-5,66021875	3,5395E-59
Cluster-7089.39	Psi-yw-268345	Phosphopantetheine attachment site	-5,60668214	1,5348E-66
Cluster-7089.36	Psi-yw-284043	acyl carrier chloroplastic	-5,58783007	6,9961E-72
Cluster-11979.0	Psi-yw-122096	Protein of unknown function (DUF3730)	-5,54816500	7,9225E-47
Cluster-3254.20	Psi-yw-266844	21 kDa	-5,50073759	1,4569E-87
Cluster-7791.4	Psi-yw-193537	A Chain The Crystal Structure Of The Complex Between Stearoyl Acyl Carrier Desaturase From Ricinus Communis (Castor Bean) And	-5,46498580	7,5672E-182
Cluster-14674.4	Psi-yw-286168	Complex 1 protein (LYR family)	-5,35104174	1,7846E-42
Cluster-7791.5	Psi-yw-70943	stearoyl-[acyl-carrier-] 9- chloroplastic-like	-5,33342781	5,5176E-126
Cluster-8055.3	Psi-yw-234488	zinc finger ZAT10-like	-5,23127668	9,9198E-47
Cluster-3254.4	Psi-yw-290163	Plant invertase/pectin methylesterase inhibitor	-5,13582561	2,0123E-122
Cluster-8591.2	Psi-yw-221827	Phosphopantetheine attachment site	-5,10693682	5,2296E-82
Cluster-11630.0	Psi-yw-194700	beta-amylase chloroplastic	-5,05392041	3,0951E-46
Cluster-12888.1	Psi-yw-83796	Protein of unknown function (DUF3339)	-4,93848865	1,7965E-48
Cluster-3254.8	Psi-yw-290161	21 kDa -like	-4,93644756	1,6051E-94
Cluster-7011.20	Psi-yw-119510	serine carboxypeptidase-like 2 isoform X1	-4,62545020	6,7186E-46

Cluster	Unigenes	Annotation	log2FoldChange	padj
Cluster-865.8	Psi-yw-66137	isoflavone 2 -hydroxylase	-4,58067178	1,8356E-74
Cluster-22812.11748	Psi-yw-292066	No recognize	-4,56925243	4,5666E-57
Cluster-11630.5	Psi-yw-194699	beta-amylase chloroplastic	-4,27557911	1,9875E-43
Cluster-15099.0	Psi-yw-98041	probable phosphatase 2C 60	-4,26260846	2,7537E-52
Cluster-11189.0	Psi-yw-268415	exonuclease chloroplastic mitochondrial	-4,22190751	4,2598E-63
Cluster-17544.1	Psi-yw-142238	ACT domain	-4,11832671	1,4039E-58
Cluster-22812.30308	Psi-yw-252096	inorganic phosphate transporter 1-4-like	-4,04240878	6,9682E-61
Cluster-15099.2	Psi-yw-98040	probable phosphatase 2C 60	-4,00045680	4,4116E-45
Cluster-16953.1	Psi-yw-240138	non-functional NADPH-dependent codeinone reductase 2	4,20969470	3,4098E-44
Cluster-19414.0	Psi-yw-148480	alpha-1,4 glucan phosphorylase L chloroplastic amyloplastic	4,24560415	9,0066E-50
Cluster-22812.25272	Psi-yw-273104	CATA_IPOBA ame: Full=Catalase	4,26621464	2,0100E-46
Cluster-7033.0	Psi-yw-37423	NHL repeat	4,33537633	2,5557E-47
Cluster-20559.4	Psi-yw-295119	Alpha/beta hydrolase family	4,35131123	1,0684E-47
Cluster-13479.0	Psi-yw-221016	Remorin, C-terminal region / Remorin, N-terminal region	4,61196656	5,7587E-62
Cluster-22812.31720	Psi-yw-280481	auxin-responsive IAA16	4,90037127	9,2059E-45
Cluster-22812.25247	Psi-yw-250446	catalase isozyme 1	5,01370024	1,8800E-49
Cluster-22812.25240	Psi-yw-64792	catalase isozyme 3 isoform X1	5,04028230	6,8756E-51
Cluster-22812.25242	Psi-yw-138192	Catalase / Catalase-related immune-responsive	5,06601817	6,3159E-49
Cluster-22812.25246	Psi-yw-231252	catalase isozyme 1	5,18108057	1,0900E-61
Cluster-22812.25279	Psi-yw-34746	catalase family	5,18129137	6,0824E-53
Cluster-17746.0	Psi-yw-269274	inorganic phosphate transporter 2- chloroplastic	5,24742788	1,2005E-45
Cluster-22812.7114	Psi-yw-24829	2OG-Fe(II) oxygenase superfamily / non-haem dioxygenase in morphine synthesis N-terminal	5,28467995	1,5477E-67
Cluster-22812.25257	Psi-yw-144273	catalase family	5,38161993	3,6388E-45
Cluster-22812.25261	Psi-yw-2542	No recognize	5,43394825	8,4938E-44
Cluster-10585.0	Psi-yw-152630	F-box At4g18380-like	5,51007151	5,5986E-63

Cluster	Unigenes	Annotation	log2FoldChange	padj
Cluster-12723.18	Psi-yw-174780	alcohol dehydrogenase class-3	6,16606715	5,6626E-69
Cluster-12723.17	Psi-yw-56672	alcohol dehydrogenase class-3	6,31086049	1,4356E-56
Cluster-6649.0	Psi-yw-252668	oligopeptide transporter 3	6,58731442	1,2573E-51
Cluster-6649.3	Psi-yw-17282	oligopeptide transporter 3	6,59414809	6,4308E-62
Cluster-22812.29708	Psi-yw-279436	cytochrome P450 82G1	6,80178749	2,9035E-47
