# Supplementary material for: Transcriptomics analysis of Psidium cattleyanum Sabine (Myrtaceae) unveil potential genes involved in fruit pigmentation
Source: Genet Mol Biol. 2020 Apr 27;43(2):e20190255. doi: 10.1590/1678-4685-GMB-2019-0255 (PMC7199922; doi:10.1590/1678-4685-GMB-2019-0255)
Supplement: Table S5 [file 1415-4757-GMB-43-2-e20190255-s6.pdf]

Supplementary material to: Transcriptomics analysis of *Psidium cattleyanum* Sabine (Myrtaceae) unveil potential genes involved in fruit pigmentation

Table S5 - Top 100 differential gene expression between Unripe vs Ripe fruit in red morphotype.

Cluster	Unigenes	Annotation	log2FoldChange	padj
Cluster-2958.21783	Psi-rd-173130	3-hydroxyisobutyryl- hydrolase 1	-10,14234994	8,527959068E-08
Cluster-3398.0	Psi-rd-202325	No recognize	-9,585476912	2,874906270E-07
Cluster-13.0	Psi-rd-97264	aquaporin NIP2-1-like	-9,558033777	4,824761886E-07
Cluster-4787.0	Psi-rd-10331	subtilisin-like protease	-7,898073808	4,352567494E-37
Cluster-2516.1	Psi-rd-188295	Wound-induced protein	-7,079820525	2,665000000E-06
Cluster-20378.0	Psi-rd-110131	non-symbiotic hemoglobin class 1	-7,068608748	3,114500914E-15
Cluster-1036.3	Psi-rd-57651	PLAC8 family	-6,608800502	3,004010314E-39
Cluster-14443.0	Psi-rd-172864	probable purple acid phosphatase 20	-6,211060536	3,776935961E-17
Cluster-7059.1	Psi-rd-140202	vignain	-5,97321784	3,562963529E-35
Cluster-14443.1	Psi-rd-122359	hypothetical protein EUGRSUZ_A01513	-5,912087978	2,148761109E-07
Cluster-2502.1	Psi-rd-19970	Wound-induced protein	-5,866927169	3,386959593E-07
Cluster-15519.1	Psi-rd-176811	probable prolyl 4-hydroxylase 7	-5,791919373	1,023316172E-07
Cluster-2958.28975	Psi-rd-204916	metal transporter Nramp5-like	-5,725212289	2,984468070E-21
Cluster-2443.1	Psi-rd-208312	probable xyloglucan galactosyltransferase GT11	-5,683036258	1,770537850E-12
Cluster-1036.2	Psi-rd-174097	hypothetical protein PanWU01x14_021900	-5,642505865	3,745895183E-32
Cluster-2958.28856	Psi-rd-194822	uncharacterized protein LOC104433009	-5,513327918	2,291613581E-22
Cluster-15519.0	Psi-rd-5858	probable prolyl 4-hydroxylase 7	-5,47551647	3,654108247E-09
Cluster-7059.0	Psi-rd-140204	Cys endopeptidase family	-5,421465463	4,798991260E-10
Cluster-2317.1	Psi-rd-47772	TENA/THI-4/PQQC family	-5,317535408	2,984468070E-21

Cluster	Unigenes	Annotation	log2FoldChange	padj
Cluster-8601.5	Psi-rd-128979	NPH3 family	-5,268113188	2,000000000E-08
Cluster-2119.0	Psi-rd-126209	mannan endo-1,4-beta-mannosidase 5	-5,156127382	7,314132191E-15
Cluster-2958.30662	Psi-rd-138563	GDSL esterase lipase At5g22810	-4,988135957	8,620002489E-13
Cluster-20162.1	Psi-rd-240780	sucrose synthase 5-like	-4,952872242	5,851021050E-14
Cluster-14684.0	Psi-rd-282061	phenylalanine ammonia-lyase	-4,824490709	7,595347121E-10
Cluster-9121.1	Psi-rd-105802	Reverse transcriptase-like / zinc-binding in reverse transcriptase	-4,759536307	6,387175965E-11
Cluster-3434.3	Psi-rd-214717	2OG-Fe(II) oxygenase superfamily	-4,685946503	5,326304506E-09
Cluster-3266.3	Psi-rd-184229	DMR6-LIKE OXYGENASE 2	-4,665178924	3,297049314E-29
Cluster-16055.0	Psi-rd-206702	Myb/SANT-like DNA-binding domain	-4,576785836	1,634505689E-10
Cluster-2436.2	Psi-rd-92334	phospholipase A1-IIgamma-like	-4,542399182	7,693942128E-28
Cluster-2958.21538	Psi-rd-165087	leucoanthocyanidin dioxygenase	-4,495127051	6,989712974E-13
Cluster-2436.3	Psi-rd-285217	phospholipase A1-IIgamma	-4,491927502	1,690738248E-16
Cluster-18665.1	Psi-rd-186772	WAT1-related At5g47470	-4,467025201	1,409952605E-37
Cluster-3547.1	Psi-rd-99459	beta-glucosidase 24-like	-4,383174567	6,091339300E-14
Cluster-19469.4	Psi-rd-146418	Alpha/beta hydrolase family	-4,342209451	1,054225507E-06
Cluster-19778.3	Psi-rd-91602	serine carboxypeptidase-like 7	-4,340787684	5,934325117E-10
Cluster-14274.19	Psi-rd-194111	aspartate chloroplastic	-4,334301413	8,189560217E-09
Cluster-21567.0	Psi-rd-191357	Plant protein of unknown function (DUF946)	-4,330909519	8,339571141E-10
Cluster-16338.0	Psi-rd-124686	Ring finger domain	-4,302502366	3,311606534E-07
Cluster-16055.2	Psi-rd-255568	Myb/SANT-like DNA-binding domain	-4,293104559	8,492878519E-12
Cluster-2356.4	Psi-rd-55805	Late embryogenesis abundant	-4,22889227	7,514425170E-11
Cluster-18594.2	Psi-rd-138302	plant cysteine oxidase 2	-4,216024015	2,160259529E-06
Cluster-3388.0	Psi-rd-65618	UDP-glucose:flavonoid 3-O-glycosyltransferase: UFGT	-4,207943936	4,590927302E-18
Cluster-5902.0	Psi-rd-144290	probable metal-nicotianamine transporter YSL7	-4,176809701	7,806353230E-14
Cluster-2958.27142	Psi-rd-36836	uncharacterized protein LOC104449274	-4,155891373	4,128259676E-08
Cluster-3938.1	Psi-rd-61250	NADH-ubiquinone reductase complex 1 MLRQ subunit	-4,150252652	1,368302818E-06
Cluster-20162.0	Psi-rd-58325	sucrose synthase 5-like	-4,14700074	1,985044634E-07
Cluster-19778.0	Psi-rd-212629	serine carboxypeptidase-like 1	-4,127091591	2,660000000E-07

Cluster	Unigenes	Annotation	log2FoldChange	padj
Cluster-2958.14648	Psi-rd-217759	catalase isozyme 3	-4,095653762	3,806821425E-21
Cluster-8376.0	Psi-rd-110195	DUF1223 domain-containing	-4,047309423	2,507656581E-09
Cluster-2069.15	Psi-rd-256261	chalcone isomerase	-4,014697194	2,820800766E-06
Cluster-2431.0	Psi-rd-144987	phytoene	-4,012176117	1,793037294E-07
Cluster-14684.1	Psi-rd-75320	phenylalanine ammonia-lyase	-4,010920074	6,217414153E-07
Cluster-21699.0	Psi-rd-286490	Auxin canalisation / Plant pleckstrin homology-like region	4,014131142	1,930701289E-10
Cluster-2958.8270	Psi-rd-295751	Protein of unknown function (DUF568)	4,017312618	1,775145435E-20
Cluster-17146.0	Psi-rd-265837	photosystem I reaction center subunit IV chloroplastic	4,030611794	1,583853943E-06
Cluster-8073.0	Psi-rd-60505	Bromodomain domain-containing	4,036740246	4,089184689E-08
Cluster-6350.12	Psi-rd-234427	uncharacterized protein LOC102662543 isoform X2	4,055649754	1,512438443E-29
Cluster-2958.30092	Psi-rd-200613	No recognize	4,084594436	1,724134508E-05
Cluster-2958.25213	Psi-rd-291030	No recognize	4,102844558	1,432000000E-05
Cluster-2958.17821	Psi-rd-272315	No recognize	4,131701752	9,449000000E-06
Cluster-2958.1196	Psi-rd-292222	Leucine Rich Repeat	4,139766724	5,304270003E-07
Cluster-6350.7	Psi-rd-234425	Dormancy/auxin associated protein	4,152156748	1,085298497E-42
Cluster-2958.1163	Psi-rd-128927	Senescence regulator	4,205021586	7,452606067E-09
Cluster-2958.19452	Psi-rd-240468	transcriptional corepressor LEUNIG isoform X2	4,211925909	1,109712004E-18
Cluster-2958.8129	Psi-rd-295752	Protein of unknown function (DUF568)	4,26392215	3,804883794E-10
Cluster-2179.0	Psi-rd-297933	bZIP transcription factor 2-like	4,274100932	9,188280004E-23
Cluster-2958.33612	Psi-rd-87915	No recognize	4,28469638	1,507550784E-05
Cluster-7751.0	Psi-rd-59393	Phosphate-induced protein 1 conserved region	4,284719423	1,421096147E-13
Cluster-6436.1	Psi-rd-108583	F-box At4g18380-like	4,302105729	2,296762248E-09
Cluster-2958.28540	Psi-rd-280391	photosystem I reaction center subunit chloroplastic	4,324412001	1,366291212E-07
Cluster-10069.0	Psi-rd-109154	7337009KCW73803.1 hypothetical protein EUGRSUZ_E02406	4,389581321	5,733726021E-06
Cluster-2958.14283	Psi-rd-22431	No recognize	4,427023248	4,170186930E-10
Cluster-3599.5	Psi-rd-279794	dehydrin DHN1	4,430038314	2,562225991E-06
Cluster-2958.15532	Psi-rd-240999	dehydration-responsive element-binding 3-like	4,465909173	8,898824067E-09
Cluster-2958.1172	Psi-rd-246529	Senescence regulator	4,544022079	2,009471207E-06

Cluster	Unigenes	Annotation	log2FoldChange	padj
Cluster-2958.21543	Psi-rd-266391	leucine-rich repeat receptor	4,595619298	6,137481653E-11
Cluster-8411.5	Psi-rd-173400	Zinc-binding dehydrogenase / Alcohol dehydrogenase GroES-like domain	4,596908166	3,404648037E-07
Cluster-2958.33734	Psi-rd-281988	No recognize	4,600245998	2,026763181E-14
Cluster-17940.1	Psi-rd-106675	ethylene-responsive transcription factor ERF034	4,643045769	8,878224593E-18
Cluster-2958.24505	Psi-rd-186758	hypothetical protein MIMGU_mgv1a024143mg	4,653135595	3,720710408E-33
Cluster-3761.2	Psi-rd-289273	cytochrome P450 71A1-like	4,689992021	6,777218870E-08
Cluster-4515.1	Psi-rd-187103	PHD-finger / Ring finger domain	4,725146533	4,606163033E-06
Cluster-2958.4671	Psi-rd-145609	No recognize	4,912324802	2,042499128E-08
Cluster-10914.0	Psi-rd-150486	Pectate lyase	4,915853021	1,139960590E-08
Cluster-3599.10	Psi-rd-279791	dehydrin DHN1	4,921483462	5,976252146E-06
Cluster-2958.29015	Psi-rd-26002	TRAF-like family protein	4,957438661	1,184404535E-07
Cluster-2958.25200	Psi-rd-186759	Pollen allergen / Rare lipoprotein A (RlpA)-like double-psi beta-barrel	5,121348276	1,911461459E-12
Cluster-3599.4	Psi-rd-279788	No recognize	5,19092308	5,620274111E-08
Cluster-4314.0	Psi-rd-159291	Leucoanthocyanidin dioxygenase	5,237001028	9,512361249E-07
Cluster-2958.18589	Psi-rd-240470	hypothetical protein DM860_002287	5,317623258	1,145098513E-10
Cluster-21520.0	Psi-rd-282743	hypothetical protein EUGRSUZ_H00312	5,396331771	6,827187857E-07
Cluster-2958.32692	Psi-rd-281984	Glycine rich protein family	5,617795744	6,247406661E-12
Cluster-4009.21	Psi-rd-290558	beta-bisabolene synthase-like	5,625727922	1,139960590E-08
Cluster-6070.5	Psi-rd-98404	benzyl alcohol O-benzoyltransferase	5,836680096	4,176832786E-11
Cluster-2958.13955	Psi-rd-228836	21 kDa -like	5,926712486	5,190601491E-10
Cluster-12511.0	Psi-rd-58872	hypothetical protein EUGRSUZ_C03164	6,184743297	1,331135472E-07
Cluster-4096.0	Psi-rd-30808	GH3 auxin-responsive promoter	6,55417288	9,982311475E-40
Cluster-1875.1	Psi-rd-136528	Nodulin-like	6,788531713	1,867298921E-06
Cluster-3637.0	Psi-rd-257301	Nodulin-like	7,77885677	6,530553536E-13
Cluster-6070.12	Psi-rd-98406	benzyl alcohol O-benzoyltransferase	9,054506591	1,054225507E-06
