# Supplementary material for: Transcriptomics analysis of Psidium cattleyanum Sabine (Myrtaceae) unveil potential genes involved in fruit pigmentation
Source: Genet Mol Biol. 2020 Apr 27;43(2):e20190255. doi: 10.1590/1678-4685-GMB-2019-0255 (PMC7199922; doi:10.1590/1678-4685-GMB-2019-0255)
Supplement: Table S6 [file 1415-4757-GMB-43-2-e20190255-s7.pdf]

Supplementary material to: Transcriptomics analysis of *Psidium cattleyanum* Sabine (Myrtaceae) unveil potential genes involved in fruit pigmentation

Table S6 - Species information used to perform UDP-glucose: flavonoid 3-O-glucosyltransferase (UFGT) phylogenetic analysis.

Species	**Annotation**	**Acronym**
Citrus sinensis	orange1.1g009851m.g Org_Csinensis peptide: orange1.1g009851m (1 of 2) PTHR11926//PTHR11926:SF362 - GLUCOSYL/GLUCURONOSYL TRANSFERASES // SUBFAMILY NOT NAMED (PAC:18092892)	Csi_1g009851
Citrus sinensis	orange1.1g010093m.g Org_Csinensis peptide: orange1.1g010093m (1 of 2) PTHR11926//PTHR11926:SF190 - GLUCOSYL/GLUCURONOSYL TRANSFERASES // SUBFAMILY NOT NAMED (PAC:18100224)	Csi_1g010093
Citrus sinensis	orange1.1g009851m.g Org_Csinensis peptide: orange1.1g010617m (1 of 2) PTHR11926//PTHR11926:SF362 - GLUCOSYL/GLUCURONOSYL TRANSFERASES // SUBFAMILY NOT NAMED (PAC:18092893)	Csi_1g010617
Citrus sinensis	orange1.1g010684m.g Org_Csinensis peptide: orange1.1g010684m (1 of 7) 2.4.1.85 - Cyanohydrin beta-glucosyltransferase / Uridine diphosphoglucose:aldehyde cyanohydrin beta-glucosyltransferase (PAC:18106939)	Csi_1g010684

Species	Annotation	Acronym
Citrus sinensis	orange1.1g010684m.g Org_Csinensis peptide: orange1.1g010775m (1 of 7) 2.4.1.85 - Cyanohydrin beta-glucosyltransferase / Uridine diphosphoglucose:aldehyde cyanohydrin beta-glucosyltransferase (PAC:18106940)	Csi_1g010775
Citrus sinensis	orange1.1g010825m.g Org_Csinensis peptide: orange1.1g010825m (1 of 5) PTHR11926//PTHR11926:SF235 - GLUCOSYL/GLUCURONOSYL TRANSFERASES // SUBFAMILY NOT NAMED (PAC:18106811)	Csi_1g010825
Citrus sinensis	orange1.1g010940m.g Org_Csinensis peptide: orange1.1g010940m (1 of 13) K13496 - UDP-glucosyl transferase 73C [EC:2.4.1.-] Glc - brassinosteroid (UGT73C) (PAC:18097882)	Csi_1g010940
Citrus sinensis	orange1.1g010988m.g Org_Csinensis peptide: orange1.1g010988m (1 of 13) K13496 - UDP-glucosyl transferase 73C [EC:2.4.1.-] Glc - brassinosteroid (UGT73C) (PAC:18097877)	Csi_1g010988
Citrus sinensis	orange1.1g011099m.g Org_Csinensis peptide: orange1.1g011099m (1 of 3) K12356 - coniferyl-alcohol glucosyltransferase (UGT72E) (PAC:18125099)	Csi_1g011099
Citrus sinensis	orange1.1g011106m.g Org_Csinensis peptide: orange1.1g011106m (1 of 8) PTHR11926//PTHR11926:SF392 - GLUCOSYL/GLUCURONOSYL TRANSFERASES // SUBFAMILY NOT NAMED (PAC:18128774)	Csi_1g011106
Citrus sinensis	orange1.1g011142m.g Org_Csinensis peptide: orange1.1g011142m (1 of 14) 2.4.1.203 - Trans-zeatin O-beta-D-glucosyltransferase / Zeatin O-glucosyltransferase (PAC:18123465)	Csi_1g011142

Species	**Annotation**	**Acronym**
Citrus sinensis	orange1.1g011339m.g Org_Csinensis peptide: orange1.1g011339m (1 of 17) PTHR11926:SF248 - UDP-GLYCOSYLTRANSFERASE 73C7 (PAC:18097885)	Csi_1g011339
Citrus sinensis	orange1.1g011381m.g Org_Csinensis peptide: orange1.1g011381m (1 of 7) 2.4.1.218 - Hydroquinone glucosyltransferase / Hydroquinone:O-glucosyltransferase (PAC:18119660)	Csi_1g011381
Citrus sinensis	orange1.1g011396m.g Org_Csinensis peptide: orange1.1g011396m (1 of 7) 2.4.1.218 - Hydroquinone glucosyltransferase / Hydroquinone:O-glucosyltransferase (PAC:18119667)	Csi_1g011396
Citrus sinensis	orange1.1g011490m.g Org_Csinensis peptide: orange1.1g011490m (1 of 13) K13496 - UDP-glucosyl transferase 73C [EC:2.4.1.-] Glc - brassinosteroid (UGT73C) (PAC:18117775)	Csi_1g011490
Citrus sinensis	orange1.1g011531m.g Org_Csinensis peptide: orange1.1g011531m (1 of 13) 2.4.1.323 - 7-deoxyloganetic acid glucosyltransferase (PAC:18123981)	Csi_1g011531
Citrus sinensis	orange1.1g011608m.g Org_Csinensis peptide: orange1.1g011608m (1 of 3) K12356 - coniferyl-alcohol glucosyltransferase (UGT72E) (PAC:18124560)	Csi_1g011608
Citrus sinensis	orange1.1g011687m.g Org_Csinensis peptide: orange1.1g011687m (1 of 1) PTHR11926:SF149 - UDP-GLYCOSYLTRANSFERASE 82A1 (PAC:18107007)	Csi_1g011687

Species	**Annotation**	**Acronym**
Citrus sinensis	orange1.1g011724m.g Org_Csinensis peptide: orange1.1g011724m (1 of 2) PTHR11926//PTHR11926:SF383 - GLUCOSYL/GLUCURONOSYL TRANSFERASES // SUBFAMILY NOT NAMED (PAC:18106332)	Csi_1g011724
Citrus sinensis	orange1.1g011765m.g Org_Csinensis peptide: orange1.1g011765m (1 of 1) PTHR11926//PTHR11926:SF367 - GLUCOSYL/GLUCURONOSYL TRANSFERASES // SUBFAMILY NOT NAMED (PAC:18139297)	Csi_1g011765
Citrus sinensis	orange1.1g011789m.g Org_Csinensis peptide: orange1.1g011789m (1 of 2) PTHR11926//PTHR11926:SF383 - GLUCOSYL/GLUCURONOSYL TRANSFERASES // SUBFAMILY NOT NAMED (PAC:18104317)	Csi_1g011789
Citrus sinensis	orange1.1g011792m.g Org_Csinensis peptide: orange1.1g011792m (1 of 13) 2.4.1.323 - 7-deoxyloganetic acid glucosyltransferase (PAC:18127949)	Csi_1g011792
Citrus sinensis	orange1.1g011832m.g Org_Csinensis peptide: orange1.1g011832m (1 of 13) 2.4.1.323 - 7-deoxyloganetic acid glucosyltransferase (PAC:18138365)	Csi_1g011832
Citrus sinensis	orange1.1g011848m.g Org_Csinensis peptide: orange1.1g011848m (1 of 13) 2.4.1.323 - 7-deoxyloganetic acid glucosyltransferase (PAC:18137986)	Csi_1g011848
Citrus sinensis	orange1.1g012063m.g Org_Csinensis peptide: orange1.1g012063m (1 of 7) 2.4.1.218 - Hydroquinone glucosyltransferase / Hydroquinone:O-glucosyltransferase (PAC:18136774)	Csi_1g012063

Species	**Annotation**	**Acronym**
Citrus sinensis	orange1.1g012096m.g Org_Csinensis peptide: orange1.1g012096m (1 of 3) PTHR11926:SF176 - UDP-GLYCOSYLTRANSFERASE 87A2 (PAC:18111309)	Csi_1g012096
Citrus sinensis	orange1.1g012151m.g Org_Csinensis peptide: orange1.1g012151m (1 of 10) 2.4.1.118 - Cytokinin 7-beta-glucosyltransferase / Uridine diphosphoglucose-zeatin 7-glucosyltransferase (PAC:18134625)	Csi_1g012151
Citrus sinensis	orange1.1g012194m.g Org_Csinensis peptide: orange1.1g012194m (1 of 7) K13691 - pathogen-inducible salicylic acid glucosyltransferase [EC:2.4.1.-] Glc b1-2 SA (SGT1) (PAC:18119636)	Csi_1g012194
Citrus sinensis	orange1.1g012212m.g Org_Csinensis peptide: orange1.1g012212m (1 of 7) 2.4.1.85 - Cyanohydrin beta-glucosyltransferase / Uridine diphosphoglucose:aldehyde cyanohydrin beta-glucosyltransferase (PAC:18134914)	Csi_1g012212
Citrus sinensis	orange1.1g010684m.g Org_Csinensis peptide: orange1.1g012217m (1 of 7) 2.4.1.85 - Cyanohydrin beta-glucosyltransferase / Uridine diphosphoglucose:aldehyde cyanohydrin beta-glucosyltransferase (PAC:18106941)	Csi_1g012217
Citrus sinensis	orange1.1g012277m.g Org_Csinensis peptide: orange1.1g012277m (1 of 3) PTHR11926:SF213 - UDP-GLYCOSYLTRANSFERASE 74B1 (PAC:18119613)	Csi_1g012277
Citrus sinensis	orange1.1g009851m.g Org_Csinensis peptide: orange1.1g012314m (1 of 2) PTHR11926//PTHR11926:SF362 - GLUCOSYL/GLUCURONOSYL TRANSFERASES // SUBFAMILY NOT NAMED (PAC:18092894)	Csi_1g012314

Species	Annotation	Acronym
Citrus sinensis	orange1.1g010684m.g Org_Csinensis peptide: orange1.1g012342m (1 of 7) 2.4.1.85 - Cyanohydrin beta-glucosyltransferase / Uridine diphosphoglucose:aldehyde cyanohydrin beta-glucosyltransferase (PAC:18106942)	Csi_1g012342
Citrus sinensis	orange1.1g012277m.g Org_Csinensis peptide: orange1.1g012412m (1 of 3) PTHR11926:SF213 - UDP-GLYCOSYLTRANSFERASE 74B1 (PAC:18119614)	Csi_1g012412
Citrus sinensis	orange1.1g012474m.g Org_Csinensis peptide: orange1.1g012474m (1 of 10) 2.4.1.118 - Cytokinin 7-beta-glucosyltransferase / Uridine diphosphoglucose-zeatin 7-glucosyltransferase (PAC:18138037)	Csi_1g012474
Citrus sinensis	orange1.1g012513m.g Org_Csinensis peptide: orange1.1g012513m (1 of 7) 2.4.1.218 - Hydroquinone glucosyltransferase / Hydroquinone:O-glucosyltransferase (PAC:18119658)	Csi_1g012513
Citrus sinensis	orange1.1g012543m.g Org_Csinensis peptide: orange1.1g012543m (1 of 10) 2.4.1.118 - Cytokinin 7-beta-glucosyltransferase / Uridine diphosphoglucose-zeatin 7-glucosyltransferase (PAC:18101251)	Csi_1g012543
Citrus sinensis	orange1.1g012563m.g Org_Csinensis peptide: orange1.1g012563m (1 of 10) 2.4.1.118 - Cytokinin 7-beta-glucosyltransferase / Uridine diphosphoglucose-zeatin 7-glucosyltransferase (PAC:18097343)	Csi_1g012563
Citrus sinensis	orange1.1g011848m.g Org_Csinensis peptide: orange1.1g012587m (1 of 13) 2.4.1.323 - 7-deoxyloganetic acid glucosyltransferase (PAC:18137987)	Csi_1g012587

Species	**Annotation**	**Acronym**
Citrus sinensis	orange1.1g012594m.g Org_Csinensis peptide: orange1.1g012594m (1 of 14) 2.4.1.203 - Trans-zeatin O-beta-D-glucosyltransferase / Zeatin O-glucosyltransferase (PAC:18134968)	Csi_1g012594
Citrus sinensis	orange1.1g012613m.g Org_Csinensis peptide: orange1.1g012613m (1 of 10) 2.4.1.118 - Cytokinin 7-beta-glucosyltransferase / Uridine diphosphoglucose-zeatin 7-glucosyltransferase (PAC:18139806)	Csi_1g012613
Citrus sinensis	orange1.1g012645m.g Org_Csinensis peptide: orange1.1g012645m (1 of 1) K11820 - N-hydroxythioamide S-beta-glucosyltransferase (UGT74B1) (PAC:18132321)	Csi_1g012645
Citrus sinensis	orange1.1g012652m.g Org_Csinensis peptide: orange1.1g012652m (1 of 15) 2.4.1.324 - 7-deoxyloganetin glucosyltransferase / UGT85A24 (PAC:18099682)	Csi_1g012652
Citrus sinensis	orange1.1g012474m.g Org_Csinensis peptide: orange1.1g012678m (1 of 10) 2.4.1.118 - Cytokinin 7-beta-glucosyltransferase / Uridine diphosphoglucose-zeatin 7-glucosyltransferase (PAC:18138038)	Csi_1g012678
Citrus sinensis	orange1.1g012735m.g Org_Csinensis peptide: orange1.1g012735m (1 of 10) 2.4.1.118 - Cytokinin 7-beta-glucosyltransferase / Uridine diphosphoglucose-zeatin 7-glucosyltransferase (PAC:18138376)	Csi_1g012735
Citrus sinensis	orange1.1g012744m.g Org_Csinensis peptide: orange1.1g012744m (1 of 15) 2.4.1.324 - 7-deoxyloganetin glucosyltransferase / UGT85A24 (PAC:18135105)	Csi_1g012744

Species	**Annotation**	**Acronym**
Citrus sinensis	orange1.1g012893m.g Org_Csinensis peptide: orange1.1g012893m (1 of 2) 2.4.1.115//2.4.1.91 - Anthocyanidin 3-O-glucosyltransferase / Uridine diphosphoglucose-anthocyanidin 3-O-glucosyltransferase // Flavonol 3-O-glucosyltransferase / UDP-glucose flavonol 3-O-glucosyltransferase (PAC:18104187)	Csi_1g012893_RefSeq
Citrus sinensis	orange1.1g013342m.g Org_Csinensis peptide: orange1.1g013342m (1 of 6) PTHR11926:SF102 - GLUCOSYLTRANSFERASE-LIKE PROTEIN-RELATED (PAC:18111621)	Csi_1g013342
Citrus sinensis	orange1.1g013836m.g Org_Csinensis peptide: orange1.1g013836m (1 of 10) 2.4.1.118 - Cytokinin 7-beta-glucosyltransferase / Uridine diphosphoglucose-zeatin 7-glucosyltransferase (PAC:18120941)	Csi_1g013836
Citrus sinensis	orange1.1g013878m.g Org_Csinensis peptide: orange1.1g013878m (1 of 2) 2.4.1.115//2.4.1.91 - Anthocyanidin 3-O-glucosyltransferase / Uridine diphosphoglucose-anthocyanidin 3-O-glucosyltransferase // Flavonol 3-O-glucosyltransferase / UDP-glucose flavonol 3-O-glucosyltransferase (PAC:18120695)	Csi_1g013878
Citrus sinensis	orange1.1g014232m.g Org_Csinensis peptide: orange1.1g014232m (1 of 6) PTHR11926:SF102 - GLUCOSYLTRANSFERASE-LIKE PROTEIN-RELATED (PAC:18111557)	Csi_1g014232
Citrus sinensis	orange1.1g016062m.g Org_Csinensis peptide: orange1.1g016062m (1 of 6) PTHR11926:SF102 - GLUCOSYLTRANSFERASE-LIKE PROTEIN-RELATED (PAC:18111232)	Csi_1g016062
Citrus sinensis	orange1.1g017266m.g Org_Csinensis peptide: orange1.1g017266m (1 of 14) 2.4.1.203 - Trans-zeatin O-beta-D-glucosyltransferase / Zeatin O-glucosyltransferase (PAC:18134918)	Csi_1g017266

Species	Annotation	Acronym
Citrus sinensis	orange1.1g018483m.g Org_Csinensis peptide: orange1.1g018483m (1 of 6) PTHR11926:SF102 - GLUCOSYLTRANSFERASE-LIKE PROTEIN-RELATED (PAC:18096270)	Csi_1g018483
Citrus sinensis	orange1.1g019791m.g Org_Csinensis peptide: orange1.1g019791m (1 of 3) 2.4.1.185 - Flavanone 7-O-beta-glucosyltransferase (PAC:18122766)	Csi_1g019791
Citrus sinensis	orange1.1g012613m.g Org_Csinensis peptide: orange1.1g020179m (1 of 10) 2.4.1.118 - Cytokinin 7-beta-glucosyltransferase / Uridine diphosphoglucose-zeatin 7-glucosyltransferase (PAC:18139807)	Csi_1g020179
Citrus sinensis	orange1.1g035856m.g Org_Csinensis peptide: orange1.1g035856m (1 of 4) 2.4.1.91 - Flavonol 3-O-glucosyltransferase / UDP-glucose flavonol 3-O-glucosyltransferase (PAC:18126288)	Csi_1g035856
Citrus sinensis	orange1.1g036436m.g Org_Csinensis peptide: orange1.1g036436m (1 of 5) PTHR11926//PTHR11926:SF118 - GLUCOSYL/GLUCURONOSYL TRANSFERASES // SUBFAMILY NOT NAMED (PAC:18125471)	Csi_1g036436
Citrus sinensis	orange1.1g036740m.g Org_Csinensis peptide: orange1.1g036740m (1 of 3) 2.4.1.271 - Crocetin glucosyltransferase / UGT75L6 (PAC:18092975)	Csi_1g036740
Citrus sinensis	orange1.1g036871m.g Org_Csinensis peptide: orange1.1g036871m (1 of 7) 2.4.1.128 - Scopoletin glucosyltransferase (PAC:18116069)	Csi_1g036871

Species	**Annotation**	**Acronym**
Citrus sinensis	orange1.1g037374m.g Org_Csinensis peptide: orange1.1g037374m (1 of 13) 2.4.1.323 - 7-deoxyloganetic acid glucosyltransferase (PAC:18135475)	Csi_1g037374
Citrus sinensis	orange1.1g037640m.g Org_Csinensis peptide: orange1.1g037640m (1 of 14) 2.4.1.203 - Trans-zeatin O-beta-D-glucosyltransferase / Zeatin O-glucosyltransferase (PAC:18100579)	Csi_1g037640
Citrus sinensis	orange1.1g037999m.g Org_Csinensis peptide: orange1.1g037999m (1 of 13) 2.4.1.323 - 7-deoxyloganetic acid glucosyltransferase (PAC:18103869)	Csi_1g037999
Citrus sinensis	orange1.1g039701m.g Org_Csinensis peptide: orange1.1g039701m (1 of 13) K13496 - UDP-glucosyl transferase 73C [EC:2.4.1.-] Glc - brassinosteroid (UGT73C) (PAC:18117781)	Csi_1g039701
Citrus sinensis	orange1.1g040486m.g Org_Csinensis peptide: orange1.1g040486m (1 of 10) 2.4.1.118 - Cytokinin 7-beta-glucosyltransferase / Uridine diphosphoglucose-zeatin 7-glucosyltransferase (PAC:18101153)	Csi_1g040486
Citrus sinensis	orange1.1g041902m.g Org_Csinensis peptide: orange1.1g041902m (1 of 3) 2.4.1.271 - Crocetin glucosyltransferase / UGT75L6 (PAC:18119752)	Csi_1g041902
Citrus sinensis	orange1.1g042731m.g Org_Csinensis peptide: orange1.1g042731m (1 of 8) PTHR11926//PTHR11926:SF392 - GLUCOSYL/GLUCURONOSYL TRANSFERASES // SUBFAMILY NOT NAMED (PAC:18128793)	Csi_1g042731

Species	**Annotation**	**Acronym**
Citrus sinensis	orange1.1g042970m.g Org_Csinensis peptide: orange1.1g042970m (1 of 13) K13496 - UDP-glucosyl transferase 73C [EC:2.4.1.-] Glc - brassinosteroid (UGT73C) (PAC:18116243)	Csi_1g042970
Citrus sinensis	orange1.1g042987m.g Org_Csinensis peptide: orange1.1g042987m (1 of 5) PTHR11926//PTHR11926:SF118 - GLUCOSYL/GLUCURONOSYL TRANSFERASES // SUBFAMILY NOT NAMED (PAC:18125409)	Csi_1g042987
Citrus sinensis	orange1.1g043168m.g Org_Csinensis peptide: orange1.1g043168m (1 of 8) PTHR11926//PTHR11926:SF392 - GLUCOSYL/GLUCURONOSYL TRANSFERASES // SUBFAMILY NOT NAMED (PAC:18128738)	Csi_1g043168
Citrus sinensis	orange1.1g043290m.g Org_Csinensis peptide: orange1.1g043290m (1 of 2) PTHR11926:SF374 - UDP-GLYCOSYLTRANSFERASE 71C4 (PAC:18113667)	Csi_1g043290
Citrus sinensis	orange1.1g043304m.g Org_Csinensis peptide: orange1.1g043304m (1 of 8) PTHR11926//PTHR11926:SF392 - GLUCOSYL/GLUCURONOSYL TRANSFERASES // SUBFAMILY NOT NAMED (PAC:18128812)	Csi_1g043304
Citrus sinensis	orange1.1g043859m.g Org_Csinensis peptide: orange1.1g043859m (1 of 4) PTHR11926:SF223 - UDP-GLYCOSYLTRANSFERASE 72E1-RELATED (PAC:18125131)	Csi_1g043859
Citrus sinensis	orange1.1g044012m.g Org_Csinensis peptide: orange1.1g044012m (1 of 1) PTHR11926//PTHR11926:SF384 - GLUCOSYL/GLUCURONOSYL TRANSFERASES // SUBFAMILY NOT NAMED (PAC:18120605)	Csi_1g044012

Species	**Annotation**	**Acronym**
Citrus sinensis	orange1.1g044031m.g Org_Csinensis peptide: orange1.1g044031m (1 of 1) PTHR11926:SF343 - UDP-GLYCOSYLTRANSFERASE 88A1 (PAC:18103620)	Csi_1g044031
Citrus sinensis	orange1.1g044266m.g Org_Csinensis peptide: orange1.1g044266m (1 of 14) 2.4.1.203 - Trans-zeatin O-beta-D-glucosyltransferase / Zeatin O-glucosyltransferase (PAC:18135158)	Csi_1g044266
Citrus sinensis	orange1.1g044936m.g Org_Csinensis peptide: orange1.1g044936m (1 of 13) 2.4.1.323 - 7-deoxyloganetic acid glucosyltransferase (PAC:18123980)	Csi_1g044936
Citrus sinensis	orange1.1g045029m.g Org_Csinensis peptide: orange1.1g045029m (1 of 3) PTHR11926:SF242 - UDP-GLYCOSYLTRANSFERASE 71B2-RELATED (PAC:18106781)	Csi_1g045029
Citrus sinensis	orange1.1g045267m.g Org_Csinensis peptide: orange1.1g045267m (1 of 7) 2.4.1.218 - Hydroquinone glucosyltransferase / Hydroquinone:O-glucosyltransferase (PAC:18135959)	Csi_1g045267
Citrus sinensis	orange1.1g045281m.g Org_Csinensis peptide: orange1.1g045281m (1 of 5) PTHR11926//PTHR11926:SF118 - GLUCOSYL/GLUCURONOSYL TRANSFERASES // SUBFAMILY NOT NAMED (PAC:18125392)	Csi_1g045281
Citrus sinensis	orange1.1g045570m.g Org_Csinensis peptide: orange1.1g045570m (1 of 2) PTHR11926:SF374 - UDP-GLYCOSYLTRANSFERASE 71C4 (PAC:18094977)	Csi_1g045570

Species	Annotation	Acronym
Citrus sinensis	orange1.1g045998m.g Org_Csinensis peptide: orange1.1g045998m (1 of 2) K13692 - UDP-glucose:(indol-3-yl)acetate beta-D-glucosyltransferase [EC:2.4.1.121] Glc b1- IAA (IAGLU) (PAC:18093903)	Csi_1g045998
Citrus sinensis	orange1.1g046167m.g Org_Csinensis peptide: orange1.1g046167m (1 of 6) PTHR11926:SF102 - GLUCOSYLTRANSFERASE-LIKE PROTEIN-RELATED (PAC:18111193)	Csi_1g046167
Citrus sinensis	orange1.1g046326m.g Org_Csinensis peptide: orange1.1g046326m (1 of 2) PTHR11926:SF381 - UDP-GLYCOSYLTRANSFERASE 72B1 (PAC:18119818)	Csi_1g046326
Citrus sinensis	orange1.1g046339m.g Org_Csinensis peptide: orange1.1g046339m (1 of 2) K13692 - UDP-glucose:(indol-3-yl)acetate beta-D-glucosyltransferase [EC:2.4.1.121] Glc b1- IAA (IAGLU) (PAC:18100115)	Csi_1g046339
Citrus sinensis	orange1.1g046605m.g Org_Csinensis peptide: orange1.1g046605m (1 of 7) 2.4.1.128 - Scopoletin glucosyltransferase (PAC:18100131)	Csi_1g046605
Citrus sinensis	orange1.1g047445m.g Org_Csinensis peptide: orange1.1g047445m (1 of 2) PTHR11926:SF381 - UDP-GLYCOSYLTRANSFERASE 72B1 (PAC:18091827)	Csi_1g047445
Citrus sinensis	orange1.1g047540m.g Org_Csinensis peptide: orange1.1g047540m (1 of 5) PTHR11926//PTHR11926:SF235 - GLUCOSYL/GLUCURONOSYL TRANSFERASES // SUBFAMILY NOT NAMED (PAC:18107011)	Csi_1g047540

Species	**Annotation**	**Acronym**
Citrus sinensis	orange1.1g047662m.g Org_Csinensis peptide: orange1.1g047662m (1 of 13) 2.4.1.323 - 7-deoxyloganetic acid glucosyltransferase (PAC:18092923)	Csi_1g047662
Citrus sinensis	orange1.1g047833m.g Org_Csinensis peptide: orange1.1g047833m (1 of 8) PTHR11926//PTHR11926:SF392 - GLUCOSYL/GLUCURONOSYL TRANSFERASES // SUBFAMILY NOT NAMED (PAC:18128719)	Csi_1g047833
Citrus sinensis	orange1.1g048393m.g Org_Csinensis peptide: orange1.1g048393m (1 of 7) K13691 - pathogen-inducible salicylic acid glucosyltransferase [EC:2.4.1.-] Glc b1-2 SA (SGT1) (PAC:18129764)	Csi_1g048393
Citrus sinensis	orange1.1g048435m.g Org_Csinensis peptide: orange1.1g048435m (1 of 2) 2.4.1.210 - Limonoid glucosyltransferase / Limonoid UDP-glucosyltransferase (PAC:18139677)	Csi_1g048435
Eucalyptus grandis	Eucgr.A00303 Org_Egrandis peptide: Eucgr.A00303.1.p (1 of 21) 2.4.1.115//2.4.1.91 - Anthocyanidin 3-O-glucosyltransferase / Uridine diphosphoglucose-anthocyanidin 3-O-glucosyltransferase // Flavonol 3-O-glucosyltransferase / UDP-glucose flavonol 3-O-glucosyltransferase (PAC:32048022)	Egr_A00303
Eucalyptus grandis	Eucgr.A00304 Org_Egrandis peptide: Eucgr.A00304.1.p (1 of 21) 2.4.1.115//2.4.1.91 - Anthocyanidin 3-O-glucosyltransferase / Uridine diphosphoglucose-anthocyanidin 3-O-glucosyltransferase // Flavonol 3-O-glucosyltransferase / UDP-glucose flavonol 3-O-glucosyltransferase (PAC:32049090)	Egr_A00304

Species	**Annotation**	**Acronym**
Eucalyptus grandis	Eucgr.A00306 Org_Egrandis peptide: Eucgr.A00306.1.p (1 of 21) 2.4.1.115//2.4.1.91 - Anthocyanidin 3-O-glucosyltransferase / Uridine diphosphoglucose-anthocyanidin 3-O-glucosyltransferase // Flavonol 3-O-glucosyltransferase / UDP-glucose flavonol 3-O-glucosyltransferase (PAC:32047415)	Egr_A00306
Eucalyptus grandis	Eucgr.A00424 Org_Egrandis peptide: Eucgr.A00424.1.p (1 of 14) 2.4.1.118 - Cytokinin 7-beta-glucosyltransferase / Uridine diphosphoglucose-zeatin 7-glucosyltransferase (PAC:32046323)	Egr_A00424
Eucalyptus grandis	Eucgr.A01691 Org_Egrandis peptide: Eucgr.A01691.1.p (1 of 2) PTHR11926//PTHR11926:SF383 - GLUCOSYL/GLUCURONOSYL TRANSFERASES // SUBFAMILY NOT NAMED (PAC:32045971)	Egr_A01691
Eucalyptus grandis	Eucgr.A02964 Org_Egrandis peptide: Eucgr.A02964.1.p (1 of 21) 2.4.1.115//2.4.1.91 - Anthocyanidin 3-O-glucosyltransferase / Uridine diphosphoglucose-anthocyanidin 3-O-glucosyltransferase // Flavonol 3-O-glucosyltransferase / UDP-glucose flavonol 3-O-glucosyltransferase (PAC:32046748)	Egr_A02964_RefSeq
Eucalyptus grandis	Eucgr.B01109 Org_Egrandis peptide: Eucgr.B01109.1.p (1 of 62) 2.4.1.324 - 7-deoxyloganetin glucosyltransferase / UGT85A24 (PAC:32058347)	Egr_B01109
Eucalyptus grandis	Eucgr.B02291 Org_Egrandis peptide: Eucgr.B02291.1.p (1 of 9) PTHR11926:SF102 - GLUCOSYLTRANSFERASE-LIKE PROTEIN-RELATED (PAC:32058404)	Egr_B02291
Eucalyptus grandis	Eucgr.B02292 Org_Egrandis peptide: Eucgr.B02292.1.p (1 of 9) PTHR11926:SF102 - GLUCOSYLTRANSFERASE-LIKE PROTEIN-RELATED (PAC:32059077)	Egr_B02292

Species	**Annotation**	**Acronym**
Eucalyptus grandis	Eucgr.B02537 Org_Egrandis peptide: Eucgr.B02537.1.p (1 of 2) PTHR11926//PTHR11926:SF383 - GLUCOSYL/GLUCURONOSYL TRANSFERASES // SUBFAMILY NOT NAMED (PAC:32061803)	Egr_B02537
Eucalyptus grandis	Eucgr.B02563 Org_Egrandis peptide: Eucgr.B02563.1.p (1 of 1) 2.4.1.195 - N-hydroxythioamide S-beta-glucosyltransferase / Uridine diphosphoglucose-thiohydroximate glucosyltransferase (PAC:32061118)	Egr_B02563
Eucalyptus grandis	Eucgr.B02744 Org_Egrandis peptide: Eucgr.B02744.1.p (1 of 5) 2.4.1.323 - 7-deoxyloganetic acid glucosyltransferase (PAC:32046515)	Egr_B02744
Eucalyptus grandis	Eucgr.C00191 Org_Egrandis peptide: Eucgr.C00191.1.p (1 of 24) PTHR11926:SF292 - UDP-GLYCOSYLTRANSFERASE 78D1-RELATED (PAC:32039399)	Egr_C00191
Eucalyptus grandis	Eucgr.C00831 Org_Egrandis peptide: Eucgr.C00831.1.p (1 of 21) 2.4.1.115//2.4.1.91 - Anthocyanidin 3-O-glucosyltransferase / Uridine diphosphoglucose-anthocyanidin 3-O-glucosyltransferase // Flavonol 3-O-glucosyltransferase / UDP-glucose flavonol 3-O-glucosyltransferase (PAC:32037962)	Egr_C00831
Eucalyptus grandis	Eucgr.C00833 Org_Egrandis peptide: Eucgr.C00833.1.p (1 of 21) 2.4.1.115//2.4.1.91 - Anthocyanidin 3-O-glucosyltransferase / Uridine diphosphoglucose-anthocyanidin 3-O-glucosyltransferase // Flavonol 3-O-glucosyltransferase / UDP-glucose flavonol 3-O-glucosyltransferase (PAC:32039955)	Egr_C00833
Eucalyptus grandis	Eucgr.C01016 Org_Egrandis peptide: Eucgr.C01016.1.p (1 of 21) 2.4.1.115//2.4.1.91 - Anthocyanidin 3-O-glucosyltransferase / Uridine diphosphoglucose-anthocyanidin 3-O-glucosyltransferase // Flavonol 3-O-glucosyltransferase / UDP-glucose flavonol 3-O-glucosyltransferase (PAC:32037450)	Egr_C01016

Species	Annotation	Acronym
Eucalyptus grandis	Eucgr.C01017 Org_Egrandis peptide: Eucgr.C01017.1.p (1 of 21) 2.4.1.115//2.4.1.91 - Anthocyanidin 3-O-glucosyltransferase / Uridine diphosphoglucose-anthocyanidin 3-O-glucosyltransferase // Flavonol 3-O-glucosyltransferase / UDP-glucose flavonol 3-O-glucosyltransferase (PAC:32038714)	Egr_C01017
Eucalyptus grandis	Eucgr.C01020 Org_Egrandis peptide: Eucgr.C01020.1.p (1 of 21) 2.4.1.115//2.4.1.91 - Anthocyanidin 3-O-glucosyltransferase / Uridine diphosphoglucose-anthocyanidin 3-O-glucosyltransferase // Flavonol 3-O-glucosyltransferase / UDP-glucose flavonol 3-O-glucosyltransferase (PAC:32038085)	Egr_C01020
Eucalyptus grandis	Eucgr.C01021 Org_Egrandis peptide: Eucgr.C01021.1.p (1 of 21) 2.4.1.115//2.4.1.91 - Anthocyanidin 3-O-glucosyltransferase / Uridine diphosphoglucose-anthocyanidin 3-O-glucosyltransferase // Flavonol 3-O-glucosyltransferase / UDP-glucose flavonol 3-O-glucosyltransferase (PAC:32039857)	Egr_C01021
Eucalyptus grandis	Eucgr.C01022 Org_Egrandis peptide: Eucgr.C01022.1.p (1 of 21) 2.4.1.115//2.4.1.91 - Anthocyanidin 3-O-glucosyltransferase / Uridine diphosphoglucose-anthocyanidin 3-O-glucosyltransferase // Flavonol 3-O-glucosyltransferase / UDP-glucose flavonol 3-O-glucosyltransferase (PAC:32036199)	Egr_C01022
Eucalyptus grandis	Eucgr.C01063 Org_Egrandis peptide: Eucgr.C01063.1.p (1 of 24) PTHR11926:SF292 - UDP-GLYCOSYLTRANSFERASE 78D1-RELATED (PAC:32037757)	Egr_C01063
Eucalyptus grandis	Eucgr.D00702 Org_Egrandis peptide: Eucgr.D00702.1.p (1 of 21) 2.4.1.115//2.4.1.91 - Anthocyanidin 3-O-glucosyltransferase / Uridine diphosphoglucose-anthocyanidin 3-O-glucosyltransferase // Flavonol 3-O-glucosyltransferase / UDP-glucose flavonol 3-O-glucosyltransferase (PAC:32052173)	Egr_D00702
Eucalyptus grandis	Eucgr.E01562 Org_Egrandis peptide: Eucgr.E01562.1.p (1 of 62) 2.4.1.324 - 7-deoxyloganetin glucosyltransferase / UGT85A24 (PAC:32028265)	Egr_E01562

Species	**Annotation**	**Acronym**
Eucalyptus grandis	Eucgr.E01567 Org_Egrandis peptide: Eucgr.E01567.1.p (1 of 62) 2.4.1.324 - 7-deoxyloganetin glucosyltransferase / UGT85A24 (PAC:32029619)	Egr_E01567
Eucalyptus grandis	Eucgr.E02345 Org_Egrandis peptide: Eucgr.E02345.1.p (1 of 21) 2.4.1.115//2.4.1.91 - Anthocyanidin 3-O-glucosyltransferase / Uridine diphosphoglucose-anthocyanidin 3-O-glucosyltransferase // Flavonol 3-O-glucosyltransferase / UDP-glucose flavonol 3-O-glucosyltransferase (PAC:32029392)	Egr_E02345
Eucalyptus grandis	Eucgr.E02398 Org_Egrandis peptide: Eucgr.E02398.1.p (1 of 21) 2.4.1.115//2.4.1.91 - Anthocyanidin 3-O-glucosyltransferase / Uridine diphosphoglucose-anthocyanidin 3-O-glucosyltransferase // Flavonol 3-O-glucosyltransferase / UDP-glucose flavonol 3-O-glucosyltransferase (PAC:32030736)	Egr_E02398
Eucalyptus grandis	Eucgr.E02973 Org_Egrandis peptide: Eucgr.E02973.1.p (1 of 21) 2.4.1.115//2.4.1.91 - Anthocyanidin 3-O-glucosyltransferase / Uridine diphosphoglucose-anthocyanidin 3-O-glucosyltransferase // Flavonol 3-O-glucosyltransferase / UDP-glucose flavonol 3-O-glucosyltransferase (PAC:32028544)	Egr_E02973
Eucalyptus grandis	Eucgr.E02976 Org_Egrandis peptide: Eucgr.E02976.1.p (1 of 21) 2.4.1.115//2.4.1.91 - Anthocyanidin 3-O-glucosyltransferase / Uridine diphosphoglucose-anthocyanidin 3-O-glucosyltransferase // Flavonol 3-O-glucosyltransferase / UDP-glucose flavonol 3-O-glucosyltransferase (PAC:32031594)	Egr_E02976
Eucalyptus grandis	Eucgr.E02977 Org_Egrandis peptide: Eucgr.E02977.1.p (1 of 21) 2.4.1.115//2.4.1.91 - Anthocyanidin 3-O-glucosyltransferase / Uridine diphosphoglucose-anthocyanidin 3-O-glucosyltransferase // Flavonol 3-O-glucosyltransferase / UDP-glucose flavonol 3-O-glucosyltransferase (PAC:32031118)	Egr_E02977
Eucalyptus grandis	Eucgr.E02978 Org_Egrandis peptide: Eucgr.E02978.1.p (1 of 21) 2.4.1.115//2.4.1.91 - Anthocyanidin 3-O-glucosyltransferase / Uridine diphosphoglucose-anthocyanidin 3-O-glucosyltransferase // Flavonol 3-O-glucosyltransferase / UDP-glucose flavonol 3-O-glucosyltransferase (PAC:32028317)	Egr_E02978

Species	**Annotation**	**Acronym**
Eucalyptus grandis	Eucgr.F00337 Org_Egrandis peptide: Eucgr.F00337.1.p (1 of 62) 2.4.1.324 - 7-deoxyloganetin glucosyltransferase / UGT85A24 (PAC:32054796)	Egr_F00337
Eucalyptus grandis	Eucgr.F00338 Org_Egrandis peptide: Eucgr.F00338.1.p (1 of 62) 2.4.1.324 - 7-deoxyloganetin glucosyltransferase / UGT85A24 (PAC:32055537)	Egr_F00338
Eucalyptus grandis	Eucgr.F00339 Org_Egrandis peptide: Eucgr.F00339.1.p (1 of 62) 2.4.1.324 - 7-deoxyloganetin glucosyltransferase / UGT85A24 (PAC:32057110)	Egr_F00339
Eucalyptus grandis	Eucgr.F00340 Org_Egrandis peptide: Eucgr.F00340.1.p (1 of 62) 2.4.1.324 - 7-deoxyloganetin glucosyltransferase / UGT85A24 (PAC:32055963)	Egr_F00340
Eucalyptus grandis	Eucgr.F00343 Org_Egrandis peptide: Eucgr.F00343.1.p (1 of 62) 2.4.1.324 - 7-deoxyloganetin glucosyltransferase / UGT85A24 (PAC:32052708)	Egr_F00343
Eucalyptus grandis	Eucgr.F00346 Org_Egrandis peptide: Eucgr.F00346.1.p (1 of 62) 2.4.1.324 - 7-deoxyloganetin glucosyltransferase / UGT85A24 (PAC:32054255)	Egr_F00346
Eucalyptus grandis	Eucgr.F00348 Org_Egrandis peptide: Eucgr.F00348.1.p (1 of 62) 2.4.1.324 - 7-deoxyloganetin glucosyltransferase / UGT85A24 (PAC:32055855)	Egr_F00348

Species	**Annotation**	**Acronym**
Eucalyptus grandis	Eucgr.F00353 Org_Egrandis peptide: Eucgr.F00353.1.p (1 of 62) 2.4.1.324 - 7-deoxyloganetin glucosyltransferase / UGT85A24 (PAC:32054426)	Egr_F00353
Eucalyptus grandis	Eucgr.F03591 Org_Egrandis peptide: Eucgr.F03591.1.p (1 of 13) PTHR11926//PTHR11926:SF235 - GLUCOSYL/GLUCURONOSYL TRANSFERASES // SUBFAMILY NOT NAMED (PAC:32052989)	Egr_F03591
Eucalyptus grandis	Eucgr.F03592 Org_Egrandis peptide: Eucgr.F03592.1.p (1 of 13) PTHR11926//PTHR11926:SF235 - GLUCOSYL/GLUCURONOSYL TRANSFERASES // SUBFAMILY NOT NAMED (PAC:32057231)	Egr_F03592
Eucalyptus grandis	Eucgr.F03595 Org_Egrandis peptide: Eucgr.F03595.1.p (1 of 13) PTHR11926//PTHR11926:SF235 - GLUCOSYL/GLUCURONOSYL TRANSFERASES // SUBFAMILY NOT NAMED (PAC:32054927)	Egr_F03595
Eucalyptus grandis	Eucgr.F03596 Org_Egrandis peptide: Eucgr.F03596.1.p (1 of 13) PTHR11926//PTHR11926:SF235 - GLUCOSYL/GLUCURONOSYL TRANSFERASES // SUBFAMILY NOT NAMED (PAC:32053686)	Egr_F03596
Eucalyptus grandis	Eucgr.F03599 Org_Egrandis peptide: Eucgr.F03599.1.p (1 of 13) PTHR11926//PTHR11926:SF235 - GLUCOSYL/GLUCURONOSYL TRANSFERASES // SUBFAMILY NOT NAMED (PAC:32053344)	Egr_F03599
Eucalyptus grandis	Eucgr.F03601 Org_Egrandis peptide: Eucgr.F03601.1.p (1 of 13) PTHR11926//PTHR11926:SF235 - GLUCOSYL/GLUCURONOSYL TRANSFERASES // SUBFAMILY NOT NAMED (PAC:32055390)	Egr_F03601

Species	**Annotation**	**Acronym**
Eucalyptus grandis	Eucgr.G01342 Org_Egrandis peptide: Eucgr.G01342.1.p (1 of 3) PTHR11926:SF167 - UDP-GLYCOSYLTRANSFERASE 84A1-RELATED (PAC:32071954)	Egr_G01342
Eucalyptus grandis	Eucgr.G01348 Org_Egrandis peptide: Eucgr.G01348.1.p (1 of 1) K13692 - UDP-glucose:(indol-3-yl)acetate beta-D-glucosyltransferase [EC:2.4.1.121] Glc b1- IAA (IAGLU) (PAC:32071497)	Egr_G01348
Eucalyptus grandis	Eucgr.G02215 Org_Egrandis peptide: Eucgr.G02215.1.p (1 of 13) PTHR11926//PTHR11926:SF235 - GLUCOSYL/GLUCURONOSYL TRANSFERASES // SUBFAMILY NOT NAMED (PAC:32073445)	Egr_G02215
Eucalyptus grandis	Eucgr.G02216 Org_Egrandis peptide: Eucgr.G02216.1.p (1 of 13) PTHR11926//PTHR11926:SF235 - GLUCOSYL/GLUCURONOSYL TRANSFERASES // SUBFAMILY NOT NAMED (PAC:32072641)	Egr_G02216
Eucalyptus grandis	Eucgr.G02217 Org_Egrandis peptide: Eucgr.G02217.1.p (1 of 9) 2.4.1.85 - Cyanohydrin beta-glucosyltransferase / Uridine diphosphoglucose:aldehyde cyanohydrin beta-glucosyltransferase (PAC:32071731)	Egr_G02217
Eucalyptus grandis	Eucgr.G02220 Org_Egrandis peptide: Eucgr.G02220.1.p (1 of 13) PTHR11926//PTHR11926:SF235 - GLUCOSYL/GLUCURONOSYL TRANSFERASES // SUBFAMILY NOT NAMED (PAC:32071526)	Egr_G02220
Eucalyptus grandis	Eucgr.G03006 Org_Egrandis peptide: Eucgr.G03006.1.p (1 of 14) 2.4.1.118 - Cytokinin 7-beta-glucosyltransferase / Uridine diphosphoglucose-zeatin 7-glucosyltransferase (PAC:32070786)	Egr_G03006

Species	Annotation	Acronym
Eucalyptus grandis	Eucgr.G03009 Org_Egrandis peptide: Eucgr.G03009.1.p (1 of 14) 2.4.1.118 - Cytokinin 7-beta-glucosyltransferase / Uridine diphosphoglucose-zeatin 7-glucosyltransferase (PAC:32072030)	Egr_G03009
Eucalyptus grandis	Eucgr.G03010 Org_Egrandis peptide: Eucgr.G03010.1.p (1 of 14) 2.4.1.118 - Cytokinin 7-beta-glucosyltransferase / Uridine diphosphoglucose-zeatin 7-glucosyltransferase (PAC:32070976)	Egr_G03010
Eucalyptus grandis	Eucgr.G03013 Org_Egrandis peptide: Eucgr.G03013.1.p (1 of 14) 2.4.1.118 - Cytokinin 7-beta-glucosyltransferase / Uridine diphosphoglucose-zeatin 7-glucosyltransferase (PAC:32071791)	Egr_G03013
Eucalyptus grandis	Eucgr.G03014 Org_Egrandis peptide: Eucgr.G03014.1.p (1 of 14) 2.4.1.118 - Cytokinin 7-beta-glucosyltransferase / Uridine diphosphoglucose-zeatin 7-glucosyltransferase (PAC:32072829)	Egr_G03014
Eucalyptus grandis	Eucgr.G03386 Org_Egrandis peptide: Eucgr.G03386.1.p (1 of 9) 2.4.1.85 - Cyanohydrin beta-glucosyltransferase / Uridine diphosphoglucose:aldehyde cyanohydrin beta-glucosyltransferase (PAC:32072897)	Egr_G03386
Eucalyptus grandis	Eucgr.H04754 Org_Egrandis peptide: Eucgr.H04754.1.p (1 of 8) PTHR11926//PTHR11926:SF269 - GLUCOSYL/GLUCURONOSYL TRANSFERASES // SUBFAMILY NOT NAMED (PAC:32043090)	Egr_H04754
Eucalyptus grandis	Eucgr.H04756 Org_Egrandis peptide: Eucgr.H04756.1.p (1 of 9) 2.4.1.85 - Cyanohydrin beta-glucosyltransferase / Uridine diphosphoglucose:aldehyde cyanohydrin beta-glucosyltransferase (PAC:32042512)	Egr_H04756

Species	**Annotation**	**Acronym**
Eucalyptus grandis	Eucgr.H04759 Org_Egrandis peptide: Eucgr.H04759.1.p (1 of 8) PTHR11926//PTHR11926:SF269 - GLUCOSYL/GLUCURONOSYL TRANSFERASES // SUBFAMILY NOT NAMED (PAC:32043359)	Egr_H04759
Eucalyptus grandis	Eucgr.H04760 Org_Egrandis peptide: Eucgr.H04760.1.p (1 of 8) PTHR11926//PTHR11926:SF269 - GLUCOSYL/GLUCURONOSYL TRANSFERASES // SUBFAMILY NOT NAMED (PAC:32044350)	Egr_H04760
Eucalyptus grandis	Eucgr.H04762 Org_Egrandis peptide: Eucgr.H04762.1.p (1 of 8) PTHR11926//PTHR11926:SF269 - GLUCOSYL/GLUCURONOSYL TRANSFERASES // SUBFAMILY NOT NAMED (PAC:32042166)	Egr_H04762
Eucalyptus grandis	Eucgr.H04764 Org_Egrandis peptide: Eucgr.H04764.1.p (1 of 8) PTHR11926//PTHR11926:SF269 - GLUCOSYL/GLUCURONOSYL TRANSFERASES // SUBFAMILY NOT NAMED (PAC:32045076)	Egr_H04764
Eucalyptus grandis	Eucgr.H04766 Org_Egrandis peptide: Eucgr.H04766.1.p (1 of 9) 2.4.1.85 - Cyanohydrin beta-glucosyltransferase / Uridine diphosphoglucose:aldehyde cyanohydrin beta-glucosyltransferase (PAC:32042964)	Egr_H04766
Eucalyptus grandis	Eucgr.I00512 Org_Egrandis peptide: Eucgr.I00512.1.p (1 of 9) 2.4.1.85 - Cyanohydrin beta-glucosyltransferase / Uridine diphosphoglucose:aldehyde cyanohydrin beta-glucosyltransferase (PAC:32063333)	Egr_I00512
Eucalyptus grandis	Eucgr.I01103 Org_Egrandis peptide: Eucgr.I01103.1.p (1 of 3) PTHR11926:SF167 - UDP-GLYCOSYLTRANSFERASE 84A1-RELATED (PAC:32065273)	Egr_I01103

Species	Annotation	Acronym
Eucalyptus grandis	Eucgr.I02607 Org_Egrandis peptide: Eucgr.I02607.1.p (1 of 21) 2.4.1.115//2.4.1.91 - Anthocyanidin 3-O-glucosyltransferase / Uridine diphosphoglucose-anthocyanidin 3-O-glucosyltransferase // Flavonol 3-O-glucosyltransferase / UDP-glucose flavonol 3-O-glucosyltransferase (PAC:32065650)	Egr_I02607
Eucalyptus grandis	Eucgr.I02647 Org_Egrandis peptide: Eucgr.I02647.1.p (1 of 62) 2.4.1.324 - 7-deoxyloganetin glucosyltransferase / UGT85A24 (PAC:32064495)	Egr_I02647
Eucalyptus grandis	Eucgr.I02655 Org_Egrandis peptide: Eucgr.I02655.1.p (1 of 62) 2.4.1.324 - 7-deoxyloganetin glucosyltransferase / UGT85A24 (PAC:32065375)	Egr_I02655
Eucalyptus grandis	Eucgr.I02656 Org_Egrandis peptide: Eucgr.I02656.1.p (1 of 62) 2.4.1.324 - 7-deoxyloganetin glucosyltransferase / UGT85A24 (PAC:32062953)	Egr_I02656
Eucalyptus grandis	Eucgr.J00970 Org_Egrandis peptide: Eucgr.J00970.1.p (1 of 14) 2.4.1.118 - Cytokinin 7-beta-glucosyltransferase / Uridine diphosphoglucose-zeatin 7-glucosyltransferase (PAC:32033830)	Egr_J00970
Eucalyptus grandis	Eucgr.J00972 Org_Egrandis peptide: Eucgr.J00972.1.p (1 of 14) 2.4.1.118 - Cytokinin 7-beta-glucosyltransferase / Uridine diphosphoglucose-zeatin 7-glucosyltransferase (PAC:32034479)	Egr_J00972
Eucalyptus grandis	Eucgr.J00977 Org_Egrandis peptide: Eucgr.J00977.1.p (1 of 5) 2.4.1.323 - 7-deoxyloganetic acid glucosyltransferase (PAC:32035536)	Egr_J00977

Species	**Annotation**	**Acronym**
Eucalyptus grandis	Eucgr.J01276 Org_Egrandis peptide: Eucgr.J01276.1.p (1 of 1) PTHR11926:SF143 - UDP-GLYCOSYLTRANSFERASE 85A2-RELATED (PAC:32032457)	Egr_J01276
Eucalyptus grandis	Eucgr.J01962 Org_Egrandis peptide: Eucgr.J01962.1.p (1 of 9) PTHR11926:SF102 - GLUCOSYLTRANSFERASE-LIKE PROTEIN-RELATED (PAC:32033392)	Egr_J01962
Eucalyptus grandis	Eucgr.J01963 Org_Egrandis peptide: Eucgr.J01963.1.p (1 of 9) PTHR11926:SF102 - GLUCOSYLTRANSFERASE-LIKE PROTEIN-RELATED (PAC:32034453)	Egr_J01963
Eucalyptus grandis	Eucgr.J01967 Org_Egrandis peptide: Eucgr.J01967.1.p (1 of 9) PTHR11926:SF102 - GLUCOSYLTRANSFERASE-LIKE PROTEIN-RELATED (PAC:32034815)	Egr_J01967
Eucalyptus grandis	Eucgr.J01968 Org_Egrandis peptide: Eucgr.J01968.1.p (1 of 9) PTHR11926:SF102 - GLUCOSYLTRANSFERASE-LIKE PROTEIN-RELATED (PAC:32035627)	Egr_J01968
Eucalyptus grandis	Eucgr.J01971 Org_Egrandis peptide: Eucgr.J01971.1.p (1 of 9) PTHR11926:SF102 - GLUCOSYLTRANSFERASE-LIKE PROTEIN-RELATED (PAC:32033353)	Egr_J01971
Eucalyptus grandis	Eucgr.J01973 Org_Egrandis peptide: Eucgr.J01973.1.p (1 of 9) PTHR11926:SF102 - GLUCOSYLTRANSFERASE-LIKE PROTEIN-RELATED (PAC:32033439)	Egr_J01973

Species	**Annotation**	**Acronym**
Eucalyptus grandis	Eucgr.K02352 Org_Egrandis peptide: Eucgr.K02352.1.p (1 of 3) PTHR11926//PTHR11926:SF331 - GLUCOSYL/GLUCURONOSYL TRANSFERASES // SUBFAMILY NOT NAMED (PAC:32068580)	Egr_K02352
Eucalyptus grandis	Eucgr.K02353 Org_Egrandis peptide: Eucgr.K02353.1.p (1 of 3) PTHR11926//PTHR11926:SF331 - GLUCOSYL/GLUCURONOSYL TRANSFERASES // SUBFAMILY NOT NAMED (PAC:32068551)	Egr_K02353
Eucalyptus grandis	Eucgr.K02355 Org_Egrandis peptide: Eucgr.K02355.1.p (1 of 3) PTHR11926//PTHR11926:SF331 - GLUCOSYL/GLUCURONOSYL TRANSFERASES // SUBFAMILY NOT NAMED (PAC:32065904)	Egr_K02355
Eucalyptus grandis	Eucgr.L00502 Org_Egrandis peptide: Eucgr.L00502.1.p (1 of 62) 2.4.1.324 - 7-deoxyloganetin glucosyltransferase / UGT85A24 (PAC:32063418)	Egr_L00502
Eucalyptus grandis	Eucgr.L00503 Org_Egrandis peptide: Eucgr.L00503.1.p (1 of 62) 2.4.1.324 - 7-deoxyloganetin glucosyltransferase / UGT85A24 (PAC:32062728)	Egr_L00503
Eucalyptus grandis	Eucgr.L00508 Org_Egrandis peptide: Eucgr.L00508.1.p (1 of 62) 2.4.1.324 - 7-deoxyloganetin glucosyltransferase / UGT85A24 (PAC:32064897)	Egr_L00508
Eucalyptus grandis	Eucgr.L00511 Org_Egrandis peptide: Eucgr.L00511.1.p (1 of 62) 2.4.1.324 - 7-deoxyloganetin glucosyltransferase / UGT85A24 (PAC:32064438)	Egr_L00511

Species	Annotation	Acronym
Eucalyptus grandis	Eucgr.L00512 Org_Egrandis peptide: Eucgr.L00512.1.p (1 of 62) 2.4.1.324 - 7-deoxyloganetin glucosyltransferase / UGT85A24 (PAC:32063740)	Egr_L00512
Eucalyptus grandis	Eucgr.L00599 Org_Egrandis peptide: Eucgr.L00599.1.p (1 of 62) 2.4.1.324 - 7-deoxyloganetin glucosyltransferase / UGT85A24 (PAC:32027692)	Egr_L00599
Eucalyptus grandis	Eucgr.L00991 Org_Egrandis peptide: Eucgr.L00991.1.p (1 of 62) 2.4.1.324 - 7-deoxyloganetin glucosyltransferase / UGT85A24 (PAC:32027596)	Egr_L00991
Eucalyptus grandis	Eucgr.L00994 Org_Egrandis peptide: Eucgr.L00994.1.p (1 of 62) 2.4.1.324 - 7-deoxyloganetin glucosyltransferase / UGT85A24 (PAC:32027597)	Egr_L00994
Eucalyptus grandis	Eucgr.L02520 Org_Egrandis peptide: Eucgr.L02520.1.p (1 of 21) 2.4.1.115//2.4.1.91 - Anthocyanidin 3-O-glucosyltransferase / Uridine diphosphoglucose-anthocyanidin 3-O-glucosyltransferase // Flavonol 3-O-glucosyltransferase / UDP-glucose flavonol 3-O-glucosyltransferase (PAC:32027635)	Egr_L02520
Eucalyptus grandis	Eucgr.L02741 Org_Egrandis peptide: Eucgr.L02741.1.p (1 of 21) 2.4.1.115//2.4.1.91 - Anthocyanidin 3-O-glucosyltransferase / Uridine diphosphoglucose-anthocyanidin 3-O-glucosyltransferase // Flavonol 3-O-glucosyltransferase / UDP-glucose flavonol 3-O-glucosyltransferase (PAC:32069862)	Egr_L02741
Psidium cattleyanum	7-deoxyloganetin glucosyltransferase	Psi_yw_107685

Species	Annotation	Acronym
Psidium cattleyanum	7-deoxyloganetin glucosyltransferase	Psi_yw_152475
Psidium cattleyanum	7-deoxyloganetin glucosyltransferase	Psi_yw_152477
Psidium cattleyanum	7-deoxyloganetin glucosyltransferase	Psi_yw_183028
Psidium cattleyanum	7-deoxyloganetin glucosyltransferase	Psi_yw_25904
Psidium cattleyanum	7-deoxyloganetin glucosyltransferase	Psi_yw_286996
Psidium cattleyanum	7-deoxyloganetin glucosyltransferase	Psi_yw_294524
Psidium cattleyanum	7-deoxyloganetin glucosyltransferase	Psi_yw_294529
Psidium cattleyanum	7-deoxyloganetin glucosyltransferase	Psi_yw_294530
Psidium cattleyanum	anthocyanidin 3-O-glucoside 2 -O-glucosyltransferase-like	Psi_yw_197
Psidium cattleyanum	anthocyanidin 3-O-glucosyltransferase 2	Psi_yw_136430
Psidium cattleyanum	anthocyanidin 3-O-glucosyltransferase 2	Psi_yw_142523
Psidium cattleyanum	anthocyanidin 3-O-glucosyltransferase 2	Psi_yw_178580
Psidium cattleyanum	anthocyanidin 3-O-glucosyltransferase 2	Psi_yw_209774
Psidium cattleyanum	anthocyanidin 3-O-glucosyltransferase 2	Psi_yw_251645
Psidium cattleyanum	anthocyanidin 3-O-glucosyltransferase 2	Psi_yw_267791
Psidium cattleyanum	anthocyanidin 3-O-glucosyltransferase 2	Psi_yw_283965
Psidium cattleyanum	anthocyanidin 3-O-glucosyltransferase 2	Psi_yw_64969
Psidium cattleyanum	anthocyanidin 3-O-glucosyltransferase 2-like	Psi_yw_283104
Psidium cattleyanum	anthocyanidin 3-O-glucosyltransferase 2-like	Psi_yw_292910

Species	Annotation	Acronym
Psidium cattleyanum	anthocyanidin 3-O-glucosyltransferase 7	Psi_yw_246102
Psidium cattleyanum	Anthocyanidin 5,3-O-glucosyltransferase	Psi_yw_174927
Psidium cattleyanum	beta-D-glucosyl crocetin beta-1,6-glucosyltransferase-like	Psi_yw_77699
Psidium cattleyanum	bifunctional UDP-glucose 4-epimerase and UDP-xylose 4-epimerase 1	Psi_yw_276096
Psidium cattleyanum	cinnamate beta-D-glucosyltransferase-like	Psi_yw_236488
Psidium cattleyanum	dolichyl-diphosphooligosaccharide-- glycosyltransferase 48 kDa subunit-like	Psi_yw_17394
Psidium cattleyanum	dolichyl-diphosphooligosaccharide-- glycosyltransferase subunit 2	Psi_yw_99688
Psidium cattleyanum	flavonoid 3-O-glucosyltransferase	Psi_yw_172184
Psidium cattleyanum	hydroquinone glucosyltransferase	Psi_yw_146651
Psidium cattleyanum	hydroquinone glucosyltransferase-like	Psi_yw_38988
Psidium cattleyanum	probable glycosyltransferase At3g42180	Psi_yw_129025
Psidium cattleyanum	7-deoxyloganetic acid glucosyltransferase-like	Psi_yw_193512
Psidium cattleyanum	UDP-glycosyltransferase 84B1-like	Psi_rd_104181
Psidium cattleyanum	UDP-glycosyltransferase 91C1-like	Psi_rd_111227
Psidium cattleyanum	UDP-glycosyltransferase 88A1-like	Psi_rd_120882
Psidium cattleyanum	anthocyanidin 3-O-glucosyltransferase 2	Psi_rd_13096
Psidium cattleyanum	anthocyanidin 3-O-glucosyltransferase 2	Psi_rd_13100
Psidium cattleyanum	7-deoxyloganetin glucosyltransferase-like	Psi_rd_132210
Psidium cattleyanum	anthocyanidin 3-O-glucosyltransferase 2	Psi_rd_133083
Psidium cattleyanum	UDP-glucose iridoid glucosyltransferase	Psi_rd_133214
Psidium cattleyanum	UDP-glycosyltransferase 71K1	Psi_rd_139697
Psidium cattleyanum	anthocyanidin 3-O-glucosyltransferase 2-like	Psi_rd_139862
Psidium cattleyanum	UDP-glycosyltransferase 91A1-like	Psi_rd_156891
Psidium cattleyanum	UDP-glucuronosyl UDP-glucosyltransferase	Psi_rd_159686
Psidium cattleyanum	probable alpha,alpha-trehalose-phosphate synthase [UDP-forming] 11	Psi_rd_163842
Psidium cattleyanum	UDP-N-acetylglucosamine--N-acetylmuramyl-(pentapeptide) pyrophosphoryl-undecaprenol N-acetylglucosamine transferase	Psi_rd_165120
Psidium cattleyanum	UDP-glucuronate 4-epimerase 6	Psi_rd_165694
Psidium cattleyanum	UDP-glycosyltransferase 76C2	Psi_rd_168277

Species	**Annotation**	**Acronym**
Psidium cattleyanum	7-deoxyloganetin glucosyltransferase	Psi_rd_168764
Psidium cattleyanum	bifunctional UDP-glucose 4-epimerase and UDP-xylose 4-epimerase 1	Psi_rd_170511
Psidium cattleyanum	UDP-glycosyltransferase 92A1	Psi_rd_184154
Psidium cattleyanum	UDP-glycosyltransferase 73C3-like	Psi_rd_184196
Psidium cattleyanum	anthocyanidin 3-O-glucosyltransferase 7	Psi_rd_188302
Psidium cattleyanum	UDP-glycosyltransferase 74F2-like	Psi_rd_191946
Psidium cattleyanum	beta-D-glucosyl crocetin beta-1,6-glucosyltransferase-like	Psi_rd_192204
Psidium cattleyanum	probable UDP-arabinopyranose mutase 5 isoform X2	Psi_rd_192583
Psidium cattleyanum	anthocyanidin 3-O-glucosyltransferase 2	Psi_rd_196204
Psidium cattleyanum	UDP-arabinose 4-epimerase 1	Psi_rd_199936
Psidium cattleyanum	anthocyanidin 3-O-glucosyltransferase 2	Psi_rd_224630
Psidium cattleyanum	UDP-glycosyltransferase 89B2-like	Psi_rd_228853
Psidium cattleyanum	UDP-glycosyltransferase 91A1-like	Psi_rd_234661
Psidium cattleyanum	UDP-glycosyltransferase 73D1-like	Psi_rd_234961
Psidium cattleyanum	7-deoxyloganetin glucosyltransferase	Psi_rd_246003
Psidium cattleyanum	UDP glucose: glyco glucosyltransferase	Psi_rd_249367
Psidium cattleyanum	scopoletin glucosyltransferase	Psi_rd_268581
Psidium cattleyanum	cinnamate beta-D-glucosyltransferase-like	Psi_rd_268622
Psidium cattleyanum	beta-D-glucosyl crocetin beta-1,6-glucosyltransferase-like	Psi_rd_270515
Psidium cattleyanum	UDP-glucuronate 4-epimerase 1	Psi_rd_272811
Psidium cattleyanum	UDP-glycosyltransferase 74B1-like	Psi_rd_280712
Psidium cattleyanum	zeatin O-glucosyltransferase	Psi_rd_282936
Psidium cattleyanum	UDP-glycosyltransferase 83A1	Psi_rd_289980
Psidium cattleyanum	hydroquinone glucosyltransferase	Psi_rd_290204
Psidium cattleyanum	UDP-glycosyltransferase 91A1-like	Psi_rd_293058
Psidium cattleyanum	7-deoxyloganetin glucosyltransferase	Psi_rd_294405
Psidium cattleyanum	UDP-glycosyltransferase 74G1-like	Psi_rd_41485
Psidium cattleyanum	7-deoxyloganetin glucosyltransferase	Psi_rd_44820
Psidium cattleyanum	7-deoxyloganetin glucosyltransferase	Psi_rd_44823

Species	**Annotation**	**Acronym**
Psidium cattleyanum	probable alpha,alpha-trehalose-phosphate synthase [UDP-forming] 9	Psi_rd_4850
Psidium cattleyanum	UDP-glycosyltransferase 76F1	Psi_rd_60075
Psidium cattleyanum	UDP-arabinopyranose mutase 1	Psi_rd_62453
Psidium cattleyanum	UDP-glucose:flavonoid 3-O-	Psi_rd_65618
Psidium cattleyanum	7-deoxyloganetin glucosyltransferase	Psi_rd_68499
Psidium cattleyanum	UDP-glycosyltransferase 88B1	Psi_rd_77174
Psidium cattleyanum	bifunctional UDP-glucose 4-epimerase and UDP-xylose 4-epimerase 1	Psi_rd_82720
Psidium cattleyanum	UDP-glycosyltransferase 83A1	Psi_rd_87912
Psidium cattleyanum	hydroquinone glucosyltransferase-like	Psi_rd_93878
Vitis vinifera	GSVIVG01003960001 Org_Vvinifera peptide: GSVIVT01003960001 (1 of 2) PTHR11926//PTHR11926:SF199 - GLUCOSYL/GLUCURONOSYL TRANSFERASES // SUBFAMILY NOT NAMED (PAC:17818844)	Vvi_GSVIVG01003960001
Vitis vinifera	GSVIVG01004328001 Org_Vvinifera peptide: GSVIVT01004328001 (1 of 3) PTHR11926//PTHR11926:SF269 - GLUCOSYL/GLUCURONOSYL TRANSFERASES // SUBFAMILY NOT NAMED (PAC:17818993)	Vvi_GSVIVG01004328001
Vitis vinifera	GSVIVG01005733001 Org_Vvinifera peptide: GSVIVT01005733001 (1 of 4) PTHR11926:SF396 - UDP-GLYCOSYLTRANSFERASE 75B2 (PAC:17819581)	Vvi_GSVIVG01005733001
Vitis vinifera	GSVIVG01005737001 Org_Vvinifera peptide: GSVIVT01005737001 (1 of 2) K13691 - pathogen-inducible salicylic acid glucosyltransferase [EC:2.4.1.-] Glc b1-2 SA (SGT1) (PAC:17819584)	Vvi_GSVIVG01005737001
Vitis vinifera	GSVIVG01006042001 Org_Vvinifera peptide: GSVIVT01006042001 (1 of 6) 2.4.1.85 - Cyanohydrin beta-glucosyltransferase / Uridine diphosphoglucose:aldehyde cyanohydrin beta-glucosyltransferase (PAC:17819698)	Vvi_GSVIVG01006042001

Species	**Annotation**	**Acronym**
Vitis vinifera	GSVIVG01006043001 Org_Vvinifera peptide: GSVIVT01006043001 (1 of 1) PTHR11926//PTHR11926:SF218 - GLUCOSYL/GLUCURONOSYL TRANSFERASES // SUBFAMILY NOT NAMED (PAC:17819699)	Vvi_GSVIVG01006043001
Vitis vinifera	GSVIVG01006044001 Org_Vvinifera peptide: GSVIVT01006044001 (1 of 2) PF00201//PF01535 - UDP-glucoronosyl and UDP-glucosyl transferase (UDPGT) // PPR repeat (PPR) (PAC:17819700)	Vvi_GSVIVG01006044001
Vitis vinifera	GSVIVG01006626001 Org_Vvinifera peptide: GSVIVT01006626001 (1 of 3) PTHR11926//PTHR11926:SF269 - GLUCOSYL/GLUCURONOSYL TRANSFERASES // SUBFAMILY NOT NAMED (PAC:17819983)	Vvi_GSVIVG01006626001
Vitis vinifera	GSVIVG01007815001 Org_Vvinifera peptide: GSVIVT01007815001 (1 of 2) 2.4.1.195 - N-hydroxythioamide S-beta-glucosyltransferase / Uridine diphosphoglucose-thiohydroximate glucosyltransferase (PAC:17820543)	Vvi_GSVIVG01007815001
Vitis vinifera	GSVIVG01007892001 Org_Vvinifera peptide: GSVIVT01007892001 (1 of 10) 2.4.1.203 - Trans-zeatin O-beta-D-glucosyltransferase / Zeatin O-glucosyltransferase (PAC:17820602)	Vvi_GSVIVG01007892001
Vitis vinifera	GSVIVG01007896001 Org_Vvinifera peptide: GSVIVT01007896001 (1 of 6) 2.4.1.85 - Cyanohydrin beta-glucosyltransferase / Uridine diphosphoglucose:aldehyde cyanohydrin beta-glucosyltransferase (PAC:17820603)	Vvi_GSVIVG01007896001
Vitis vinifera	GSVIVG01007898001 Org_Vvinifera peptide: GSVIVT01007898001 (1 of 10) 2.4.1.203 - Trans-zeatin O-beta-D-glucosyltransferase / Zeatin O-glucosyltransferase (PAC:17820604)	Vvi_GSVIVG01007898001

Species	**Annotation**	**Acronym**
Vitis vinifera	GSVIVG01007899001 Org_Vvinifera peptide: GSVIVT01007899001 (1 of 10) 2.4.1.203 - Trans-zeatin O-beta-D-glucosyltransferase / Zeatin O-glucosyltransferase (PAC:17820605)	Vvi_GSVIVG01007899001
Vitis vinifera	GSVIVG01008168001 Org_Vvinifera peptide: GSVIVT01008168001 (1 of 2) PTHR11926:SF253 - UDP-GLYCOSYLTRANSFERASE 89B1 (PAC:17820824)	Vvi_GSVIVG01008168001
Vitis vinifera	GSVIVG01009062001 Org_Vvinifera peptide: GSVIVT01009062001 (1 of 4) PTHR11926//PTHR11926:SF302 - GLUCOSYL/GLUCURONOSYL TRANSFERASES // SUBFAMILY NOT NAMED (PAC:17821556)	Vvi_GSVIVG01009062001
Vitis vinifera	GSVIVG01009449001 Org_Vvinifera peptide: GSVIVT01009449001 (1 of 1) PTHR11926//PTHR11926:SF384 - GLUCOSYL/GLUCURONOSYL TRANSFERASES // SUBFAMILY NOT NAMED (PAC:17821861)	Vvi_GSVIVG01009449001
Vitis vinifera	GSVIVG01009658001 Org_Vvinifera peptide: GSVIVT01009658001 (1 of 1) PTHR11926:SF381 - UDP-GLYCOSYLTRANSFERASE 72B1 (PAC:17822028)	Vvi_GSVIVG01009658001
Vitis vinifera	GSVIVG01010098001 Org_Vvinifera peptide: GSVIVT01010098001 (1 of 1) PTHR11926:SF149 - UDP-GLYCOSYLTRANSFERASE 82A1 (PAC:17822384)	Vvi_GSVIVG01010098001
Vitis vinifera	GSVIVG01010812001 Org_Vvinifera peptide: GSVIVT01010812001 (1 of 9) 2.4.1.323 - 7-deoxyloganetic acid glucosyltransferase (PAC:17822871)	Vvi_GSVIVG01010812001

Species	**Annotation**	**Acronym**
Vitis vinifera	GSVIVG01010817001 Org_Vvinifera peptide: GSVIVT01010817001 (1 of 9) 2.4.1.323 - 7-deoxyloganetic acid glucosyltransferase (PAC:17822875)	Vvi_GSVIVG01010817001
Vitis vinifera	GSVIVG01012577001 Org_Vvinifera peptide: GSVIVT01012577001 (1 of 5) PTHR11926//PTHR11926:SF118 - GLUCOSYL/GLUCURONOSYL TRANSFERASES // SUBFAMILY NOT NAMED (PAC:17824133)	Vvi_GSVIVG01012577001
Vitis vinifera	GSVIVG01012581001 Org_Vvinifera peptide: GSVIVT01012581001 (1 of 4) K12356 - coniferyl-alcohol glucosyltransferase (UGT72E) (PAC:17824134)	Vvi_GSVIVG01012581001
Vitis vinifera	GSVIVG01012978001 Org_Vvinifera peptide: GSVIVT01012978001 (1 of 9) 2.4.1.91 - Flavonol 3-O-glucosyltransferase / UDP-glucose flavonol 3-O-glucosyltransferase (PAC:17824390)	Vvi_GSVIVG01012978001
Vitis vinifera	GSVIVG01014265001 Org_Vvinifera peptide: GSVIVT01014265001 (1 of 6) 2.4.2.51 - Anthocyanidin 3-O-glucoside 2'''-O-xylosyltransferase (PAC:17825286)	Vvi_GSVIVG01014265001
Vitis vinifera	GSVIVG01014267001 Org_Vvinifera peptide: GSVIVT01014267001 (1 of 6) 2.4.2.51 - Anthocyanidin 3-O-glucoside 2'''-O-xylosyltransferase (PAC:17825287)	Vvi_GSVIVG01014267001

Species	**Annotation**	**Acronym**
Vitis vinifera	GSVIVG01014269001 Org_Vvinifera peptide: GSVIVT01014269001 (1 of 6) 2.4.2.51 - Anthocyanidin 3-O-glucoside 2'''-O-xylosyltransferase (PAC:17825289)	Vvi_GSVIVG01014269001
Vitis vinifera	GSVIVG01014270001 Org_Vvinifera peptide: GSVIVT01014270001 (1 of 6) 2.4.2.51 - Anthocyanidin 3-O-glucoside 2'''-O-xylosyltransferase (PAC:17825290)	Vvi_GSVIVG01014270001
Vitis vinifera	GSVIVG01014272001 Org_Vvinifera peptide: GSVIVT01014272001 (1 of 6) 2.4.2.51 - Anthocyanidin 3-O-glucoside 2'''-O-xylosyltransferase (PAC:17825292)	Vvi_GSVIVG01014272001
Vitis vinifera	GSVIVG01015164001 Org_Vvinifera peptide: GSVIVT01015164001 (1 of 2) PTHR11926:SF360 - UDP-GLYCOSYLTRANSFERASE 91A1-RELATED (PAC:17825963)	Vvi_GSVIVG01015164001
Vitis vinifera	GSVIVG01015743001 Org_Vvinifera peptide: GSVIVT01015743001 (1 of 6) PTHR11926//PTHR11926:SF286 - GLUCOSYL/GLUCURONOSYL TRANSFERASES // SUBFAMILY NOT NAMED (PAC:17826417)	Vvi_GSVIVG01015743001
Vitis vinifera	GSVIVG01016138001 Org_Vvinifera peptide: GSVIVT01016138001 (1 of 2) PTHR11926:SF250 - UDP-GLYCOSYLTRANSFERASE 90A1-RELATED (PAC:17826644)	Vvi_GSVIVG01016138001

Species	Annotation	Acronym
Vitis vinifera	GSVIVG01016402001 Org_Vvinifera peptide: GSVIVT01016402001 (1 of 9) 2.4.1.323 - 7-deoxyloganetic acid glucosyltransferase (PAC:17826837)	Vvi_GSVIVG01016402001
Vitis vinifera	GSVIVG01016409001 Org_Vvinifera peptide: GSVIVT01016409001 (1 of 9) 2.4.1.323 - 7-deoxyloganetic acid glucosyltransferase (PAC:17826843)	Vvi_GSVIVG01016409001
Vitis vinifera	GSVIVG01016417001 Org_Vvinifera peptide: GSVIVT01016417001 (1 of 9) 2.4.1.323 - 7-deoxyloganetic acid glucosyltransferase (PAC:17826849)	Vvi_GSVIVG01016417001
Vitis vinifera	GSVIVG01016422001 Org_Vvinifera peptide: GSVIVT01016422001 (1 of 9) 2.4.1.323 - 7-deoxyloganetic acid glucosyltransferase (PAC:17826852)	Vvi_GSVIVG01016422001
Vitis vinifera	GSVIVG01016425001 Org_Vvinifera peptide: GSVIVT01016425001 (1 of 2) PTHR11926:SF204 - UDP-GLYCOSYLTRANSFERASE 76C1-RELATED (PAC:17826854)	Vvi_GSVIVG01016425001
Vitis vinifera	GSVIVG01016427001 Org_Vvinifera peptide: GSVIVT01016427001 (1 of 2) PTHR11926:SF204 - UDP-GLYCOSYLTRANSFERASE 76C1-RELATED (PAC:17826856)	Vvi_GSVIVG01016427001

Species	**Annotation**	**Acronym**
Vitis vinifera	GSVIVG01017303001 Org_Vvinifera peptide: GSVIVT01017303001 (1 of 2) PTHR11926//PTHR11926:SF224 - GLUCOSYL/GLUCURONOSYL TRANSFERASES // SUBFAMILY NOT NAMED (PAC:17827551)	Vvi_GSVIVG01017303001
Vitis vinifera	GSVIVG01018499001 Org_Vvinifera peptide: GSVIVT01018499001 (1 of 4) K12356 - coniferyl-alcohol glucosyltransferase (UGT72E) (PAC:17828428)	Vvi_GSVIVG01018499001
Vitis vinifera	GSVIVG01019017001 Org_Vvinifera peptide: GSVIVT01019017001 (1 of 4) K12356 - coniferyl-alcohol glucosyltransferase (UGT72E) (PAC:17828794)	Vvi_GSVIVG01019017001
Vitis vinifera	GSVIVG01019508001 Org_Vvinifera peptide: GSVIVT01019508001 (1 of 1) K14299 - nucleoporin SEH1 (SEH1) (PAC:17829116)	Vvi_GSVIVG01019508001
Vitis vinifera	GSVIVG01020177001 Org_Vvinifera peptide: GSVIVT01020177001 (1 of 2) 2.4.1.195 - N-hydroxythioamide S-beta-glucosyltransferase / Uridine diphosphoglucose-thiohydroximate glucosyltransferase (PAC:17829644)	Vvi_GSVIVG01020177001
Vitis vinifera	GSVIVG01023005001 Org_Vvinifera peptide: GSVIVT01023005001 (1 of 3) PTHR11926//PTHR11926:SF184 - GLUCOSYL/GLUCURONOSYL TRANSFERASES // SUBFAMILY NOT NAMED (PAC:17831622)	Vvi_GSVIVG01023005001

Species	**Annotation**	**Acronym**
Vitis vinifera	GSVIVG01024419001 Org_Vvinifera peptide: GSVIVT01024419001 (1 of 1) K12930 - anthocyanidin 3-O-glucosyltransferase (BZ1) (PAC:17832626)	Vvi_GSVIVG01024419001_RefSeq
Vitis vinifera	GSVIVG01024649001 Org_Vvinifera peptide: GSVIVT01024649001 (1 of 6) 2.4.1.85 - Cyanohydrin beta-glucosyltransferase / Uridine diphosphoglucose:aldehyde cyanohydrin beta-glucosyltransferase (PAC:17832801)	Vvi_GSVIVG01024649001
Vitis vinifera	GSVIVG01024653001 Org_Vvinifera peptide: GSVIVT01024653001 (1 of 2) 2.4.1.120 - Sinapate 1-glucosyltransferase (PAC:17832805)	Vvi_GSVIVG01024653001
Vitis vinifera	GSVIVG01024655001 Org_Vvinifera peptide: GSVIVT01024655001 (1 of 14) 2.4.1.324 - 7-deoxyloganetin glucosyltransferase / UGT85A24 (PAC:17832806)	Vvi_GSVIVG01024655001
Vitis vinifera	GSVIVG01024656001 Org_Vvinifera peptide: GSVIVT01024656001 (1 of 8) PTHR11926:SF176 - UDP-GLYCOSYLTRANSFERASE 87A2 (PAC:17832807)	Vvi_GSVIVG01024656001
Vitis vinifera	GSVIVG01024836001 Org_Vvinifera peptide: GSVIVT01024836001 (1 of 2) PTHR11926:SF102 - GLUCOSYLTRANSFERASE-LIKE PROTEIN-RELATED (PAC:17832949)	Vvi_GSVIVG01024836001

Species	Annotation	Acronym
Vitis vinifera	GSVIVG01025320001 Org_Vvinifera peptide: GSVIVT01025320001 (1 of 1) PTHR11926//PTHR11926:SF392 - GLUCOSYL/GLUCURONOSYL TRANSFERASES // SUBFAMILY NOT NAMED (PAC:17833340)	Vvi_GSVIVG01025320001
Vitis vinifera	GSVIVG01025724001 Org_Vvinifera peptide: GSVIVT01025724001 (1 of 2) PTHR11926//PTHR11926:SF383 - GLUCOSYL/GLUCURONOSYL TRANSFERASES // SUBFAMILY NOT NAMED (PAC:17833660)	Vvi_GSVIVG01025724001
Vitis vinifera	GSVIVG01026049001 Org_Vvinifera peptide: GSVIVT01026049001 (1 of 2) PTHR11926:SF343 - UDP-GLYCOSYLTRANSFERASE 88A1 (PAC:17833904)	Vvi_GSVIVG01026049001
Vitis vinifera	GSVIVG01026054001 Org_Vvinifera peptide: GSVIVT01026054001 (1 of 1) PTHR11926//PTHR11926:SF325 - GLUCOSYL/GLUCURONOSYL TRANSFERASES // SUBFAMILY NOT NAMED (PAC:17833909)	Vvi_GSVIVG01026054001
Vitis vinifera	GSVIVG01026464001 Org_Vvinifera peptide: GSVIVT01026464001 (1 of 2) PTHR11926//PTHR11926:SF342 - GLUCOSYL/GLUCURONOSYL TRANSFERASES // SUBFAMILY NOT NAMED (PAC:17834163)	Vvi_GSVIVG01026464001
Vitis vinifera	GSVIVG01026986001 Org_Vvinifera peptide: GSVIVT01026986001 (1 of 2) PTHR11926//PTHR11926:SF310 - GLUCOSYL/GLUCURONOSYL TRANSFERASES // SUBFAMILY NOT NAMED (PAC:17834525)	Vvi_GSVIVG01026986001

Species	**Annotation**	**Acronym**
Vitis vinifera	GSVIVG01026987001 Org_Vvinifera peptide: GSVIVT01026987001 (1 of 2) PTHR11926//PTHR11926:SF310 - GLUCOSYL/GLUCURONOSYL TRANSFERASES // SUBFAMILY NOT NAMED (PAC:17834526)	Vvi_GSVIVG01026987001
Vitis vinifera	GSVIVG01027064001 Org_Vvinifera peptide: GSVIVT01027064001 (1 of 2) K08237 - hydroquinone glucosyltransferase [EC:2.4.1.218] Glc b1- hydroquinone (E2.4.1.218) (PAC:17834586)	Vvi_GSVIVG01027064001
Vitis vinifera	GSVIVG01028807001 Org_Vvinifera peptide: GSVIVT01028807001 (1 of 5) PTHR11926//PTHR11926:SF118 - GLUCOSYL/GLUCURONOSYL TRANSFERASES // SUBFAMILY NOT NAMED (PAC:17835819)	Vvi_GSVIVG01028807001
Vitis vinifera	GSVIVG01028961001 Org_Vvinifera peptide: GSVIVT01028961001 (1 of 5) PTHR11926//PTHR11926:SF118 - GLUCOSYL/GLUCURONOSYL TRANSFERASES // SUBFAMILY NOT NAMED (PAC:17835940)	Vvi_GSVIVG01028961001
Vitis vinifera	GSVIVG01029122001 Org_Vvinifera peptide: GSVIVT01029122001 (1 of 8) 2.4.1.115//2.4.1.91 - Anthocyanidin 3-O-glucosyltransferase / Uridine diphosphoglucose-anthocyanidin 3-O-glucosyltransferase // Flavonol 3-O-glucosyltransferase / UDP-glucose flavonol 3-O-glucosyltransferase (PAC:17836046)	Vvi_GSVIVG01029122001
Vitis vinifera	GSVIVG01029126001 Org_Vvinifera peptide: GSVIVT01029126001 (1 of 8) 2.4.1.115//2.4.1.91 - Anthocyanidin 3-O-glucosyltransferase / Uridine diphosphoglucose-anthocyanidin 3-O-glucosyltransferase // Flavonol 3-O-glucosyltransferase / UDP-glucose flavonol 3-O-glucosyltransferase (PAC:17836049)	Vvi_GSVIVG01029126001

Species	**Annotation**	**Acronym**
Vitis vinifera	GSVIVG01029129001 Org_Vvinifera peptide: GSVIVT01029129001 (1 of 8) 2.4.1.115//2.4.1.91 - Anthocyanidin 3-O-glucosyltransferase / Uridine diphosphoglucose-anthocyanidin 3-O-glucosyltransferase // Flavonol 3-O-glucosyltransferase / UDP-glucose flavonol 3-O-glucosyltransferase (PAC:17836050)	Vvi_GSVIVG01029129001
Vitis vinifera	GSVIVG01029671001 Org_Vvinifera peptide: GSVIVT01029671001 (1 of 3) PTHR11926//PTHR11926:SF184 - GLUCOSYL/GLUCURONOSYL TRANSFERASES // SUBFAMILY NOT NAMED (PAC:17836408)	Vvi_GSVIVG01029671001
Vitis vinifera	GSVIVG01029680001 Org_Vvinifera peptide: GSVIVT01029680001 (1 of 3) PTHR11926//PTHR11926:SF184 - GLUCOSYL/GLUCURONOSYL TRANSFERASES // SUBFAMILY NOT NAMED (PAC:17836410)	Vvi_GSVIVG01029680001
Vitis vinifera	GSVIVG01031515001 Org_Vvinifera peptide: GSVIVT01031515001 (1 of 2) 2.4.1.298 - Anthocyanidin 3-O-glucoside 5-O-glucosyltransferase (PAC:17837687)	Vvi_GSVIVG01031515001
Vitis vinifera	GSVIVG01031585001 Org_Vvinifera peptide: GSVIVT01031585001 (1 of 2) PTHR11926//PTHR11926:SF224 - GLUCOSYL/GLUCURONOSYL TRANSFERASES // SUBFAMILY NOT NAMED (PAC:17837737)	Vvi_GSVIVG01031585001
Vitis vinifera	GSVIVG01031592001 Org_Vvinifera peptide: GSVIVT01031592001 (1 of 2) 2.4.1.298 - Anthocyanidin 3-O-glucoside 5-O-glucosyltransferase (PAC:17837743)	Vvi_GSVIVG01031592001

Species	**Annotation**	**Acronym**
Vitis vinifera	GSVIVG01031613001 Org_Vvinifera peptide: GSVIVT01031613001 (1 of 4) PTHR11926:SF98 - UDP-GLYCOSYLTRANSFERASE 75B1-RELATED (PAC:17837755)	Vvi_GSVIVG01031613001
Vitis vinifera	GSVIVG01031615001 Org_Vvinifera peptide: GSVIVT01031615001 (1 of 10) 2.4.1.271 - Crocetin glucosyltransferase / UGT75L6 (PAC:17837757)	Vvi_GSVIVG01031615001
Vitis vinifera	GSVIVG01031678001 Org_Vvinifera peptide: GSVIVT01031678001 (1 of 1) PTHR11926//PTHR11926:SF298 - GLUCOSYL/GLUCURONOSYL TRANSFERASES // SUBFAMILY NOT NAMED (PAC:17837801)	Vvi_GSVIVG01031678001
Vitis vinifera	GSVIVG01032925001 Org_Vvinifera peptide: GSVIVT01032925001 (1 of 1) PTHR11926//PTHR11926:SF265 - GLUCOSYL/GLUCURONOSYL TRANSFERASES // SUBFAMILY NOT NAMED (PAC:17838773)	Vvi_GSVIVG01032925001
Vitis vinifera	GSVIVG01032927001 Org_Vvinifera peptide: GSVIVT01032927001 (1 of 10) 2.4.1.203 - Trans-zeatin O-beta-D-glucosyltransferase / Zeatin O-glucosyltransferase (PAC:17838775)	Vvi_GSVIVG01032927001
Vitis vinifera	GSVIVG01032930001 Org_Vvinifera peptide: GSVIVT01032930001 (1 of 10) 2.4.1.203 - Trans-zeatin O-beta-D-glucosyltransferase / Zeatin O-glucosyltransferase (PAC:17838776)	Vvi_GSVIVG01032930001

Species	**Annotation**	**Acronym**
Vitis vinifera	GSVIVG01033243001 Org_Vvinifera peptide: GSVIVT01033243001 (1 of 1) K13495 - cis-zeatin O-glucosyltransferase (CISZOG) (PAC:17838993)	Vvi_GSVIVG01033243001
Vitis vinifera	GSVIVG01033701001 Org_Vvinifera peptide: GSVIVT01033701001 (1 of 2) K13496 - UDP-glucosyl transferase 73C [EC:2.4.1.-] Glc - brassinosteroid (UGT73C) (PAC:17839366)	Vvi_GSVIVG01033701001
Vitis vinifera	GSVIVG01036732001 Org_Vvinifera peptide: GSVIVT01036732001 (1 of 1) PTHR11926//PTHR11926:SF267 - GLUCOSYL/GLUCURONOSYL TRANSFERASES // SUBFAMILY NOT NAMED (PAC:17841586)	Vvi_GSVIVG01036732001
Vitis vinifera	GSVIVG01037410001 Org_Vvinifera peptide: GSVIVT01037410001 (1 of 8) 2.4.1.115//2.4.1.91 - Anthocyanidin 3-O-glucosyltransferase / Uridine diphosphoglucose-anthocyanidin 3-O-glucosyltransferase // Flavonol 3-O-glucosyltransferase / UDP-glucose flavonol 3-O-glucosyltransferase (PAC:17842040)	Vvi_GSVIVG01037410001
Vitis vinifera	GSVIVG01037411001 Org_Vvinifera peptide: GSVIVT01037411001 (1 of 8) 2.4.1.115//2.4.1.91 - Anthocyanidin 3-O-glucosyltransferase / Uridine diphosphoglucose-anthocyanidin 3-O-glucosyltransferase // Flavonol 3-O-glucosyltransferase / UDP-glucose flavonol 3-O-glucosyltransferase (PAC:17842041)	Vvi_GSVIVG01037411001
Vitis vinifera	GSVIVG01037413001 Org_Vvinifera peptide: GSVIVT01037413001 (1 of 8) 2.4.1.115//2.4.1.91 - Anthocyanidin 3-O-glucosyltransferase / Uridine diphosphoglucose-anthocyanidin 3-O-glucosyltransferase // Flavonol 3-O-glucosyltransferase / UDP-glucose flavonol 3-O-glucosyltransferase (PAC:17842043)	Vvi_GSVIVG01037413001

Species	**Annotation**	**Acronym**
Vitis vinifera	GSVIVG01038200001 Org_Vvinifera peptide: GSVIVT01038200001 (1 of 2) K13691 - pathogen-inducible salicylic acid glucosyltransferase [EC:2.4.1.-] Glc b1-2 SA (SGT1) (PAC:17842581)	Vvi_GSVIVG01038200001
Vitis vinifera	GSVIVG01038204001 Org_Vvinifera peptide: GSVIVT01038204001 (1 of 4) PTHR11926:SF279 - UDP-GLYCOSYLTRANSFERASE 74C1-RELATED (PAC:17842585)	Vvi_GSVIVG01038204001
Vitis vinifera	GSVIVG01038206001 Org_Vvinifera peptide: GSVIVT01038206001 (1 of 2) PTHR11926:SF271 - UDP-GLYCOSYLTRANSFERASE 74D1 (PAC:17842587)	Vvi_GSVIVG01038206001
Vitis vinifera	GSVIVG01038207001 Org_Vvinifera peptide: GSVIVT01038207001 (1 of 4) PTHR11926:SF279 - UDP-GLYCOSYLTRANSFERASE 74C1-RELATED (PAC:17842588)	Vvi_GSVIVG01038207001
Vitis vinifera	GSVIVG01038209001 Org_Vvinifera peptide: GSVIVT01038209001 (1 of 4) PTHR11926:SF279 - UDP-GLYCOSYLTRANSFERASE 74C1-RELATED (PAC:17842590)	Vvi_GSVIVG01038209001
