# Supplementary material for: Transcriptomics analysis of Psidium cattleyanum Sabine (Myrtaceae) unveil potential genes involved in fruit pigmentation
Source: Genet Mol Biol. 2020 Apr 27;43(2):e20190255. doi: 10.1590/1678-4685-GMB-2019-0255 (PMC7199922; doi:10.1590/1678-4685-GMB-2019-0255)
Supplement: Table S7 [file 1415-4757-GMB-43-2-e20190255-s8.pdf]

Supplementary material to: Transcriptomics analysis of *Psidium cattleyanum* Sabine (Myrtaceae) unveil potential genes involved in fruit pigmentation

Table S7 - Species information used to perform anthocyanidin synthase (ANS,) phylogenetic analysis.

Species	Annotation	Acronym
Eucalyptus grandis	Eucgr.A00518 Org_Egrandis peptide: Eucgr.A00518.1.p (1 of 3) PTHR10209:SF134 - 2-OXOGLUTARATE (2OG) AND FE(II)-DEPENDENT OXYGENASE-LIKE PROTEIN (PAC:32047697)	Egr_A00518
Eucalyptus grandis	Eucgr.C00494 Org_Egrandis peptide: Eucgr.C00494.1.p (1 of 4) PTHR10209:SF107 - 2-OXOGLUTARATE (2OG) AND FE(II)-DEPENDENT OXYGENASE-LIKE PROTEIN-RELATED (PAC:32039882)	Egr_C00494
Eucalyptus grandis	Eucgr.D01945 Org_Egrandis peptide: Eucgr.D01945.1.p (1 of 1) K05277 - leucoanthocyanidin dioxygenase (E1.14.11.19) (PAC:32050146)	Egr_D01945_RefSeq
Eucalyptus grandis	Eucgr.E01689 Org_Egrandis peptide: Eucgr.E01689.1.p (1 of 4) K05278 - flavonol synthase (FLS) (PAC:32031614)	Egr_E01689

Species	Annotation	Acronym
Eucalyptus grandis	Eucgr.E02879 Org_Egrandis peptide: Eucgr.E02879.1.p (1 of 27) 1.14.11.32 - Codeine 3-O-demethylase / Codeine O-demethylase (PAC:32030408)	Egr_E02879
Eucalyptus grandis	Eucgr.E02880 Org_Egrandis peptide: Eucgr.E02880.1.p (1 of 27) 1.14.11.32 - Codeine 3-O-demethylase / Codeine O-demethylase (PAC:32030562)	Egr_E02880
Eucalyptus grandis	Eucgr.E02881 Org_Egrandis peptide: Eucgr.E02881.1.p (1 of 27) 1.14.11.32 - Codeine 3-O-demethylase / Codeine O-demethylase (PAC:32028404)	Egr_E02881
Eucalyptus grandis	Eucgr.E02882 Org_Egrandis peptide: Eucgr.E02882.1.p (1 of 27) 1.14.11.32 - Codeine 3-O-demethylase / Codeine O-demethylase (PAC:32031268)	Egr_E02882
Eucalyptus grandis	Eucgr.E02888 Org_Egrandis peptide: Eucgr.E02888.1.p (1 of 27) 1.14.11.32 - Codeine 3-O-demethylase / Codeine O-demethylase (PAC:32029771)	Egr_E02888
Eucalyptus grandis	Eucgr.E02892 Org_Egrandis peptide: Eucgr.E02892.1.p (1 of 27) 1.14.11.32 - Codeine 3-O-demethylase / Codeine O-demethylase (PAC:32028428)	Egr_E02892

Species	**Annotation**	**Acronym**
Eucalyptus grandis	Eucgr.E02897 Org_Egrandis peptide: Eucgr.E02897.1.p (1 of 27) 1.14.11.32 - Codeine 3-O-demethylase / Codeine O-demethylase (PAC:32030531)	Egr_E02897
Eucalyptus grandis	Eucgr.E02902 Org_Egrandis peptide: Eucgr.E02902.1.p (1 of 27) 1.14.11.32 - Codeine 3-O-demethylase / Codeine O-demethylase (PAC:32029292)	Egr_E02902
Eucalyptus grandis	Eucgr.E02903 Org_Egrandis peptide: Eucgr.E02903.1.p (1 of 27) 1.14.11.32 - Codeine 3-O-demethylase / Codeine O-demethylase (PAC:32028392)	Egr_E02903
Eucalyptus grandis	Eucgr.F03761 Org_Egrandis peptide: Eucgr.F03761.1.p (1 of 4) K05278 - flavonol synthase (FLS) (PAC:32056383)	Egr_F03761
Eucalyptus grandis	Eucgr.F03763 Org_Egrandis peptide: Eucgr.F03763.1.p (1 of 4) K05278 - flavonol synthase (FLS) (PAC:32057279)	Egr_F03763
Eucalyptus grandis	Eucgr.G03066 Org_Egrandis peptide: Eucgr.G03066.1.p (1 of 3) PTHR10209:SF134 - 2-OXOGLUTARATE (2OG) AND FE(II)-DEPENDENT OXYGENASE-LIKE PROTEIN (PAC:32071295)	Egr_G03066

Species	**Annotation**	**Acronym**
Eucalyptus grandis	Eucgr.K02978 Org_Egrandis peptide: Eucgr.K02978.1.p (1 of 3) PTHR10209:SF134 - 2-OXOGLUTARATE (2OG) AND FE(II)-DEPENDENT OXYGENASE-LIKE PROTEIN (PAC:32069092)	Egr_K02978
Eucalyptus grandis	Eucgr.L00738 Org_Egrandis peptide: Eucgr.L00738.1.p (1 of 1) PTHR10209:SF165 - FLAVONOL SYNTHASE 3-RELATED (PAC:32041102)	Egr_L00738
Vitis vinifera	GSVIVG01008907001 Org_Vvinifera peptide: GSVIVT01008907001 (1 of 1) PTHR10209//PTHR10209:SF213 - OXIDOREDUCTASE, 2OG-FE II OXYGENASE FAMILY PROTEIN // SUBFAMILY NOT NAMED (PAC:17821428)	Vvi_GSVIVG01008907001
Vitis vinifera	GSVIVG01008914001 Org_Vvinifera peptide: GSVIVT01008914001 (1 of 2) 1.14.11.23//1.14.11.9 - Flavonol synthase / FLS // Flavanone 3-dioxygenase / Naringenin,2-oxoglutarate:oxygen oxidoreductase (3-hydroxylating) (PAC:17821434)	Vvi_GSVIVG01008914001
Vitis vinifera	GSVIVG01019892001 Org_Vvinifera peptide: GSVIVT01019892001 (1 of 1) K05277 - leucoanthocyanidin dioxygenase (E1.14.11.19) (PAC:17829430)	Vvi_GSVIVG01019892001_RefSeq

Species	Annotation	Acronym
Vitis vinifera	GSVIVG01032809001 Org_Vvinifera peptide: GSVIVT01032809001 (1 of 2) 1.14.11.19 - Leucocyanidin oxygenase / Leucoanthocyanidin dioxygenase (PAC:17838686)	Vvi_GSVIVG01032809001
Citrus sinensis	orange1.1g017934m.g Org_Csinensis peptide: orange1.1g017934m (1 of 3) 1.14.11.19 - Leucocyanidin oxygenase / Leucoanthocyanidin dioxygenase (PAC:18131621)	Csi_1g017934
Citrus sinensis	orange1.1g018097m.g Org_Csinensis peptide: orange1.1g018097m (1 of 3) 1.14.11.19 - Leucocyanidin oxygenase / Leucoanthocyanidin dioxygenase (PAC:18138296)	Csi_1g018097
Citrus sinensis	orange1.1g018369m.g Org_Csinensis peptide: orange1.1g018369m (1 of 1) K05277 - leucoanthocyanidin dioxygenase (E1.14.11.19) (PAC:18120419)	Csi_1g018369_RefSeq
Citrus sinensis	orange1.1g018466m.g Org_Csinensis peptide: orange1.1g018466m (1 of 1) PTHR10209:SF226 - 2-OXOGLUTARATE (2OG) AND FE(II)-DEPENDENT OXYGENASE SUPERFAMILY PROTEIN (PAC:18135670)	Csi_1g018466
Citrus sinensis	orange1.1g019717m.g Org_Csinensis peptide: orange1.1g019717m (1 of 3) K05278 - flavonol synthase (FLS) (PAC:18095158)	Csi_1g019717

Species	**Annotation**	**Acronym**
Citrus sinensis	orange1.1g019857m.g Org_Csinensis peptide: orange1.1g019857m (1 of 3) K05278 - flavonol synthase (FLS) (PAC:18103697)	Csi_1g019857
Citrus sinensis	orange1.1g044975m.g Org_Csinensis peptide: orange1.1g044975m (1 of 3) K05278 - flavonol synthase (FLS) (PAC:18108323)	Csi_1g044975
Prunus persica	Prupe.1G502300 Org_Ppersica peptide: Prupe.1G502300.1.p (1 of 5) K05278 - flavonol synthase (FLS) (PAC:32115630)	Ppe_1G502300
Prunus persica	Prupe.1G502500 Org_Ppersica peptide: Prupe.1G502500.1.p (1 of 5) K05278 - flavonol synthase (FLS) (PAC:32119114)	Ppe_1G502500
Prunus persica	Prupe.1G502700 Org_Ppersica peptide: Prupe.1G502700.1.p (1 of 2) 1.14.11.23//1.14.11.9 - Flavonol synthase / FLS // Flavanone 3-dioxygenase / Naringenin,2-oxoglutarate:oxygen oxidoreductase (3-hydroxylating) (PAC:32116450)	Ppe_1G502700
Prunus persica	Prupe.1G502800 Org_Ppersica peptide: Prupe.1G502800.1.p (1 of 2) 1.14.11.23//1.14.11.9 - Flavonol synthase / FLS // Flavanone 3-dioxygenase / Naringenin,2-oxoglutarate:oxygen oxidoreductase (3-hydroxylating) (PAC:32114227)	Ppe_1G502800

Species	**Annotation**	**Acronym**
Prunus persica	Prupe.2G250900 Org_Ppersica peptide: Prupe.2G250900.1.p (1 of 2) PTHR10209:SF134 - 2-OXOGLUTARATE (2OG) AND FE(II)-DEPENDENT OXYGENASE-LIKE PROTEIN (PAC:32077589)	Ppe_1G250900
Prunus persica	Prupe.3G183300 Org_Ppersica peptide: Prupe.3G183300.1.p (1 of 1) PTHR10209:SF226 - 2-OXOGLUTARATE (2OG) AND FE(II)-DEPENDENT OXYGENASE SUPERFAMILY PROTEIN (PAC:32106140)	Ppe_1G183300
Prunus persica	Prupe.5G043900 Org_Ppersica peptide: Prupe.5G043900.1.p (1 of 4) 4.2.1.78 - (S)-norcoclaurine synthase / (S)-norlaudanosoline synthase (PAC:32099136)	Ppe_1G043900
Prunus persica	Prupe.5G086700 Org_Ppersica peptide: Prupe.5G086700.1.p (1 of 1) K05277 - leucoanthocyanidin dioxygenase (E1.14.11.19) (PAC:32098744)	Ppe_1G086700_RefSeq
Prunus persica	Prupe.6G278400 Org_Ppersica peptide: Prupe.6G278400.1.p (1 of 3) 1.14.11.19 - Leucocyanidin oxygenase / Leucoanthocyanidin dioxygenase (PAC:32085823)	Ppe_1G278400
Prunus persica	Prupe.I003900 Org_Ppersica peptide: Prupe.I003900.1.p (1 of 5) K05278 - flavonol synthase (FLS) (PAC:32073833)	Ppe_1G003900
Psidium cattleyanum	Leucoanthocyanidin dioxygenase	Psi_yw_156616
Psidium cattleyanum	Leucoanthocyanidin dioxygenase	Psi_yw_103225

Species	**Annotation**	**Acronym**
Psidium cattleyanum	Leucoanthocyanidin dioxygenase	Psi_yw_90440
Psidium cattleyanum	Leucoanthocyanidin dioxygenase	Psi_rd_165087
Psidium cattleyanum	Leucoanthocyanidin dioxygenase	Psi_rd_159291
Psidium cattleyanum	Leucoanthocyanidin dioxygenase	Psi_rd_226347
