# Supplementary material for: Transcriptomics analysis of Psidium cattleyanum Sabine (Myrtaceae) unveil potential genes involved in fruit pigmentation
Source: Genet Mol Biol. 2020 Apr 27;43(2):e20190255. doi: 10.1590/1678-4685-GMB-2019-0255 (PMC7199922; doi:10.1590/1678-4685-GMB-2019-0255)
Supplement: Table S8 [file 1415-4757-GMB-43-2-e20190255-s9.pdf]

Supplementary material to: Transcriptomics analysis of *Psidium cattleyanum* Sabine (Myrtaceae) unveil potential genes involved in fruit pigmentation

Table S8 - Top 100 differential gene expression between Leaf vs Unripe fruit in yellow morphotype.

Cluster	Unigenes	Annotation	log2FoldChange	padj
Cluster-22812.17420	Psi-yw-149956	Hydrophobic seed protein	-10,22395449	3,523869E-54
Cluster-1200.11	Psi-yw-194351	developmental SEPALLATA 1-like	-10,02003465	1,085055E-54
Cluster-13454.14	Psi-yw-274869	No recognize	-10,01245716	2,297938E-48
Cluster-3079.40	Psi-yw-254028	expansin 2	-9,954122097	3,379039E-76
Cluster-22812.17300	Psi-yw-246143	Hydrophobic seed protein	-9,886594187	9,128365E-75
Cluster-22947.1	Psi-yw-265120	truncated transcription factor CAULIFLOWER A isoform X1	-9,730198317	5,058590E-71
Cluster-1200.9	Psi-yw-285951	SRF-type transcription factor (DNA-binding and dimerisation domain) / K-box region	-9,571508197	1,531646E-84
Cluster-6720.0	Psi-yw-159320	NRT1 PTR FAMILY -like	-9,445722704	7,040760E-44
Cluster-22812.24648	Psi-yw-186727	(R,S)-reticuline 7-O-methyltransferase	-9,351891967	1,784500E-113
Cluster-22812.25359	Psi-yw-280850	O-methyltransferase / Dimerisation domain	-9,343517158	2,480532E-81
Cluster-22947.5	Psi-yw-121878	truncated transcription factor CAULIFLOWER A isoform X1	-9,22809104	1,042437E-131
Cluster-3327.0	Psi-yw-272778	Helix-loop-helix DNA-binding domain	-9,218829014	1,579111E-131
Cluster-3079.8	Psi-yw-175845	alpha-expansin 1	-9,204054696	2,218718E-48
Cluster-22812.16869	Psi-yw-192838	tify domain / Divergent CCT motif	-9,178164816	1,822037E-52
Cluster-1200.6	Psi-yw-240725	SRF-type transcription factor (DNA-binding and dimerisation domain) / K-box region	-8,98860625	8,034518E-53
Cluster-22812.4531	Psi-yw-280852	(R,S)-reticuline 7-O-methyltransferase	-8,631108588	7,544352E-174

Cluster	Unigenes	Annotation	log2FoldChange	padj
Cluster-22812.12783	Psi-yw-105308	zinc finger 2	-8,399089653	7,022210E-76
Cluster-3254.6	Psi-yw-147382	21 kDa	-8,290059463	4,102816E-45
Cluster-13869.4	Psi-yw-289723	flavonoid 3 -monooxygenase	-8,269215475	5,682057E-46
Cluster-3445.3	Psi-yw-293875	K-box region	-8,190212242	9,753979E-143
Cluster-22812.8855	Psi-yw-290790	aldehyde dehydrogenase family 2 member mitochondrial	-8,168187354	2,527681E-90
Cluster-22812.9017	Psi-yw-246081	very-long-chain 3-oxoacyl- reductase 1	-8,084554617	2,426582E-44
Cluster-22812.9517	Psi-yw-191033	(R,S)-reticuline 7-O-methyltransferase	-7,975630252	2,134361E-175
Cluster-10125.5	Psi-yw-212035	asparagine synthetase [glutamine-hydrolyzing] 1	-7,939005371	1,279715E-74
Cluster-4157.1	Psi-yw-70264	wall-associated receptor kinase-like 1	-7,836662441	3,376128E-67
Cluster-6470.0	Psi-yw-279094	Protease inhibitor/seed storage/LTP family	-7,738523649	3,702959E-46
Cluster-10125.6	Psi-yw-277029	asparagine synthetase [glutamine-hydrolyzing] 1	-7,544350328	1,116942E-125
Cluster-22812.5519	Psi-yw-296602	adenine phosphoribosyltransferase 5	-7,386613003	1,511930E-45
Cluster-3601.6	Psi-yw-278223	aluminum-activated malate transporter 4	-7,345636166	1,308441E-151
Cluster-22812.5886	Psi-yw-148866	hypothetical protein CCACVL1_24695	-7,276539009	9,105955E-113
Cluster-3257.5	Psi-yw-278614	beta-glucosidase 3B-like	-7,191731011	3,451572E-50
Cluster-5897.0	Psi-yw-148571	uncharacterized protein LOC104443102	-7,180222177	3,316378E-42
Cluster-13454.4	Psi-yw-13877	WRKY DNA -binding domain	-7,146026071	2,283842E-48
Cluster-4186.7	Psi-yw-16525	MATH domain and coiled-coil domain-containing At3g58360-like	-7,025979421	7,025474E-65
Cluster-4186.8	Psi-yw-182319	MATH domain and coiled-coil domain-containing At3g58360-like	-6,982183372	1,211364E-61
Cluster-6532.0	Psi-yw-111338	No recognize	-6,932891937	6,703278E-43
Cluster-4186.6	Psi-yw-233870	MATH domain and coiled-coil domain-containing At3g58360-like	-6,922455822	4,628256E-64
Cluster-22812.5538	Psi-yw-96891	Phosphoribosyl transferase domain	-6,918335062	1,497581E-48
Cluster-7726.1	Psi-yw-210442	Heavy-metal-associated domain	-6,896933846	1,652628E-109
Cluster-6532.5	Psi-yw-287755	21 kDa -like	-6,883314875	7,364022E-68
Cluster-2495.2	Psi-yw-253929	Protein of unknown function DUF260	-6,864699005	2,303882E-50

Cluster	Unigenes	Annotation	log2FoldChange	padj
Cluster-4186.4	Psi-yw-233871	MATH domain and coiled-coil domain-containing At3g58360-like	-6,852876689	2,662256E-66
Cluster-6373.2	Psi-yw-199185	monosaccharide-sensing 2	-6,658013976	2,075385E-133
Cluster-3749.0	Psi-yw-221965	GDP-fucose protein O-fucosyltransferase	-6,602105185	3,702959E-46
Cluster-6532.6	Psi-yw-111340	21 kDa -like	-6,486013317	2,217725E-71
Cluster-5631.0	Psi-yw-218132	Yippee zinc-binding/DNA-binding /Mis18, centromere assembly	-6,438574895	2,083098E-56
Cluster-22812.806	Psi-yw-85835	plasma membrane-associated cation-binding 1	-6,352428559	4,675573E-44
Cluster-4279.4	Psi-yw-229024	aquaporin PIP1-2	-6,339653622	1,010788E-54
Cluster-6532.4	Psi-yw-111343	21 kDa -like	-6,286875628	2,682945E-82
Cluster-6532.9	Psi-yw-287757	Plant invertase/pectin methylesterase inhibitor	-6,217096203	3,937320E-43
Cluster-17170.0	Psi-yw-245868	Cellulase (glycosyl hydrolase family 5)	-6,185154169	2,422935E-51
Cluster-3601.5	Psi-yw-129700	aluminum-activated malate transporter 4	-6,178128837	1,698953E-58
Cluster-1254.0	Psi-yw-271153	C2 domain	-6,144685348	4,568022E-64
Cluster-6532.1	Psi-yw-287752	No recognize	-6,11405721	7,306475E-51
Cluster-22812.7521	Psi-yw-106656	AP2 domain	-5,989829512	8,303535E-74
Cluster-4889.1	Psi-yw-208505	BRO1-like domain	-5,964549487	7,022210E-76
Cluster-22024.0	Psi-yw-111925	Protein of unknown function (DUF1399)	-5,76063063	2,243521E-49
Cluster-18174.0	Psi-yw-142215	CSC1 At1g32090	-5,69682621	6,902070E-42
Cluster-10871.1	Psi-yw-224823	Protein of unknown function (DUF1635)	-5,668289291	3,895085E-72
Cluster-4896.7	Psi-yw-222438	Methyltransferase domain / Sterol methyltransferase C-terminal	-5,629953334	5,852919E-47
Cluster-11047.0	Psi-yw-269864	beta-amyrin synthase	-5,593535683	1,843353E-51
Cluster-22812.890	Psi-yw-83071	microtubule-associated 70-1	-5,555964305	6,566789E-65
Cluster-18567.0	Psi-yw-121787	homeobox knotted-1-like 1	-5,536030889	6,566789E-65
Cluster-22812.26111	Psi-yw-155456	ethylene-responsive transcription factor ERF061	-5,491806051	5,476351E-87
Cluster-22812.27154	Psi-yw-258451	endoglucanase 6	-5,404005176	4,758264E-45
Cluster-17170.1	Psi-yw-186660	Cellulase (glycosyl hydrolase family 5)	-5,373678448	9,808123E-65
Cluster-9085.0	Psi-yw-101925	uncharacterized protein LOC104432562 isoform X2	-5,34764482	3,932886E-65

Cluster	Unigenes	Annotation	log2FoldChange	padj
Cluster-18048.0	Psi-yw-47064	Senescence regulator	-5,321033837	5,116702E-74
Cluster-13695.0	Psi-yw-171764	uncharacterized protein LOC104419162	-5,166013646	1,049155E-46
Cluster-15184.0	Psi-yw-179663	branched-chain-amino-acid aminotransferase 1	-5,162848457	3,699810E-76
Cluster-1360.1	Psi-yw-32791	Domain of unknown function (DUF4228)	-5,068594745	5,287907E-53
Cluster-3842.15	Psi-yw-294642	1-aminocyclopropane-1-carboxylate oxidase 1	-4,954182487	1,624194E-92
Cluster-19419.0	Psi-yw-248636	Transmembrane amino acid transporter protein	-4,763300701	3,401438E-47
Cluster-22812.21197	Psi-yw-299335	unnamed protein product	-4,541239813	1,657087E-52
Cluster-15184.1	Psi-yw-126542	branched-chain-amino-acid aminotransferase 1	-4,431993068	8,491218E-141
Cluster-22812.24635	Psi-yw-82866	Permease family	-4,413683484	2,837887E-74
Cluster-4236.36	Psi-yw-64671	peroxisomal (S)-2-hydroxy-acid oxidase	-4,291656645	5,273116E-48
Cluster-22812.26060	Psi-yw-64341	ethylene-responsive transcription factor ERF061	-4,266522538	1,207200E-50
Cluster-22812.18881	Psi-yw-136383	dehydrin ERD14-like	-4,057061416	3,842908E-43
Cluster-7089.16	Psi-yw-106860	glycine dehydrogenase (decarboxylating) mitochondrial	4,136878005	2,740258E-42
Cluster-22812.10714	Psi-yw-227945	Retrovirus-related Pol poly from transposon TNT 1-94	4,372279494	8,273820E-57
Cluster-12116.1	Psi-yw-159469	ferredoxin-dependent glutamate chloroplastic	4,52999824	1,034949E-44
Cluster-16035.1	Psi-yw-286366	ELF4-LIKE 3-like	4,856091746	3,114091E-48
Cluster-22812.24590	Psi-yw-47840	No recognize	5,078715285	1,397674E-51
Cluster-7011.20	Psi-yw-119510	serine carboxypeptidase-like 2 isoform X1	5,160498191	1,468106E-57
Cluster-22812.11608	Psi-yw-143355	Tetratricopeptide repeat-like superfamily	5,194589537	1,248098E-49
Cluster-21047.0	Psi-yw-100548	SBF-like CPA transporter family (DUF4137)	5,411737158	6,084145E-55
Cluster-22812.20306	Psi-yw-277986	rust resistance kinase Lr10	5,459472695	1,029117E-60
Cluster-10475.22	Psi-yw-135925	subtilisin-like protease	5,523017032	7,804654E-57
Cluster-7659.34	Psi-yw-283561	pleiotropic drug resistance 2	5,593007849	4,156768E-82
Cluster-22812.11614	Psi-yw-231560	Tetratricopeptide repeat-like superfamily	5,627006286	2,382858E-48
Cluster-7731.0	Psi-yw-177007	proline-rich receptor-like protein kinase PERK9	5,835185702	5,466942E-89
Cluster-13333.6	Psi-yw-242233	NRT1 PTR FAMILY	5,935810196	2,582637E-42
Cluster-4550.2	Psi-yw-206392	No apical meristem (NAM) protein	6,144753817	1,150594E-59

Cluster	Unigenes	Annotation	log2FoldChange	padj
Cluster-22812.23590	Psi-yw-13734	(-)-germacrene D synthase	7,108922407	1,878046E-60
Cluster-16255.0	Psi-yw-253106	NRT1 PTR FAMILY	8,005126897	8,864270E-42
Cluster-22812.31999	Psi-yw-151271	aspartyl protease family At5g10770	8,858370815	4,788312E-43
Cluster-13737.8	Psi-yw-278432	galactinol synthase 1	9,856856968	6,919479E-57
Cluster-22812.13979	Psi-yw-278433	galactinol synthase 1	10,51174337	2,904617E-69
Cluster-22812.13980	Psi-yw-193688	galactinol synthase 1	10,61915214	5,156530E-45
