# Supplementary material for: Transcriptomics analysis of Psidium cattleyanum Sabine (Myrtaceae) unveil potential genes involved in fruit pigmentation
Source: Genet Mol Biol. 2020 Apr 27;43(2):e20190255. doi: 10.1590/1678-4685-GMB-2019-0255 (PMC7199922; doi:10.1590/1678-4685-GMB-2019-0255)
Supplement: Table S9 [file 1415-4757-GMB-43-2-e20190255-s10.pdf]

Supplementary material to: Transcriptomics analysis of *Psidium cattleyanum* Sabine (Myrtaceae) unveil potential genes involved in fruit pigmentation

Table S9 - Top 100 differential gene expression between Leaf vs Ripe fruit in yellow morphotype.

Cluster	Unigenes	Annotation	log2FoldChange	padj
Cluster-397.12	Psi-yw-285440	germin subfamily 1 member 7	-12.7152081868	1.78870872136079E-140
Cluster-22812.24648	Psi-yw-186727	(R,S)-reticuline 7-O-methyltransferase	-12.0904822779	1.3639278590605E-201
Cluster-3991.13	Psi-yw-285717	Senescence-associated gene	-12.0882447427	3.20105711862865E-102
Cluster-22812.25359	Psi-yw-280850	(R,S)-reticuline 7-O-methyltransferase	-11.6912734169	1.95216662139548E-133
Cluster-22812.4531	Psi-yw-280852	(R,S)-reticuline 7-O-methyltransferase	-11.4137573185	0,00000000E+00
Cluster-3991.10	Psi-yw-149533	Late embryogenesis abundant protein	-11.3757279264	2.15105429785811E-176
Cluster-3991.1	Psi-yw-285715	protein SENESCENCE-ASSOCIATED GENE 21, mitochondrial-like	-11.1877359296	5.93429012881966E-189
Cluster-20542.2	Psi-yw-46751	1-aminocyclopropane-1-carboxylate synthase 1	-11.1436389013	1.03357195399527E-120
Cluster-3254.4	Psi-yw-290163	Plant invertase/pectin methylesterase inhibitor	-11.0568279435	1.47800485893305E-109
Cluster-3991.4	Psi-yw-108155	SENESCENCE-ASSOCIATED GENE mitochondrial-like	-10.9517218139	2.77564326615588E-216
Cluster-22812.9517	Psi-yw-191033	(R,S)-reticuline 7-O-methyltransferase	-10.8947936665	0,00000000E+00
Cluster-3254.10	Psi-yw-147381	21 kDa	-10.8455130409	7.85134001396825E-162
Cluster-612.11	Psi-yw-138306	pathogenesis-related PR-4-like	-10.4529786694	1.70299231501535E-101
Cluster-865.5	Psi-yw-237737	cytochrome P450 81F3-like	-10.3328605491	1.7714611789908E-112
Cluster-22947.5	Psi-yw-121878	truncated transcription factor CAULIFLOWER A isoform X1	-10.2304833317	1.72095501914433E-171
Cluster-3327.0	Psi-yw-272778	Helix-loop-helix DNA-binding domain	-9.9187516848	2.83912033565399E-167
Cluster-2495.2	Psi-yw-253929	Protein of unknown function DUF260	-9.8237361061	8.64453756841595E-111

Cluster	Unigenes	Annotation	log2FoldChange	padj
Cluster-22812.8855	Psi-yw-290790	aldehyde dehydrogenase family 2 member mitochondrial	-9.8010471876	1.38304682424956E-146
Cluster-22812.12783	Psi-yw-105308	zinc finger 2	-9.6724921139	2.09151058986059E-109
Cluster-14047.1	Psi-yw-33362	uncharacterized protein LOC104437075	-9.5499766802	4.81586867044141E-104
Cluster-8824.0	Psi-yw-257977	Transmembrane amino acid transporter protein	-9.3771937171	4.24957743901079E-122
Cluster-8606.21	Psi-yw-293334	allene oxide cyclase	-8.9402632171	3.20105711862865E-102
Cluster-6373.2	Psi-yw-199185	monosaccharide-sensing 2	-8.5781448224	2.12371377249145E-254
Cluster-3601.6	Psi-yw-278223	aluminum-activated malate transporter 4	-8.4956644463	1.35863118461476E-220
Cluster-5631.0	Psi-yw-218132	Yippee zinc-binding/DNA-binding /Mis18, centromere assembly	-8.4288965688	4.93654764978461E-109
Cluster-3601.5	Psi-yw-129700	aluminum-activated malate transporter 4	-8.1349791351	6.63823179256293E-110
Cluster-10841.10	Psi-yw-41152	dof zinc finger -like	-8.1075261781	1.54229111244854E-102
Cluster-19419.0	Psi-yw-248636	Transmembrane amino acid transporter protein	-8.0445096888	9.93134079533687E-161
Cluster-7726.1	Psi-yw-210442	Heavy-metal-associated domain	-7.8873252808	5.59210616436672E-175
Cluster-18536.1	Psi-yw-104354	inactive cadmium zinc-transporting ATPase HMA3	-7.8593002861	6.78428620723806E-112
Cluster-4889.1	Psi-yw-208505	BRO1-like domain	-7.6917956451	2.49699141438115E-154
Cluster-22812.5886	Psi-yw-148866	hypothetical protein CCACVL1_24695	-7.5417649716	6.02895608856858E-140
Cluster-22812.21197	Psi-yw-299335	No recognize	-7.4742188208	3.38536088629299E-175
Cluster-7089.38	Psi-yw-191839	Lecithin retinol acyltransferase	-7.3352014372	6.07661723054991E-173
Cluster-22812.14759	Psi-yw-299333	protein NETWORKED 4A-like	-7.3209487557	9.640259186866E-149
Cluster-7089.17	Psi-yw-284053	Phosphopantetheine attachment site	-7.2129983632	3.35816093138882E-141
Cluster-22812.6949	Psi-yw-114395	No recognize	-7.1781207149	2.19763201623923E-135
Cluster-22812.27354	Psi-yw-282624	oleoyl-acyl carrier thioesterase chloroplastic	-7.1300800222	1.29032407511959E-139
Cluster-7089.9	Psi-yw-284051	Phosphopantetheine attachment site	-7.0970411604	1.88712184973651E-176
Cluster-3842.15	Psi-yw-294642	1-aminocyclopropane-1-carboxylate oxidase 1	-7.0165992432	2.19744953598897E-229
Cluster-22812.31014	Psi-yw-236447	uncharacterized protein LOC104443968	-6.9457337242	1.37111442098434E-133
Cluster-22812.7521	Psi-yw-106656	AP2 domain	-6.9266977102	2.89720315564685E-112
Cluster-7089.39	Psi-yw-268345	Phosphopantetheine attachment site	-6.7842656822	5.07296143037167E-107

Cluster	Unigenes	Annotation	log2FoldChange	padj
Cluster-7089.36	Psi-yw-284043	acyl carrier chloroplastic	-6.7761860023	1.30627332404971E-112
Cluster-22812.890	Psi-yw-83071	microtubule-associated 70-1	-6.7066609036	4.47451934375037E-111
Cluster-22812.26111	Psi-yw-155456	ethylene-responsive transcription factor ERF061	-6.7028402689	2.87179353333306E-150
Cluster-3142.2	Psi-yw-247866	endochitinase EP3	-6.5157607558	9.51827697204898E-110
Cluster-10871.1	Psi-yw-224823	Protein of unknown function (DUF1635)	-6.4637200535	3.98582703054656E-117
Cluster-17170.1	Psi-yw-186660	Cellulase (glycosyl hydrolase family 5)	-6.4404938644	4.07972257162332E-116
Cluster-8591.2	Psi-yw-221827	Phosphopantetheine attachment site	-6.1894849507	2.92343559922193E-149
Cluster-8750.0	Psi-yw-16402	type I cytoskeletal 10	-5.9640175407	1.33296762154358E-137
Cluster-7791.5	Psi-yw-70943	stearoyl-[acyl-carrier-] 9- chloroplastic-like	-5.8580071943	7.65187754246372E-191
Cluster-22812.6177	Psi-yw-111916	Phosphoenolpyruvate carboxykinase	-5.7966340698	3.35816093138882E-141
Cluster-22812.3081	Psi-yw-157314	phosphoenolpyruvate carboxykinase [ATP]-like	-5.7285753103	1.83398871782996E-141
Cluster-21576.1	Psi-yw-254405	Enoyl-(Acyl carrier protein) reductase	-5.6195559721	7.85517138687032E-119
Cluster-3079.13	Psi-yw-283564	expansin-A8-like precursor	-5.5975128726	6.41123593905708E-104
Cluster-22812.26060	Psi-yw-64341	ethylene-responsive transcription factor ERF061	-5.5008663064	2.70123871925216E-101
Cluster-7791.4	Psi-yw-193537	A Chain The Crystal Structure Of The Complex Between Stearoyl Acyl Carrier Desaturase From Ricinus Communis (Castor Bean) And	-5.4636074458	1.25667794383142E-232
Cluster-22812.30727	Psi-yw-192230	phosphoenolpyruvate carboxykinase [ATP]	-5.4525000418	5.36138508939895E-168
Cluster-22812.24635	Psi-yw-82866	Permease family	-5.3811572604	2.07384719154037E-135
Cluster-22812.33144	Psi-yw-192233	phosphoenolpyruvate carboxykinase [ATP]	-5.3437566358	2.10247676557075E-127
Cluster-22812.10767	Psi-yw-273203	phosphoenolpyruvate carboxykinase [ATP]-like	-5.3312874996	1.96566914087545E-171
Cluster-22812.28168	Psi-yw-181884	Cysteine-rich TM module stress tolerance	-5.3015770043	4.22103845588836E-113
Cluster-22812.860	Psi-yw-111910	phosphoenolpyruvate	-5.1242124844	3.31716410393563E-115
Cluster-22812.24658	Psi-yw-209883	nucleobase-ascorbate transporter 6	-5.0874327281	5.73159342055794E-148
Cluster-17694.3	Psi-yw-128614	plastidic ATP ADP-transporter-like	-5.0774257519	2.03464149717144E-125
Cluster-20925.1	Psi-yw-235339	Protein of unknown function (DUF789)	-4.9925609293	1.60368302837999E-114
Cluster-17544.1	Psi-yw-142238	ACT domain	-4.9743961207	3.20113213643555E-106
Cluster-22812.24657	Psi-yw-209884	nucleobase-ascorbate transporter 6	-4.9508989897	2.0667746525211E-118

Cluster	Unigenes	Annotation	log2FoldChange	padj
Cluster-22812.32028	Psi-yw-3735	2OG-Fe(II) oxygenase superfamily / non-haem dioxygenase in morphine synthesis N-terminal	-4.9388793795	7.68312087589879E-110
Cluster-16442.1	Psi-yw-17887	ethanolamine-phosphate cytidylyltransferase isoform X1	-4.9361041714	2.77509042465544E-116
Cluster-12543.0	Psi-yw-297346	pyridoxal 5 -phosphate synthase-like subunit	-4.8870764075	8.44596564915309E-194
Cluster-17544.0	Psi-yw-247675	ACT domain	-4.8770567492	1.63566832614466E-125
Cluster-12652.0	Psi-yw-142	C2 domain	-4.7054925106	4.63622188440585E-102
Cluster-22812.172	Psi-yw-167922	Protein of unknown function, DUF538	-4.6478502506	3.54918894035004E-128
Cluster-10766.1	Psi-yw-35808	ras-related Rab11D	-4.6325368298	7.79769779340547E-102
Cluster-22812.173	Psi-yw-247654	Protein of unknown function, DUF538	-4.6247065386	6.06662751404885E-122
Cluster-10018.0	Psi-yw-24363	protease Do-like 14	-4.5969433229	1.71378564626033E-145
Cluster-10321.3	Psi-yw-247655	Protein of unknown function, DUF538	-4.5902598837	1.09327565802963E-156
Cluster-22917.0	Psi-yw-19994	Yippee zinc-binding/DNA-binding /Mis18, centromere assembly	-4.5340517407	5.34312240223414E-100
Cluster-14893.5	Psi-yw-279316	GcpE protein	-4.5157571531	9.5785327637328E-122
Cluster-22812.27444	Psi-yw-143547	1-phosphatidylinositol-3-phosphate 5-kinase FAB1C	-4.5043729544	6.33332900042403E-190
Cluster-22812.27427	Psi-yw-96414	1-phosphatidylinositol-3-phosphate 5-kinase FAB1C	-4.4899944255	3.08124511012352E-181
Cluster-10588.1	Psi-yw-225811	programmed cell death 4	-4.3600400502	1.27674217796461E-102
Cluster-13260.1	Psi-yw-54538	heavy metal-associated HMA	-4.3514032567	2.00711247542417E-110
Cluster-14990.2	Psi-yw-19858	Mitochondrial carrier protein	-4.2971884424	3.21784188002212E-121
Cluster-8250.0	Psi-yw-107304	Ubiquitin-like autophagy protein Apg12	-4.1238889774	1.20319569691628E-117
Cluster-22812.18875	Psi-yw-158979	dehydrin 2	-4.0596023848	4.95272048925322E-102
Cluster-14424.0	Psi-yw-288383	hypothetical protein EUGRSUZ_F03989	-4.0396318145	1.55820531514193E-100
Cluster-8178.1	Psi-yw-56472	Domain of unknown function (DUF4588)	-4.0210835217	3.47822905906813E-119
Cluster-11047.0	Psi-yw-269864	beta-amyrin synthase	-8.373203556	4.69714260269674E-134
Cluster-7081.8	Psi-yw-258680	Histidine phosphatase superfamily (branch 1)	-4.804802454	4.77636347483277E-127
Cluster-3254.20	Psi-yw-266844	21 kDa	-10.34101074	6.15673628960347E-136

Cluster	Unigenes	Annotation	log2FoldChange	padj
Cluster-22812.7114	Psi-yw-24829	2OG-Fe(II) oxygenase superfamily / non-haem dioxygenase in morphine synthesis N-terminal	6.098955665	7.15954326316282E-108
Cluster-4864.11	Psi-yw-30826	Glycosyl transferase family 90	4.5618929472	3.73758031608077E-117
Cluster-22812.27433	Psi-yw-143654	homeobox BEL1 homolog	4.7621531233	3.71695282060961E-103
Cluster-11397.0	Psi-yw-146651	hydroquinone glucosyltransferase	5.0346628802	7.37169292479209E-219
Cluster-10574.3	Psi-yw-208470	Alpha/beta hydrolase family	5.2682358613	1.94084456622131E-129
Cluster-7731.0	Psi-yw-177007	proline-rich receptor-like protein kinase PERK9	7.0281269337	3.15183523499121E-119
Cluster-20835.1	Psi-yw-235931	NRT1 PTR FAMILY	9.6205432656	1.49523181169413E-117
