# Supplementary material for: Transcriptomics analysis of Psidium cattleyanum Sabine (Myrtaceae) unveil potential genes involved in fruit pigmentation
Source: Genet Mol Biol. 2020 Apr 27;43(2):e20190255. doi: 10.1590/1678-4685-GMB-2019-0255 (PMC7199922; doi:10.1590/1678-4685-GMB-2019-0255)
Supplement: Table S10 [file 1415-4757-GMB-43-2-e20190255-s11.pdf]

Genetics and Molecular Biology 43, 2 (2020)

Supplementary material to: Transcriptomics analysis of *Psidium cattleyanum* Sabine (Myrtaceae) unveil potential genes involved in fruit pigmentation

Table S10 - Top 100 differential gene expression between Leaf vs Unripe fruit in red morphotype

Cluster	Unigenes	Annotation	log2FoldChange	padj
Cluster-2958.24561	Psi-rd-240469	expansin 4 family	-12,31783639	3,46048551E-167
Cluster-882.1	Psi-rd-268889	probable 9-cis-epoxycarotenoid dioxygenase chloroplastic	-12,18251971	3,88190940E-173
Cluster-2958.21568	Psi-rd-271456	DNA-damage-repair toleration DRT100-like	-12,05944698	3,93428684E-88
Cluster-4091.4	Psi-rd-269454	No recognize	-11,74630668	7,38180137E-92
Cluster-2741.0	Psi-rd-88462	transcription factor PRE5-like	-11,51975755	4,27785672E-103
Cluster-2958.24538	Psi-rd-226252	expansin-A8-like precursor	-10,94576202	7,57789992E-157
Cluster-19403.3	Psi-rd-239217	Gibberellin regulated protein	-10,85338187	1,15476122E-109
Cluster-6780.1	Psi-rd-106864	No recognize	-10,82925718	6,59439361E-146
Cluster-8773.0	Psi-rd-172243	No recognize	-10,78914208	3,57604596E-84
Cluster-899.1	Psi-rd-268423	21 kDa	-10,77077531	3,33384524E-91
Cluster-2794.2	Psi-rd-284208	No recognize	-10,68678503	1,53471852E-112
Cluster-1376.7	Psi-rd-236924	dof zinc finger	-10,67247771	2,34058778E-96
Cluster-4208.18	Psi-rd-234652	plasma membrane-associated cation-binding protein 2	-10,63176776	2,64181358E-84
Cluster-795.0	Psi-rd-243774	Plant protein 1589 of unknown function (A_thal_3526)	-10,62931385	2,60304575E-100
Cluster-693.17	Psi-rd-272882	isoflavone 3 -hydroxylase	-10,62514037	6,74736129E-132
Cluster-2958.24274	Psi-rd-294600	expansin 3	-10,50421451	2,22797693E-195
Cluster-972.4	Psi-rd-283089	polygalacturonase	-10,46034888	8,45054214E-105
Cluster-2958.24239	Psi-rd-115441	Pollen allergen / Rare lipoprotein A (RlpA)-like double-psi beta-barrel	-10,34217304	4,13813015E-99
Cluster-899.4	Psi-rd-76839	21 kDa	-10,23517487	2,82987602E-127

Cluster	Unigenes	Annotation	log2FoldChange	padj
Cluster-2210.11	Psi-rd-254768	Pectate lyase	-10,23356558	3,48708267E-143
Cluster-2958.2139	Psi-rd-270950	(R,S)-reticuline 7-O-methyltransferase	-10,1743965	1,02802602E-128
Cluster-899.16	Psi-rd-268420	21 kDa	-10,14912292	1,79119078E-228
Cluster-2958.9678	Psi-rd-270096	probable RNA-binding ARP1 isoform X2	-10,04989602	9,51368527E-92
Cluster-2958.23328	Psi-rd-294597	expansin 3	-9,999757578	1,48066264E-108
Cluster-2958.1631	Psi-rd-231317	omega-hydroxypalmitate O-feruloyl transferase	-9,993971061	2,78606159E-87
Cluster-2958.26748	Psi-rd-208895	Pectate lyase	-9,752205413	5,33275638E-127
Cluster-2958.12264	Psi-rd-280031	Glycosyl transferase family group 2	-9,671549573	1,59770151E-86
Cluster-2958.1894	Psi-rd-180356	MADS-box CMB1-like	-9,6181734	1,46972769E-112
Cluster-2958.18045	Psi-rd-124004	endoglucanase 6	-9,45962085	7,38459096E-99
Cluster-1441.0	Psi-rd-108391	aluminum-activated malate transporter 4	-9,098025009	5,51859331E-95
Cluster-1105.6	Psi-rd-159479	lipid transfer	-8,579556506	1,24867355E-83
Cluster-2958.26252	Psi-rd-274141	Pectate lyase	-8,513199006	2,05030929E-200
Cluster-1806.5	Psi-rd-265758	protein E6	-8,300287494	3,05041555E-98
Cluster-1105.2	Psi-rd-162061	lipid transfer precursor	-8,156088731	9,35982135E-123
Cluster-1244.5	Psi-rd-173010	hypothetical protein EUGRSUZ_E00515	-8,113438731	1,24746270E-103
Cluster-18407.6	Psi-rd-195692	ABC transporter G family member 25	-7,883235201	3,46803968E-99
Cluster-1105.18	Psi-rd-162059	lipid transfer	-7,846215194	1,66909736E-112
Cluster-1105.25	Psi-rd-270793	lipid transfer precursor	-7,69592614	3,23454939E-93
Cluster-593.12	Psi-rd-294992	hydroxymethylglutaryl- reductase	-7,695689114	4,29900424E-109
Cluster-3828.2	Psi-rd-265670	Senescence regulator	-7,62419266	5,81240241E-90
Cluster-18407.11	Psi-rd-247775	ABC transporter G family member 25	-7,474958785	2,57418639E-133
Cluster-2958.497	Psi-rd-88373	zinc-finger of the FCS-type, C2-C2	-7,356994848	2,57608529E-84
Cluster-1806.7	Psi-rd-61236	protein E6	-7,356659452	4,26935290E-123
Cluster-1105.8	Psi-rd-270792	non-specific lipid-transfer	-7,165070235	1,11259518E-127
Cluster-8355.0	Psi-rd-47473	nematode resistance -like HSPRO2	-7,135581384	1,33204918E-116
Cluster-8045.23	Psi-rd-114518	No recognize	-6,863220266	1,26080075E-105
Cluster-8560.2	Psi-rd-262398	Pathogenesis-related protein Bet v I family	-6,663089616	2,15845652E-86
Cluster-1821.2	Psi-rd-238979	monosaccharide-sensing 2	-6,575125629	6,72166972E-100

Cluster	Unigenes	Annotation	log2FoldChange	padj
Cluster-3631.9	Psi-rd-294650	omega-3 fatty acid chloroplastic-like	-6,16261457	1,68572221E-95
Cluster-8045.8	Psi-rd-58876	neurofilament medium polypeptide	-6,088116653	3,01802203E-83
Cluster-9704.0	Psi-rd-247199	ferredoxin-dependent glutamate chloroplastic	5,97034313	2,40888987E-106
Cluster-2958.29918	Psi-rd-15712	glycine dehydrogenase (decarboxylating) mitochondrial	6,582468717	1,24746270E-103
Cluster-19010.0	Psi-rd-23827	phosphatase 2C 57	6,714045108	4,97923902E-107
Cluster-18535.0	Psi-rd-159317	cytochrome b6-f complex iron-sulfur	6,927457808	9,99983897E-103
Cluster-14933.2	Psi-rd-184768	TSS isoform X1	6,993401056	4,21510919E-110
Cluster-2632.26	Psi-rd-224535	ATPase family associated with various cellular activities (AAA)	7,182848387	4,12818675E-95
Cluster-7678.21	Psi-rd-229589	chlorophyll a-b binding chloroplastic	7,314252593	1,21667036E-88
Cluster-9704.1	Psi-rd-247198	ferredoxin-dependent glutamate chloroplastic	7,524315009	1,81789708E-167
Cluster-19308.2	Psi-rd-54431	hypothetical protein EUGRSUZ_D01944	7,606151835	2,31560515E-102
Cluster-10033.18	Psi-rd-157349	chlorophyll a-b binding chloroplastic	7,806347245	9,60858835E-104
Cluster-851.23	Psi-rd-84311	pentatricopeptide repeat-containing At5g65560-like isoform X1	7,851253671	3,05713712E-94
Cluster-2958.23966	Psi-rd-248607	hypothetical protein EUGRSUZ_B030602, partial	7,890936676	1,12384631E-101
Cluster-20354.1	Psi-rd-5023	Serine mitochondrial	7,910810046	8,34031746E-89
Cluster-2958.10533	Psi-rd-124196	LOV domain-containing	7,920031999	1,76911124E-88
Cluster-7678.25	Psi-rd-297112	chlorophyll a b-binding type	7,947540413	5,47089526E-104
Cluster-20015.1	Psi-rd-58957	serine--glyoxylate aminotransferase	8,01332383	5,27411746E-116
Cluster-10033.15	Psi-rd-128444	chlorophyll a-b binding chloroplastic	8,225995295	1,00095184E-94
Cluster-9704.3	Psi-rd-81784	ferredoxin-dependent glutamate chloroplastic	8,266981598	1,69291906E-93
Cluster-20015.3	Psi-rd-58954	serine--glyoxylate aminotransferase	8,300934497	2,31630510E-94
Cluster-19605.0	Psi-rd-5165	plastocyanin	8,305917824	1,03672914E-148
Cluster-11305.10	Psi-rd-230442	photosystem I reaction center subunit chloroplastic	8,326175965	3,81433056E-85
Cluster-579.4	Psi-rd-15870	chlorophyll a-b binding chloroplastic	8,34239226	1,04757637E-122
Cluster-18935.2	Psi-rd-88284	ferric reduction oxidase chloroplastic	8,505512766	6,66936520E-83

Cluster	Unigenes	Annotation	log2FoldChange	padj
Cluster-14952.3	Psi-rd-175825	Ribulose bisphosphate carboxylase, small chain / Ribulose-1,5-bisphosphate carboxylase small subunit	8,56441859	4,57972025E-117
Cluster-20015.2	Psi-rd-288823	serine--glyoxylate aminotransferase	8,60039372	2,61654631E-87
Cluster-15858.8	Psi-rd-258635	probable carotenoid cleavage dioxygenase chloroplastic	8,616687764	1,48574305E-134
Cluster-12155.3	Psi-rd-233351	glyceraldehyde-3-phosphate dehydrogenase chloroplastic	8,623504106	8,80996721E-130
Cluster-19281.1	Psi-rd-233963	fructose-1,6- chloroplastic	8,633433574	1,89235414E-87
Cluster-15247.0	Psi-rd-190214	NAD dependent epimerase/dehydratase family	8,664598279	1,87379224E-87
Cluster-2958.25046	Psi-rd-6633	protein CHUP1, chloroplastic	8,669266749	6,92812348E-101
Cluster-18541.0	Psi-rd-255131	ferredoxin--NADP leaf chloroplastic	8,696979642	6,40863818E-115
Cluster-7678.32	Psi-rd-214041	chlorophyll a-b binding chloroplastic	8,85928358	3,42059844E-127
Cluster-14952.6	Psi-rd-253002	Ribulose bisphosphate carboxylase, small chain / Ribulose-1,5-bisphosphate carboxylase small subunit	9,149457989	3,79627515E-89
Cluster-14952.9	Psi-rd-165461	ribulose bisphosphate carboxylase small chain 4, chloroplastic-like	9,179685524	4,77274375E-100
Cluster-20354.6	Psi-rd-281054	serine mitochondrial	9,263285429	1,32070679E-97
Cluster-7627.2	Psi-rd-390	fructose-bisphosphate aldolase chloroplastic	9,275752947	2,24050405E-111
Cluster-21179.1	Psi-rd-23215	Light regulated protein Lir1	9,557314557	3,72938554E-142
Cluster-14952.40	Psi-rd-165459	Ribulose bisphosphate carboxylase, small chain / Ribulose-1,5-bisphosphate carboxylase small subunit	9,812381949	1,41307112E-98
Cluster-851.9	Psi-rd-233630	Phosphoribulokinase family	9,845244241	2,32068364E-97
Cluster-14952.35	Psi-rd-139791	Ribulose bisphosphate carboxylase, small chain / Ribulose-1,5-bisphosphate carboxylase small subunit	9,954336143	9,14124226E-127
Cluster-14952.21	Psi-rd-211411	Ribulose bisphosphate carboxylase, small chain / Ribulose-1,5-bisphosphate carboxylase small subunit	9,973386096	8,26689552E-113
Cluster-3996.3	Psi-rd-46782	peroxisomal (S)-2-hydroxy-acid oxidase	10,02536162	4,74570775E-112
Cluster-3996.4	Psi-rd-107889	peroxisomal (S)-2-hydroxy-acid oxidase	10,86159821	2,18053805E-129

Cluster	Unigenes	Annotation	log2FoldChange	padj
Cluster-2632.22	Psi-rd-280546	ATPase family associated with various cellular activities (AAA)	10,87769674	7,06512958E-98
Cluster-2958.20359	Psi-rd-204515	fructose-bisphosphate aldolase chloroplastic	10,89042692	1,68773252E-154
Cluster-2958.513	Psi-rd-260002	fructose-bisphosphate aldolase chloroplastic	11,04587602	4,23898013E-101
Cluster-2958.33205	Psi-rd-54839	fructose-bisphosphate aldolase chloroplastic	11,06280365	1,16615446E-111
Cluster-2632.36	Psi-rd-145549	ribulose bisphosphate carboxylase oxygenase chloroplastic	11,49327991	1,59027817E-186
Cluster-2632.32	Psi-rd-153474	ATPase family associated with various cellular activities (AAA)	11,49911835	6,97765583E-107
Cluster-2632.37	Psi-rd-280547	ATPase family associated with various cellular activities (AAA)	11,83608591	4,42307272E-102
